# Single-cell multiomic analysis of thymocyte development reveals drivers of CD4^+^ T cell and CD8^+^ T cell lineage commitment

**DOI:** 10.1038/s41590-023-01584-0

**Published:** 2023-08-14

**Authors:** Zoë Steier, Dominik A. Aylard, Laura L. McIntyre, Isabel Baldwin, Esther Jeong Yoon Kim, Lydia K. Lutes, Can Ergen, Tse-Shun Huang, Ellen A. Robey, Nir Yosef, Aaron Streets

**Affiliations:** 1 University of California, Berkeley, Department of Bioengineering, Berkeley, CA USA; 2 UC Berkeley - UCSF Graduate Program in Bioengineering, Berkeley and San Francisco, CA USA; 3 University of California, Berkeley, Center for Computational Biology, Berkeley, CA USA; 4 University of California, Berkeley, Division of Immunology and Molecular Medicine, Department of Molecular and Cell Biology, Berkeley, CA USA; 5 University of California, Berkeley, Department of Electrical Engineering and Computer Sciences, Berkeley, CA USA; 6 BioLegend, Inc., San Diego, CA USA; 7 Weizmann Institute of Science, Department of Systems Immunology, Rehovot, Israel; 8 Chan Zuckerberg Biohub - San Francisco, San Francisco, CA USA; 9 Present Address: Massachusetts Institute of Technology, Institute for Medical Engineering and Science, Cambridge, MA USA; 10 Present Address: Broad Institute of MIT and Harvard, Cambridge, MA USA; 11 Present Address: Ragon Institute of MGH, MIT, and Harvard, Cambridge, MA USA

**Keywords:** Thymus, RNA sequencing, Protein sequencing, T cells, Gene expression analysis

## Abstract

The development of CD4^+^ T cells and CD8^+^ T cells in the thymus is critical to adaptive immunity and is widely studied as a model of lineage commitment. Recognition of self-peptide major histocompatibility complex (MHC) class I or II by the T cell antigen receptor (TCR) determines the CD8^+^ or CD4^+^ T cell lineage choice, respectively, but how distinct TCR signals drive transcriptional programs of lineage commitment remains largely unknown. Here we applied CITE-seq to measure RNA and surface proteins in thymocytes from wild-type and T cell lineage-restricted mice to generate a comprehensive timeline of cell states for each T cell lineage. These analyses identified a sequential process whereby all thymocytes initiate CD4^+^ T cell lineage differentiation during a first wave of TCR signaling, followed by a second TCR signaling wave that coincides with CD8^+^ T cell lineage specification. CITE-seq and pharmaceutical inhibition experiments implicated a TCR–calcineurin–NFAT–GATA3 axis in driving the CD4^+^ T cell fate. Our data provide a resource for understanding cell fate decisions and implicate a sequential selection process in guiding lineage choice.

## Abstract (web summary)

Streets, Yosef, Robey and colleagues use multiomics analysis to generate a comprehensive timeline of the CD4^+^ and CD8^+^ T cell lineage commitment and identify sequential waves of TCR signaling that first initiate CD4^+^ T cell lineage differentiation and then CD8^+^ T cells lineage specification.

## Main

The commitment of a developing thymocyte to the CD4^+^ helper or CD8^+^ cytotoxic T cell fate provides an important model for understanding cell fate decisions. The ultimate fate of a thymocyte is determined by the specificity of its T cell receptor (TCR) for major histocompatibility complex (MHC) molecules during positive selection in the thymus, with recognition of major histocompatibility complex class I (MHCI) leading to the CD8^+^ T cell fate and recognition of MHC class II (MHCII) leading to the CD4^+^ T cell fate. CD8 and CD4 are coreceptors for MHCI and MHCII, respectively, and their expression pattern has an important role in lineage commitment^1–4^. The ‘kinetic signaling’ model of lineage commitment focuses on TCR signal termination in CD8-fated cells in directing the lineage choice^3^. However, there is evidence that TCR signaling impacts thymocyte development throughout the >2-day process of positive selection^5–9^, and a clear, quantitative picture of the temporal pattern of TCR signaling throughout lineage specification has not emerged yet. It is also unknown whether different transcriptional targets of the TCR pathway are activated in a temporal and/or lineage-specific manner. Thus, the molecular links between TCR signaling and induction of the lineage-defining transcription factors THPOK (encoded by *Zbtb7b* in mice) and RUNX3 in mature CD4^+^ and CD8^+^ T cells, respectively^10^ remain unknown.

One complicating factor in addressing these questions is the diversity in TCR specificity and resulting cell fates, with many cells undergoing death by neglect, negative selection or agonist selection, alongside CD4-fated and CD8-fated cells. Even in mice bearing fixed rearranged TCRs (TCR transgenic (TCRtg)) that lead to a predetermined lineage choice, defining cell states and ordering them into a developmental trajectory remains a challenge. Traditionally, cell states have been characterized using flow cytometry to quantify cell surface markers^1^. Defining immature CD4^+^CD8^+^ (double positive (DP)) versus mature CD4^+^CD8^−^ or CD4^−^CD8^+^ (single positive (SP)) is relatively straightforward; however, further subdivisions can be complicated by the subjectivity of manual gating and limited number of markers. This approach also lacks the ability to make quantitative global comparisons in gene expression between cell states.

Advances in single-cell RNA sequencing (scRNA-seq) technologies have enabled the unbiased observation of transcriptional heterogeneity in the mammalian thymus^11–15^. Although these studies provided important insights, they did not have sufficient temporal resolution of the CD4 versus CD8 commitment process to connect TCR signaling events to the induction of THPOK, RUNX3 and the initiation of lineage-specific transcriptional programs. A high-resolution delineation of the differentiation process that provides connections to flow cytometry-based studies is needed to inform models of lineage commitment and to identify early drivers of lineage divergence.

To address these challenges, we leveraged CITE-seq^16^ and totalVI^17^ to build a high-resolution timeline of RNA and protein expression changes during positive selection. We identified two temporally distinct waves of TCR signaling: an early wave that is more sustained in CD4-fated cells, and a later wave that is specific to CD8-fated cells and that overlaps with CD8^+^ T cell lineage specification. We find that CD8-fated cells initially undergo a parallel, but transient, CD4 transcriptional program, implying that CD8-fated cells audition for the CD4^+^ T cell fate before undergoing CD8^+^ T cell lineage specification. We also identify TCR signaling through calcineurin–NFAT as a driver of the CD4^+^ T cell fate. These data provide an important resource for understanding T cell fate commitment in the thymus.

## Results

### CITE-seq and totalVI form an RNA–protein thymocyte atlas

**Fig. 1:**
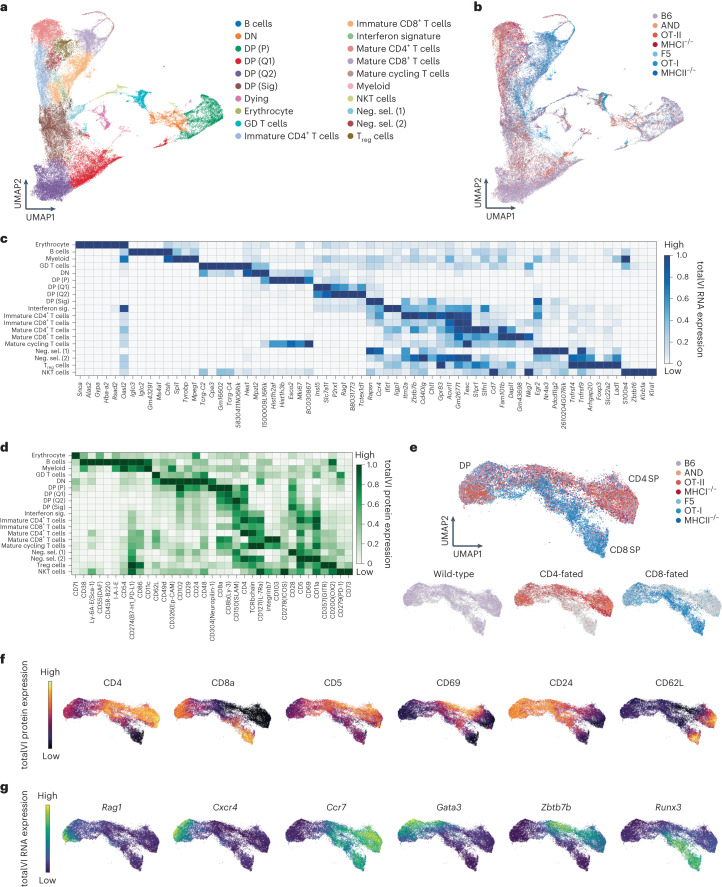
A joint transcriptomic and surface protein atlas of thymocyte development in wild-type and lineage-restricted mice. **a**,**b**, UMAP^51^ plots of the totalVI latent space from CITE-seq on total thymocytes from wild-type (WT), CD4-fated (MHCI^−/−^, OT-II and AND) and CD8-fated (MHCII^−/−^, OT-I and F5) mice, labeled by cell-type annotation (**a**) and mouse genotype (**b**). **c**,**d**, Heatmaps of markers derived from totalVI one-vs-all differential expression test between cell types for RNA (**c**) and proteins (**d**) in thymocytes from mice as in **a**. Values are totalVI denoised expression. **e**, UMAP plots of the totalVI latent space from positively selected thymocytes with cells labeled by mouse genotype. **f**, UMAP plots of the totalVI latent space from positively selected thymocytes. Cells colored by totalVI denoised expression of protein markers of lineage (CD4, CD8a), TCR signaling (CD5, CD69) and maturation (CD24, CD62L). **g**, Cells colored by totalVI denoised expression of RNA markers of TCR recombination (*Rag1*), thymic location (*Cxcr4*, *Ccr7*), and lineage regulation (*Gata3*, *Zbtb7b*, *Runx3*) as in **f**. GD T cells, gamma-delta T cells; DN, double negative; DP (P), double positive proliferating; DP (Q1), DP quiescent 1; DP (Q2), DP quiescent 2; DP (Sig.), DP signaled; Neg. sel. (1), negative selection 1; Neg. sel. (2), negative selection 2; T_reg_ cell, regulatory CD4^+^ T cell; NKT cell, natural killer T cell.

**Extended Data Fig. 1:**
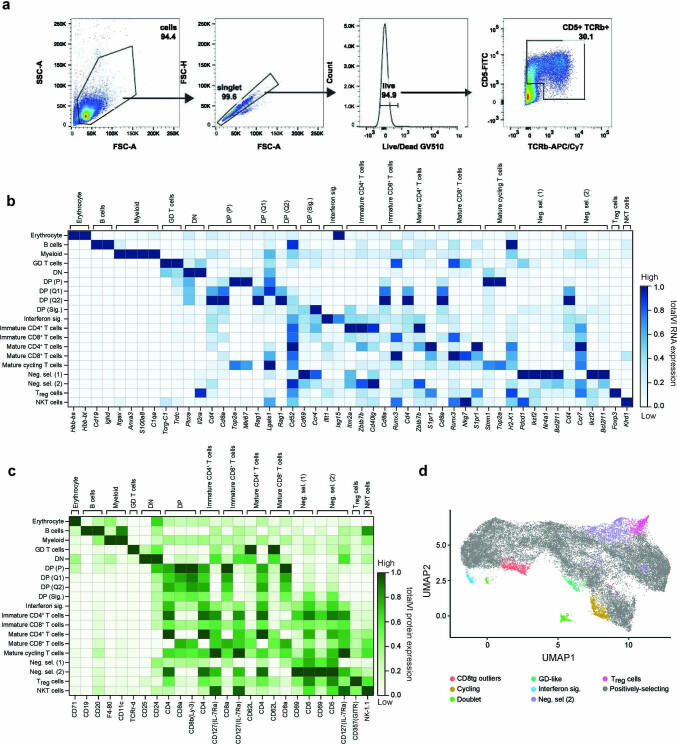
Sorting and characterization of positively selecting thymocytes. **a**, Representative FACS plots displaying gating strategy to sort thymocytes for CITE-seq. Cell populations were gated and sorted to include lymphocytes, exclude forward scatter doublets, include Ghost Dye Violet 510 Live/Dead stain negative (live cells), then on TCRβ^+^CD5^+^ to enrich for cells that were positively selecting. **b-c**, Heatmaps of manually selected cell-type markers for RNA (**b**) and proteins (**c**). Values are totalVI denoised expression. **d**, UMAP plot of totalVI latent space from positively selected thymocytes before filtering indicating annotated populations that were retained (positively selecting thymocytes) or removed (all other populations) from downstream analysis.

We profiled thymocytes from wild-type C57BL/6 mice (referred to as B6), CD4^+^ T cell lineage-restricted mice (hereafter CD4-fated), including AND and OT-II TCRtg mice, which express TCRs specific for MHCII, and *B2m*^−/−^ (referred to as MHCI^−/−^), which have diverse TCR repertoires, and CD8^+^ T cell lineage-restricted mice (hereafter CD8-fated), including F5 and OT-I TCRtg mice, which express TCRs specific for MHCI, and *H2-Ab1*^−/−^ (referred to as MHCII^−/−^), which have diverse TCRs. We measured transcriptomic and surface protein composition at the single-cell level using CITE-seq^16^ with a panel of 111 antibodies (Supplementary Data 1), which we jointly analyzed using totalVI^17^. We analyzed thymi from two mice per lineage-restricted genotype and five wild-type mice (Supplementary Data 2). Samples from the MHC-deficient mice and three of the five wild-type mice were sorted to enrich for CD5^+^TCRβ^+^ thymocytes undergoing positive selection (Extended Data Fig. 1a). totalVI integration of CITE-seq data (72,042 cells) stratified cells based on RNA and protein information (Fig. 1a) and identified thymocytes across developmental stages including CD4^−^CD8^−^ (double negative), proliferating DP, quiescent pre-selection DP, post-TCR-recombination DP receiving positive selection signals (DP (Sig.)), and immature and mature CD4^+^ and CD8^+^ T cells, along with negatively selecting and agonist-selecting populations (Fig. 1a). Wild-type, CD4-fated and CD8-fated samples were well mixed at early developmental stages, up to DP (Sig.) but branched into CD4^+^ and CD8^+^ T cell lineages in later-stage populations (Fig. 1b). We characterized cell populations with traditional markers (Extended Data Fig. 1b,c) and with unbiased totalVI differential expression tests (Fig. 1c,d and Supplementary Data 3). Top differentially expressed features included lineage surface markers (CD4, CD8), transcription factors (*Foxp3*, *Zbtb7b*) and markers of maturation stage (*Rag1*, *Ccr4*, *S1pr1*) (Fig. 1c,d). These multiomic definitions indicated continuous expression changes, particularly between the DP and SP stages (Fig. 1c,d), motivating analysis as a continuous developmental process.

We focused further analysis on positively selecting thymocytes from DP (Sig.) through mature stages (Fig. 1a). The totalVI latent space derived from these populations stratified thymocytes by developmental stage and CD4–CD8 lineage (Fig. 1e and Extended Data Fig. 1d). totalVI denoised expression of the proteins CD4 and CD8, markers of positive selection-induced TCR signaling (CD5, CD69) and maturation stage (CD24, CD62L), as well as RNA markers of TCR recombination (*Rag1*), location within the thymus (*Cxcr4*, *Ccr7*) and lineage regulation (*Gata3*, *Zbtb7b*, *Runx3*) showed the expected developmental and lineage-specific patterns^18^ (Fig. 1f,g). Thus, our data enabled a high-resolution analysis of the continuous developmental processes between the DP and SP stages.

### Pseudotime clarifies intermediate developmental stages

**Fig. 2:**
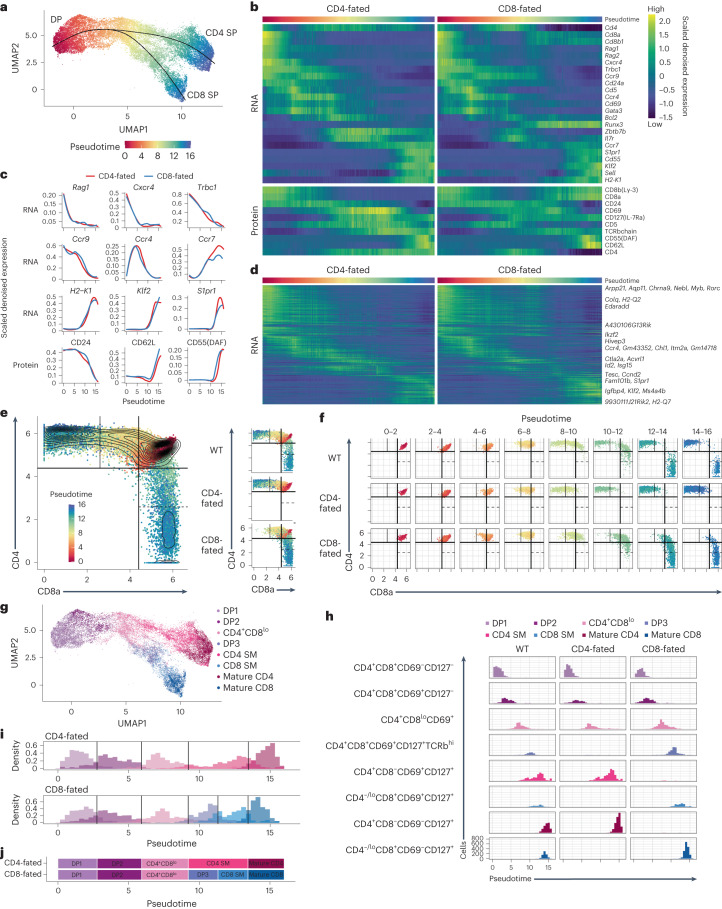
Pseudotime inference captures continuous maturation trajectory and clarifies intermediate thymocyte stages. **a**, UMAP plot of the totalVI latent space from positively selected thymocytes with cells colored by Slingshot pseudotime and smoothed curves representing the CD4^+^ and CD8^+^ T cell lineages. **b**, Heatmap of RNA (top) and protein (bottom) markers of thymocyte development over pseudotime in the CD4^+^ and CD8^+^ T cell lineages. Features are colored by totalVI denoised expression, scaled per row, and sorted by peak expression in the CD4^+^ T cell lineage. Pseudotime axis is the same as in **a**. **c**, Expression of features in the CD4^+^ and CD8^+^ T cell lineages that vary over pseudotime. Features are totalVI denoised expression values scaled per feature and smoothed by loess curves. **d**, Heatmap of all RNA differentially expressed over pseudotime in any lineage. Features are scaled and ordered as in **b**. Labeled genes are highly differentially expressed over time (Methods). **e**, In silico flow cytometry plots of log(totalVI denoised expression) of CD8a and CD4 from positively selected thymocytes (left) and the same cells separated by lineage (right). Cells are colored by pseudotime. Gates were determined based on contours of cell density. WT, wild-type. **f**, In silico flow cytometry plot of data as in **e** separated by lineage and pseudotime. **g**, UMAP plot of the totalVI latent space from positively selected thymocytes with cells colored by gate. Cells were computationally grouped into eight gates using CD4, CD8a, CD69, CD127(IL-7Ra), and TCRβ. **h**, Histograms of cells separated by lineage and gate with cells colored by gate as in **g**. **i**, Stacked histograms of gated populations in CD4-fated (top) and CD8-fated (bottom) thymocytes, with thresholds classifying gated populations over pseudotime (Methods). **j**, Schematic timeline aligns pseudotime with gated populations, with population timing determined as in **i**.

**Extended Data Fig. 2:**
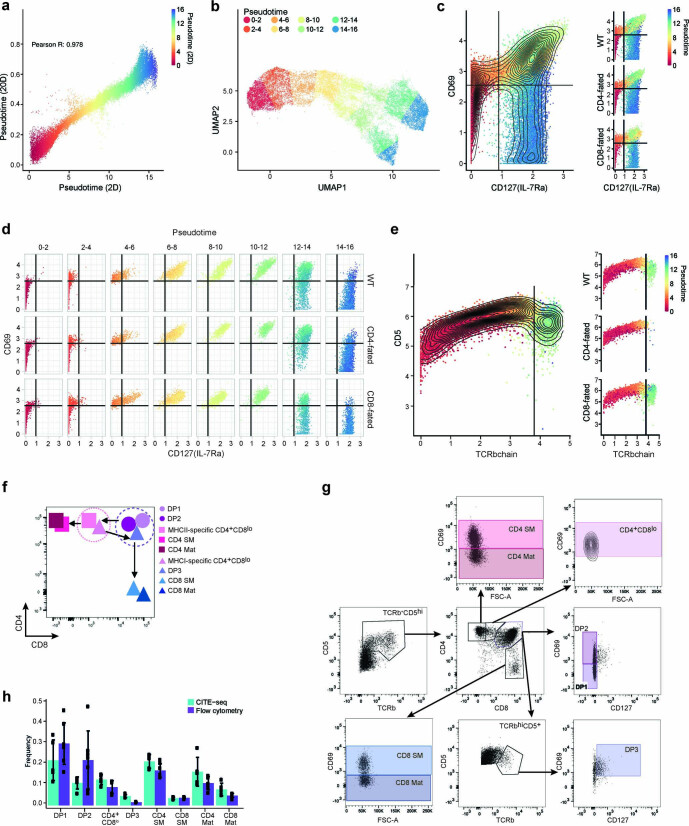
Pseudotime inference identifies intermediate thymocyte stages that can be identified by flow cytometry. **a**, Correlation between Slingshot pseudotime inferred from the full 20-dimensional totalVI latent space and a 2-dimensional UMAP projection of the 20-dimensional latent space. **b**, UMAP plot of the totalVI latent space from positively selected thymocytes. Cells are colored according to placement in eight bins uniformly spaced over 2D pseudotime for visualization. **c**, In silico flow cytometry plots of log(totalVI denoised expression) of CD127(IL-7Ra) and CD69 from positively selected thymocytes (left) and the same cells separated by lineage (right). Cells are colored by pseudotime. **d**, Data as in **c** separated by lineage and pseudotime. **e**, In silico flow cytometry plots of log(totalVI denoised expression) of TCRβ and CD5 from DP thymocytes (left) and the same cells separated by lineage (right). Cells are colored by pseudotime. Among DP thymocytes, the DP3 population is TCRβ^hi^, CD127^+^ and CD69^+^. **f**, Schematic of CD4 versus CD8 biaxial plot to identify eight gated populations in adult thymocytes: DP1, DP2, CD4^+^CD8^lo^, semimature CD4 (CD4 SM), mature CD4 (CD4 Mat), DP3, semimature CD8 (CD8 SM), and mature CD8 (CD8 Mat). Circles represent lineage uncommitted cells, squares represent CD4^+^ T lineage cells, and triangles represent CD8^+^ T lineage cells. **g**, Representative flow cytometry gating strategy for thymocyte populations in adult mice. Thymocytes were harvested from 6-8-week-old wild-type, MHCI^-/-^ or MHCII^-/-^ mice. Cell populations were gated to include lymphocytes, exclude forward scatter and side scatter doublets, include live cells, include TCRβ^+^CD5^int/hi^, then on CD4 versus CD8. Cell populations were gated into the following subsets based upon cell surface marker expression: DP1 (CD4^+^CD8^+^CD127^−^CD69^−^), DP2 (CD4^+^CD8^+^CD127^−^CD69^+^), CD4^+^CD8^lo^ (CD4^+^CD8^lo^; CD4^+^CD8^lo^CD69^+^), DP3 (CD4^+^CD8^+^TCRβ^hi^CD5^+^CD127^+^CD69^+^), semimature CD4 (CD4 SM; CD4^+^CD8^−^CD69^+^), mature CD4 (CD4 Mat; CD4^+^CD8^−^CD69^−^), semimature CD8 (CD8 SM; CD8^+^CD69^+^), and mature CD8 (CD8 Mat; CD8^+^CD69^−^). **h**, Cell-type frequencies for the eight gated populations defined by CITE-seq as in Fig. 2g, h and by flow cytometry as in **f-g**. Frequencies were derived from the five wild-type mice in the CITE-seq data set and seven wild-type mice for flow cytometry. Error bars denote mean ± standard deviation.

Next, we performed pseudotime inference with Slingshot^19,20^ on the joint RNA-protein reduced dimension space to delineate the expression changes throughout positive selection along a branching trajectory (Fig. 2a, Extended Data Fig. 2a and Supplementary Data 4). The inferred pseudotime captured the timing of known expression changes^18^, such as early downregulation of *Rag1*, continuous downregulation of the early markers *Ccr9* and *Cd24a/*CD24, transient expression of *Cd69*/CD69 and late upregulation of the maturation markers *Klf2*, *S1pr1* and *Sell/*CD62L (Fig. 2b,c). To explore beyond known markers, we performed totalVI differential expression tests over pseudotime, and created a comprehensive timeline of RNA and protein expression changes for each lineage (Fig. 2d, Supplementary Data 5 and 6 and Supplementary Information). The lineages differed in their expression of key molecules, such as coreceptors and transcription factors (Fig. 2b), as expected, whereas markers of maturation followed similar trajectories in both lineages (Fig. 2c). Most significant differences over time were common across lineages (Fig. 2d), which enabled the investigation of lineage-specific differences at comparable developmental stages.

Owing to the continuous nature of development and technical variations in marker detection, no consensus has emerged on how to define positive selection intermediates by flow cytometry^1,2,21,22^. To address this, we performed in silico flow cytometry on totalVI denoised surface protein expression to distinguish different pseudotime phases. CD4-fated cells progressed continuously in pseudotime from DP to CD4^+^CD8^lo^ to CD4^+^CD8^-^ (Fig. 2e,f and Extended Data Fig. 2b), whereas CD8-fated cells progressed from DP to CD4^+^CD8^lo^ before reversing course to reach CD4^−^CD8^+^^23–25^. Specifically, although at pseudotime 6–8 nearly all CD8-fated cells were CD4^+^CD8^lo^, at pseudotime 8–12, CD8-fated, but not CD4-fated, thymocytes passed through a DP phase again (referred to as DP3) (Fig. 2f). Although a later-time MHCI-specific DP3 population has been reported^21^, it is not commonly accounted for^11,14,15^, resulting in the contamination of the DP gate with later-time CD8-fated cells.

We used a data-driven strategy to identify a minimal set of surface markers capturing intermediate stages of positive selection and to better characterize the DP3 stage. Four pseudotime stages were separated by in silico gating on CD69 and CD127(IL-7Ra) independent of lineage: CD69^lo^CD127^lo^, CD69^+^CD127^lo^, CD69^+^CD127^+^ and CD69^−^CD127^+^ (Extended Data Fig. 2c,d). Addition of CD4 and CD8 markers enabled distinction between the lineages at later times (Fig. 2g,h). DP3 had high expression of TCRβ^21,26^ and was CD69^+^CD127^+^ (Extended Data Fig. 2d,e), compared to earlier CD69^−^CD127^−^ DP1 and CD69^+^CD127^−^ DP2. In combination, a gating scheme using CD4, CD8, TCRβ, CD69 and CD127 resolved eight populations (DP1, DP2, CD4^+^CD8^lo^, DP3, semimature CD4 (CD4 SM) and mature CD4 (which combined corresponded to CD4 SP), and semimature CD8 (CD8 SM) and mature CD8 (which combined corresponded to CD8 SP)) (Fig. 2g,h) and could enable the approximation of this separation by time and lineage using flow cytometry (Fig. 2i,j). Fluorescence-based flow cytometry replicated these eight CITE-seq-derived gates, supporting the presence of the proposed intermediate stages (Extended Data Fig. 2f–h). Collectively, these observations specified an updated model of positive selection intermediates in the CD4^+^ and CD8^+^ T cell lineages.

### CITE-seq reveals order of key lineage commitment events

**Fig. 3:**
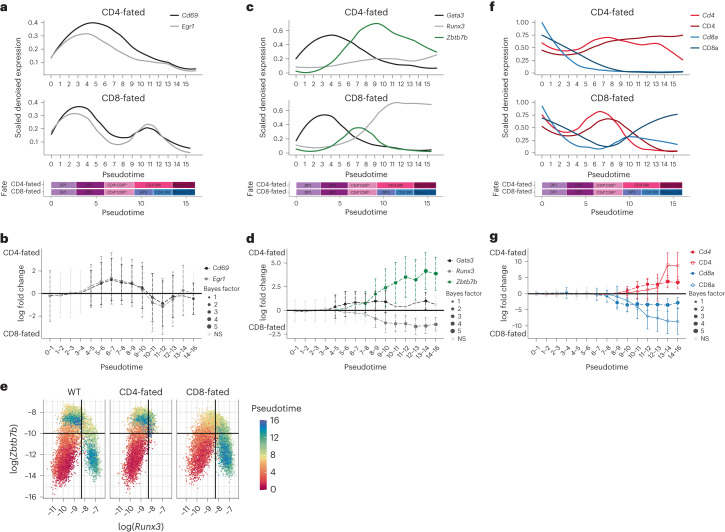
Paired measurements of RNA and protein reveal the timing of major events in CD4-CD8 lineage commitment. **a**, Expression over pseudotime of TCR signaling response molecules. Features are totalVI denoised expression values scaled per feature and smoothed by loess curves. Schematic timeline below the plot aligns pseudotime with gated populations (see Fig. 2j). **b**, Differential expression over pseudotime between CD4-fated and CD8-fated thymocytes for RNA of TCR signaling response molecules. Non-significant (NS) differences are gray, significant differences are filled circles. Size of the circle indicates log(Bayes factor). Data are median log fold change ± totalVI-computed s. d. **c**, Expression over pseudotime as in **a** for RNA of key transcription factors. **d**, Differential expression over pseudotime as in **b** for RNA of key transcription factors. **e**, In silico flow cytometry plots of log(totalVI denoised expression) of *Runx3* and *Zbtb7b* from positively selected thymocytes separated by lineage and colored by pseudotime. **f**, Expression over pseudotime as in **a** for coreceptor RNA and protein. **g**, Differential expression over pseudotime as in **b** for RNA of coreceptors. Significant RNA results are filled circles and significant protein results are open circles. **b**,**d**,**g**, *n* = 9,545 CD4-fated cells (MHCI^−/−^, OT-II and AND mice) and 9,126 CD8-fated cells (MHCII^−/−^, OT-I and F5 mice).

**Extended Data Fig. 3:**
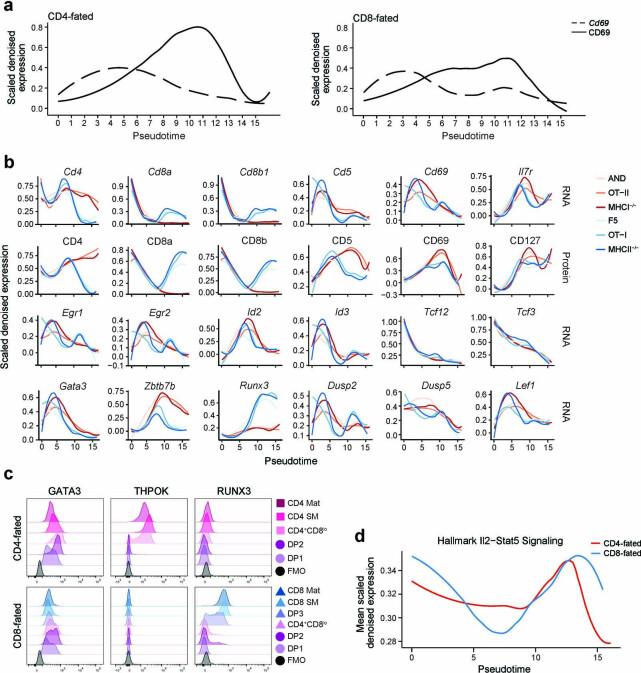
CITE-seq and fluorescence-based flow cytometry reveal the timing of expression for transcription factors and other features of CD4-CD8 T cell development. **a**, Expression over pseudotime of *Cd69* (dashed) and CD69 (solid). Features are totalVI denoised expression values scaled per feature and smoothed by loess curves. **b**, Expression of RNA and protein features over pseudotime by genotype. Features are totalVI denoised expression values scaled per feature and smoothed by loess curves. A resource of protein and RNA expression over pseudotime for all differentially expressed features is in Supplementary Information. **c**, Transcription factor protein expression in adult thymocyte populations. Representative histograms displaying GATA3, THPOK, and RUNX3 transcription factor expression detected by intracellular flow cytometry staining in CD4-fated (MHCI^-/-^) and CD8-fated (MHCII^-/-^) thymocyte populations. Thymocyte populations were gated on lymphocytes, excluding forward scatter and side scatter doublets, live cells, TCRβ^+^CD5^int/hi^ then on CD4 versus CD8. Cell populations were gated into the following subsets based upon cell surface marker expression: DP1 (CD4^+^CD8^+^CD127^−^CD69^−^), DP2 (CD4^+^CD8^+^CD127^−^CD69^+^), DP3 (CD4^+^CD8^+^TCRβ^hi^CD5^+^CD127^+^CD69^+^), CD4^+^CD8^lo^ (CD4^+^CD8^lo^CD69^+^), semimature CD4 (CD4 SM, CD4^+^CD8^−^CD69^+^), mature CD4 (CD4 Mat, CD4^+^CD8^−^CD69^−^), semimature CD8 (CD8 SM, CD8^+^CD69^+^), and mature CD8 (CD8 Mat, CD8^+^CD69^−^). Data is concatenated from n = 4 mice per genotype. Positive staining was determined using a fluorescence minus one control. **d**, Expression over pseudotime of the Hallmark Il2-Stat5 Signaling signature^74^ displayed as the mean of scaled totalVI denoised expression per gene, smoothed by loess curves.

Prolonged TCR signaling is known as a driver of CD4^+^ T cell lineage commitment, whereas the role of TCR signals in CD8 SP development remains controversial^1–3^. To gain insight into CD4-CD8 lineage commitment, we used pseudotime to characterize expression changes in TCR signaling targets, key transcription factors and coreceptors involved in this process. As expected, we observed that RNA expression often preceded the corresponding change in protein expression over pseudotime, as seen for *Cd69*/CD69 (Extended Data Fig. 3a), likely due to the time needed for protein translation, transport and degradation. As expected, the TCR response (exemplified by expression of TCR targets *Cd69* and *Egr1*) became significantly higher in CD4-fated cells early in pseudotime (by bin 4–5, within DP2) (Fig. 3a,b). This pattern reversed at later pseudotimes, with higher TCR responses in CD8-fated cells at pseudotime bin 9–10 (within DP3) (Fig. 3a,b). This suggested two distinct waves of TCR signaling during positive selection: a broader initial wave in CD4-fated cells and a later wave, specific for CD8-fated cells (Fig. 3a and Extended Data Fig. 3b).

We next used pseudotime to identify the divergence of transcription factors between the lineages and relate these expression patterns to the timing of TCR signaling. We focused on the lineage-defining transcription factors *Runx3* (CD8^+^ T cell lineage), *Zbtb7b* (CD4^+^ T cell lineage) and *Gata3* (upstream activator of *Zbtb7b* that is more highly expressed in the CD4^+^ T cell lineage)^10,27^. The differential upregulation of *Gata3* in CD4-fated cells coincided with differential expression of the first TCR response wave in pseudotime bin 4–5 (within DP2), followed by differential upregulation of *Zbtb7b* in bin 7–8 (within CD4^+^CD8^lo^) (Fig. 3c,d). Differential upregulation of *Runx3* in CD8-fated cells occurred between pseudotimes 8 and 10, overlapping with DP3 and the second rise in TCR signaling (Fig. 3d). Intracellular flow cytometry in wild-type thymocytes supported the observed timing in differential expression of these transcription factors (Extended Data Fig. 3c). IL-7 and other STAT5 activating cytokines were reported to promote *Runx3* upregulation and the CD8^+^ T cell fate^3^, but we did not observe a lineage-specific increase in STAT5 target gene expression correlating with *Runx3* upregulation (Extended Data Fig. 3d).

**Extended Data Fig. 4:**
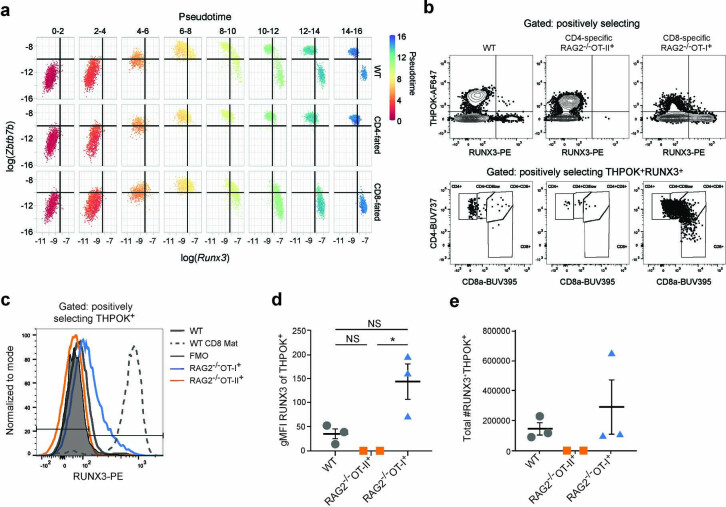
CITE-seq and fluorescence-based flow cytometry of key transcription factors. **a**, In silico flow cytometry plots of log(totalVI denoised expression) of *Runx3* and *Zbtb7b* from positively selected thymocytes separated by pseudotime. **b**, Dual expression of THPOK and RUNX3 in positively selecting CD8-fated thymocytes. Top row shows representative flow cytometry contour plots of gated positively selecting thymocytes (CD5^+^, CD4^+^ or CD8^+^, CD24^lo/int^) displaying RUNX3 vs THPOK protein expression from 6-8-week-old, adult wild-type, CD4-fated (Rag2^-/-^/OT-II^+^) and CD8-fated (Rag2^-/-^/OT-I^+^) mice. Positive staining and gates were determined using fluorescence minus one (FMO) controls. Bottom row shows representative FACS dot plots displaying CD8a vs CD4 expression in positively selecting RUNX3^+^THPOK^+^ thymocytes. **c**, Representative histogram overlays displaying RUNX3 expression in THPOK^+^ positively selecting thymocytes from Rag2^-/-^/OT-II^+^ (orange), Rag2^-/-^/OT-I^+^ (blue) and wild-type (WT, gray) mice. RUNX3 expression in CD8 Mat cells (CD8^+^CD4^−^TCR^+^) from WT mice (gray, dashed line) is included for comparison. FMO is displayed as thin line, filled histogram (black). **d**, Compiled data showing geometric mean fluorescent (gMFI) intensity of RUNX3 on THPOK^+^ positively selecting thymocytes. gMFI for each sample was calculated by subtracting the gMFI of the FMO. **e**, Total number of positively selecting RUNX3^+^THPOK^+^ thymocytes in each mouse. Data is compiled from two independent experiments. Error bars indicate mean ± SEM. In **d-e**, each symbol represents 1 mouse. For WT mice (*n* = 3), Rag2^-/-^/OT-II^+^ (*n* = 2), and Rag2^-/-^/OT-I^+^ (*n* = 3). NS, not significant.

We observed that *Gata3* induction was followed by a rise in *Zbtb7b* in both lineages, although their expression was lower and more transient in CD8-fated cells compared to CD4-fated cells (Fig. 3c,d and Extended Data Fig. 3b). This suggested that both lineages initially ‘audition’ for the CD4^+^ T cell fate, although MHCI-specific cells do so unsuccessfully. A large rise in *Runx3* expression, which occurred only in CD8-fated cells, overlapped with the decrease in *Zbtb7b* at pseudotime 7–11 (CD4^+^CD8^lo^ and DP3 stages) (Fig. 3c,d), implying that both transcription factors may be transiently co-expressed in CD8-fated cells, in spite of their ability to repress each other’s expression and their reported mutually exclusive expression at later stages^10,28^. In silico flow cytometry of *Zbtb7b* and *Runx3* showed that both CD4- and CD8-fated thymocytes initially upregulated *Zbtb7b*, whereas CD8-fated thymocytes subsequently downregulated *Zbtb7b*, simultaneous with *Runx3* upregulation (Fig. 3e and Extended Data Fig. 4a). To test the co-expression of THPOK and RUNX3 in CD8-fated cells, we performed intracellular flow cytometry in OT-I mice, which have a prominent CD4^+^CD8^lo^ population^23^. Wild-type and OT-II mice were included for comparison. We observed a small population of cells co-expressing THPOK and RUNX3 in the positively selecting OT-I thymocytes and wild-type thymocytes, but not in the OT-II thymocytes (Extended Data Fig. 4b). The expression of RUNX3 in THPOK^+^ OT-I thymocytes was substantially lower than in mature CD8^+^ thymocytes, but was significantly above background, as determined by fluorescence minus one controls and staining of THPOK^+^ OT-II thymocytes (Extended Data Fig. 4c–e). These data suggest that OT-I thymocytes contain a population that recently failed the CD4 audition and were transitioning towards CD8^+^ T cell lineage specification.

**Extended Data Fig. 5:**
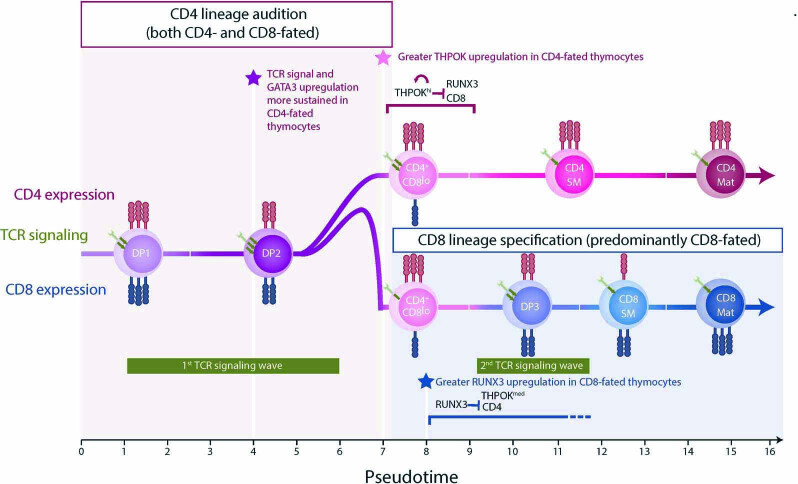
Sequential selection model of thymocyte development. A sequential selection model for CD4^+^ versus CD8^+^ T cell lineage commitment. Key events during positive selection inferred from CITE-seq data are displayed from left to right in their order of occurrence based on pseudotime. CD4-fated and CD8-fated indicate MHCII- and MHCI-specific thymocytes, respectively. Colored circles indicate the order of appearance of key thymocyte stages as defined by cell surface markers. Shaded red area indicates the time window during which both CD4- and CD8-fated thymocytes audition for the CD4^+^ T cell fate, corresponding to upregulation of GATA3 followed by THPOK. Shaded blue area indicates the later time window during which those thymocytes that failed the CD4 audition (mostly CD8-fated) receive CD8^+^ T cell lineage reinforcement and survival signals. Green horizontal bars indicate two distinct temporal waves of TCR signaling: a first wave that is stronger and more sustained in CD4- compared to CD8-fated thymocytes, and a second later wave that occurs only in CD8-fated thymocytes during the CD8^+^ T cell lineage specification phase. Stars indicate the key time points of lineage divergence, including the earliest detection of greater TCR signals and GATA3 upregulation in CD4-fated thymocytes (purple star), followed by preferential THPOK induction and CD8 repression in CD4-fated thymocytes (pink star), and finally preferential RUNX3 induction and CD4 repression in CD8-fated thymocytes (blue star). Red bracket indicates the time window during which MHCII-specific thymocytes commit to the CD4^+^ T cell lineage by fully upregulating THPOK, leading to activation of a THPOK autoregulation loop^44^ and full repression of CD8. Blue bracket indicates the time window during which CD8-fated thymocytes turn on RUNX3, leading to repression of THPOK and CD4.

The pattern of coreceptor expression and its impact on TCR signaling is a key factor in CD4-CD8 lineage commitment^1–4^. CD4 and CD8 both exhibited an initial dip in expression (the ‘double dull’ stage^24^), followed by a rise in CD4 and a continued decrease in CD8 expression (Fig. 3f). In CD8-fated cells, CD8 expression recovered as CD4 expression decreased, resulting in the DP3 stage as the cells progressed towards CD8 SP (Fig. 3f). *Cd8a* became significantly differentially expressed between lineages at pseudotime 6, which corresponded to the rise in *Zbtb7b*, and *Cd4* became differentially expressed at pseudotime 9, which corresponded to the preferential expression of *Runx3* in the CD8^+^ T cell lineage (Fig. 3g). In CD4-fated cells, CD4 expression remained relatively high and was not correlated with expression of *Cd69* and *Egr1* (Fig. 3a,f). The gradual decline in TCR signal after pseudotime 3 was likely due to negative feedback, including induction of the ERK signaling inhibitors *Dusp2/5* (Extended Data Fig. 3b)^29,30^. In contrast, in CD8-fated cells, the faster decline in TCR signaling during the first wave coincided with declining CD8 expression (Fig. 3a,f), as predicted by the kinetic signaling model^3^. Moreover, the second rise in TCR signaling (pseudotimes 8–10) correlated with the rise in CD8 expression (Fig. 3a,f). Thus, the role of CD8 in facilitating MHCI recognition, together with other factors that increase thymocyte sensitivity to TCR signals at later developmental stages^7,9,31^ could explain the second TCR signaling wave. Together, these analyses indicated the existence of an initial CD4^+^ T cell lineage auditioning phase for both MHCI- and MHCII-specific thymocytes and were consistent with a role for TCR signaling in late CD8^+^ T cell lineage specification^5–8^ (Extended Data Fig. 5).

### Differential expression implicates lineage drivers

**Fig. 4:**
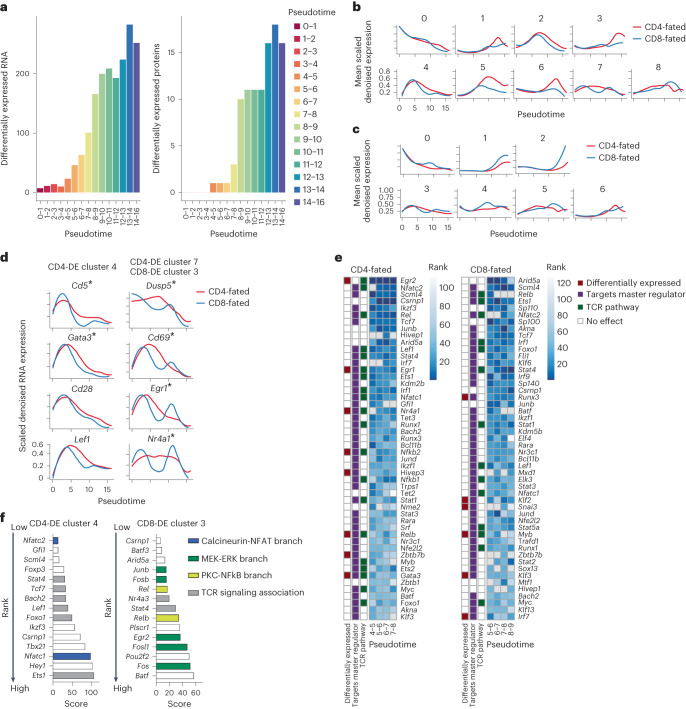
Gene expression differences between CD4-fated and CD8-fated cells implicate putative drivers of lineage commitment. **a**, Number of differentially expressed features between CD4-fated (MHCI^−/−^, OT-II and AND) and CD8-fated (MHCII^−/−^, OT-I and F5) cells across pseudotime. **b**, Genes (RNA) upregulated in CD4-fated versus CD8-fated cells scaled per gene and clustered by the Leiden algorithm according to expression in CD4-fated cells. Expression over pseudotime per cluster is displayed as the mean of scaled totalVI denoised expression per gene for genes in a cluster, smoothed by loess curves. **c**, As in **b**, but for genes upregulated in CD8-fated cells, clustered according to expression in CD8-fated cells. **d**, Expression over pseudotime of selected TCR target genes that are differentially expressed between the two lineages and putative targets of *Nfatc2*, according to ChEA3. Asterisk indicates genes differentially expressed during the time windows used for ranking in **e**. totalVI denoised expression values are scaled per gene and smoothed by loess curves. **e**, Transcription factor enrichment analysis by ChEA3 for CD4-lineage-specific differentially expressed genes (left). Transcription factors are ranked by mean enrichment in the three pseudotime bins before *Zbtb7b* differential expression (pseudotime 4–7; pseudotime 7–8 is for visualization only). Gray indicates genes detected in less than 5% of cells in the relevant population. ‘Differentially expressed’ indicates significant upregulation in at least one of the relevant time bins. ‘Targets master regulator’ indicates a transcription factor that targets either *Gata3*, *Runx3*, or *Zbtb7b* in ChEA3 databases. ‘TCR pathway’ indicates membership in NetPath^34^ TCR Signaling Pathway, genes transcriptionally upregulated by TCR signaling, or genes with literature support for TCR pathway membership. Right column is the same as the left but for CD8 lineage-specific differentially expressed genes, with ranking by mean enrichment in the three pseudotime bins before *Runx3* differential expression (pseudotime 5–8; pseudotime 8–9 is for visualization only). **f**, Transcription factor enrichment analysis for TCR target-enriched gene clusters. The top 15 transcription factors enriched in the gene sets are shown. Colors represent transcription factors activated by the respective branch of TCR signaling. Gray indicates additional transcription factors associated with TCR signaling based on Netpath^34^. In **e** and **f**, lower ranks and lower scores are better (meaning more enrichment).

**Extended Data Fig. 6:**
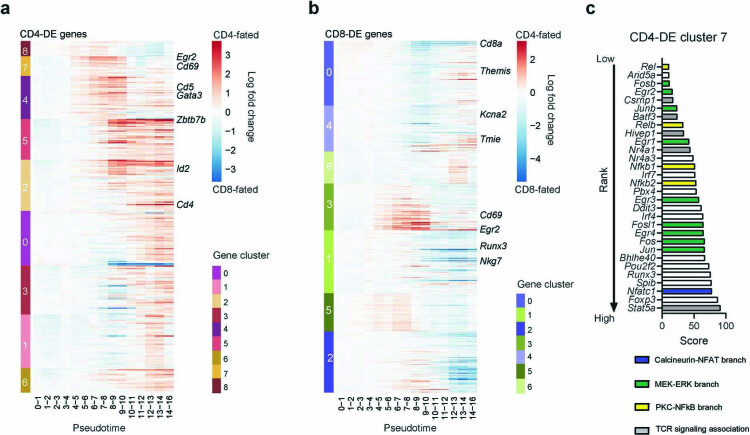
Differential expression and transcription factor enrichment distinguish CD4^+^ and CD8^+^ T cell lineages. **a**, totalVI median log fold change over pseudotime of genes upregulated in CD4-fated cells relative to CD8-fated cells. Genes are grouped by clusters shown in Fig. 4b. Clusters are ordered by their average highest magnitude fold change. **b**, totalVI median log fold change over pseudotime of genes downregulated in CD4-fated cells relative to CD8-fated cells (that is, upregulated in CD8-fated cells). Genes are grouped by clusters shown in Fig. 4c. Clusters are ordered by their average highest magnitude fold change. **c**, Transcription factor (TF) enrichment analysis for TCR target-enriched gene cluster CD4-DE cluster 7. The top 30 TFs enriched in the gene set are shown. The full ChEA3 enrichment analysis is in Supplementary Data 11-12. Colors indicate TFs activated by the respective branch of TCR signaling. Gray indicates additional TFs associated with TCR signaling based on Netpath^34^. Lower ranks and lower scores are better (meaning more enrichment).

To systematically investigate CD4-CD8 lineage divergence, we performed totalVI differential expression tests between lineage-restricted thymocytes within equivalent units of pseudotime. We found no substantial differences in RNA or protein expression between the lineages at the early DP stages, but differential expression accumulated throughout maturation (Fig. 4a, Supplementary Data 7 and Supplementary Information). This analysis resulted in a set of 302 genes with significantly higher expression in CD4-fated thymocytes (hereafter CD4-DE), 397 genes with significantly higher expression in CD8-fated thymocytes (CD8-DE) and 92 genes with higher expression in each lineage in at least one pseudotime unit that were included in both sets (Extended Data Fig. 6a,b). The genes in each set were clustered by their expression in cells of the corresponding lineage (Fig. 4b,c and Supplementary Data 8 and 9). Inspection of mean gene expression of each cluster over pseudotime reflected temporal variations in expression (Fig. 4b–d). For example, CD4-DE cluster 5 and CD8-DE cluster 1 showed a late divergence in expression of transcription factors and genes related to effector functions in their respective lineages (*Zbtb7b* and *Cd40lg* in CD4-fated cells, and *Runx3* and *Nkg7* in CD8-fated cells). Three clusters (CD4-DE clusters 4 and 7 and CD8-DE cluster 3) were significantly enriched for TCR target genes (hypergeometric test, Benjamini–Hochberg -adjusted *P* < 0.05). CD4-DE cluster 7 and CD8-DE cluster 3 contained overlapping TCR target genes, including *Cd69* and *Egr1*, and showed an early expression peak that was more sustained in CD4-fated relative to CD8-fated cells, and a second peak, specifically in CD8-fated cells (Fig. 4b–d). TCR target genes in CD4-DE cluster 4, including *Cd5* and *Gata3*, displayed a similarly early, single peak, which was more sustained for CD4-fated cells (Fig. 4b,d). CD8-DE clusters 0 and 4 exhibited increased expression in CD8-fated cells just before the second rise in TCR signaling and contained genes implicated in modulating TCR sensitivity (Fig. 4c). These included *Cd8a*, which is required for MHCI recognition and *Themis*, which modulates TCR signal strength during positive selection^32^ in CD8-DE cluster 0, and the ion channel component genes *Kcna2* and *Tmie*, which contribute to enhancing TCR sensitivity in thymocytes with low self-reactivity^31^, in CD8-DE cluster 4.

To identify which transcription factors may influence lineage commitment, we focused on pseudotimes 4–7, just after differential gene expression was first detected and before *Zbtb7b* induction, for the CD4^+^ T cell lineage, and pseudotimes 5–8, just before *Runx3* induction, for the CD8^+^ T cell lineage. We performed transcription factor enrichment analysis with ChEA3, which identifies the transcription factors most likely to explain the expression of a set of target genes^33^. We used differentially expressed genes between lineages in each unit of pseudotime as the target gene sets (Fig. 4e and Supplementary Data 10 and 11). For each transcription factor, we also considered known associations with TCR signaling^34^, evidence that it regulates *Gata3*, *Zbtb7b* or *Runx*^33^, and whether the transcription factor itself was differentially expressed at the relevant pseudotime. In CD4-lineage cells, several top-ranked transcription factors by ChEA3 were associated with TCR signaling pathways (*Egr2*, *Nfatc2*, *Egr1*, *Nfatc1* and *Rel*) (Fig. 4e). The top two in CD4-fated cells were *Egr2* and *Nfatc2*, which lie downstream of the extracellular signal-regulated kinase branch (hereafter MEK-ERK) and calcineurin-NFAT branch, respectively^35–38^, two of the three main branches of the TCR signal transduction pathway.

To explore how TCR signaling associated with divergent transcriptional regulation between the two lineages, we examined genes in CD4-DE clusters 4 and 7 and CD8-DE cluster 3, which all showed an early peak that corresponded to the CD4 audition phase in pseudotime. CD4-DE cluster 4 contained *Gata3*, a target of the TCR-associated transcription factor NFAT^34,39,40^, exhibited more transient expression and lacked a prominent second peak in CD8-fated cells (Fig. 4b), implying these genes were regulated by a branch of the TCR signaling pathway that was selectively active early during the CD4 audition. ChEA3 analysis of the genes in CD4-DE cluster 4 showed enrichment for NFAT family member *Nfatc2*, with *Gata3*, *Cd5*, *Id3*, *Cd28* and *Lef1* contributing to the enrichment score (Fig. 4f and Supplementary Data 12). By contrast, CD4-DE cluster 7 and CD8-DE cluster 3 showed enrichment for the AP-1 transcription factors *Fosb* and *Junb*, NF-κB family members *Rel* and *Nfkb1/2*, and MEK-ERK target *Egr1* (Fig. 4f, Extended Data Fig. 6c and Supplementary Data 13). This suggested that all three branches of the TCR signaling pathway participated during the CD4 audition, whereas only MEK-ERK and PKC-NF-κB were active in the later CD8 specification. Thus, ChEA3 analyses implicated NFAT in driving early TCR-induced transcriptional differences between lineages (Fig. 4e).

### Calcineurin-NFAT promotes CD4^+^ T cell lineage via GATA3

**Fig. 5:**
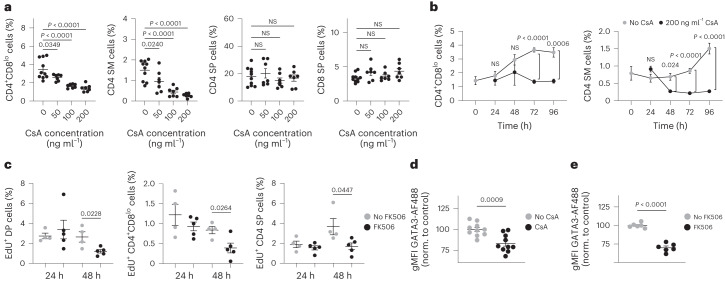
Calcineurin blockade impairs new CD4 SP development and GATA3 induction. **a**, Frequency (% of live cells) of CD4^+^CD8^lo^, CD4 SM, CD4 SP or CD8 SP in thymic slices from postnatal (day 1) wild-type mice cultured with in 50, 100 or 200 ng ml^−1^ cyclosporin A (CsA) for 96 h. Data were compiled from three independent experiments and analyzed using an ordinary one-way ANOVA. No CsA, *n* = 10; 50 ng ml^−1^, 100 ng ml^−1^ and 200 ng ml^−1^, *n* = 7 each. Gating strategy, Extended Data Fig. 7a. **b**, Frequency (% of live cells) of CD4^+^CD8^lo^ and CD4 SM cells after 0, 24, 48, 72 and 96 h culture in medium alone or with 200 ng ml^−1^ CsA as in **a**. Data were compiled from nine independent experiments. No CsA: 0 h, *n* = 6; 24 h, *n* = 9; 48 h, *n* = 10; 72 h, *n* = 22; 96 h, *n* = 10. 200 ng ml^−1^ CsA: 24 h, *n* = 6; 48 h, *n* = 7; 72 h, *n* = 14; 96 h, *n* = 7. Data were analyzed using an ordinary two-way ANOVA with multiple comparisons. **c**, Frequency (% of live cells) of EdU^+^ DP, EdU^+^ CD4^+^CD8^lo^, and EdU^+^ CD4 SP thymocyte populations from AND mice (i.p.) injected with EdU, rested for 16 h, and then treated with 1 (24 h) or 2 (48 h) 5 μg doses of FK506 (i.p.) every 24 h. Each dot represents one mouse. Data were pooled from three independent experiments. FK506 mice, *n* = 5; control, *n* = 4. **d**,**e**, Geometric mean fluorescent intensity (gMFI) of GATA3 detected by intracellular flow cytometry staining in CD69^+^ DP thymocytes from wild-type neonatal thymic slice cultures after 48-h treatment with 200 ng ml^−1^ CsA (**d**) or 72-h treatment with 6.3 ng ml^−1^ FK506 (**e**). Data were compiled from three (**d**) or two (**e**) independent experiments. For **d**, no CsA, *n* =  10 ; 200 ng ml^−1^CsA, *n* = 10. For**e**, no FK506, *n* = 6; 6.3 ng ml^−1^ FK506, *n* = 6. Each symbol represents a thymic slice. Data were analyzed using an unpaired, two-sided *t*-test. norm., normalized; NS, not significant. Error bars indicate mean ± s.e.m.

**Extended Data Fig. 7:**
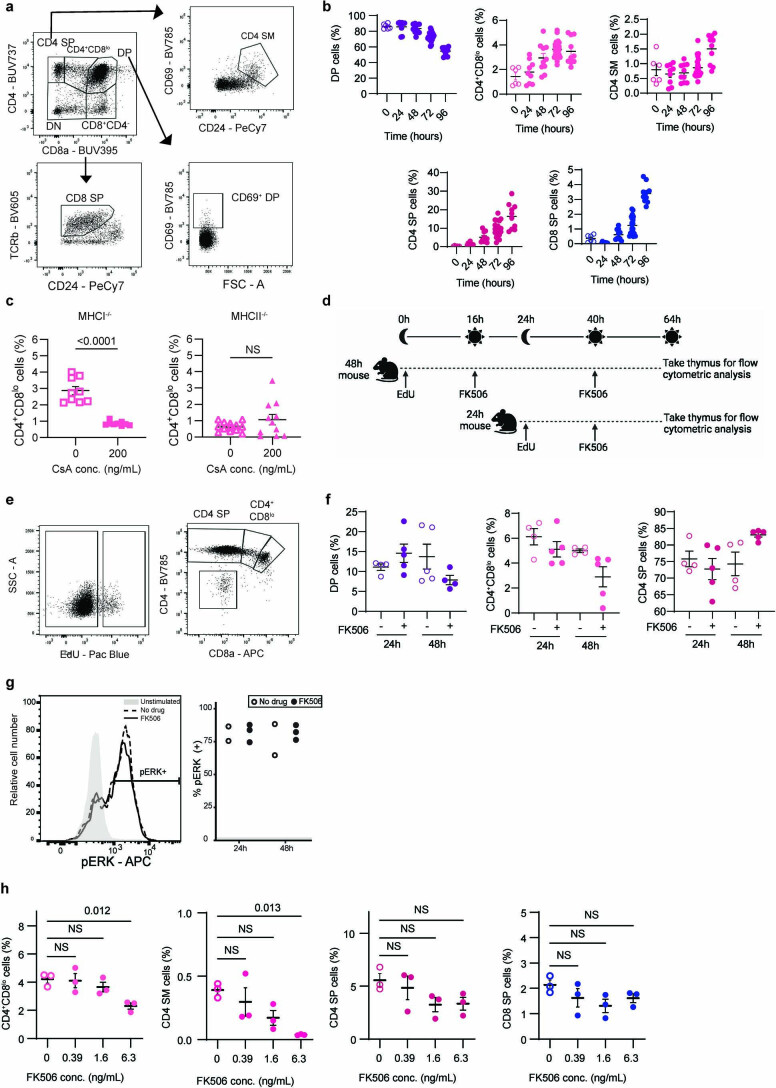
Inhibition of calcineurin blocks new CD4 SP development and GATA3 induction. **a**, Flow cytometry gating strategy for neonatal thymic slice samples. **b**, Time course of thymocyte development in neonatal slice cultures. Frequency (% of live cells) of the indicated populations after 0 (*n* = 6), 24 (*n* = 9), 48 (*n* = 10), 72 (*n* = 22) and 96 (*n* = 10) hours of culture. Error bars indicate mean ± SEM. Data were compiled from 9 independent experiments with wild-type (WT) slices. **c**, Frequency (% of live cells) of CD4^+^CD8^lo^ cells in slices from MHCI^-/-^ (squares) or MHCII^-/-^ (triangles) mice following culture in medium alone (No CsA) or with 200 ng ml^-1^ CsA for 96 hours. Error bars indicate mean ± SEM. Data were compiled from 2 independent experiments with MHCI^-/-^ thymic slices: no CsA (*n* = 9), 200 ng ml^-1^ CsA (*n* = 9), and 5 independent experiments with MHCII^-/-^ slices: no CsA (*n* = 13), 200 ng ml^-1^ CsA (*n* = 11). **d**, Schematic of in vivo EdU labeling and calcineurin blockade. AND mice were injected with EdU to label proliferating thymocytes undergoing TCRβ selection. Starting at 16 hours mice were treated with the calcineurin inhibitor FK506 daily for 24 or 48 hours. Thymocytes were analyzed by flow cytometry. **e**, Gating strategy for in vivo EdU-FK506 experiment. **f**, Frequency (% of live cells) of the indicated thymocyte populations with and without FK506. Each dot represents an individual mouse (*n* = 5 for FK506-treated, *n* = 4 for non-treated), and data are pooled from 3 independent experiments. Error bars indicate mean ± SEM. **g**, Thymocytes from FK506-treated or control AND mice were stimulated via TCR crosslinking and analyzed by intracellular pERK staining and flow cytometry. Left panel shows a representative histogram of pERK induction in gated CD4^+^CD8^+^ thymocytes upon TCR crosslinking. Unstimulated samples (no crosslinking) are shown in gray. Right panel shows quantification of pERK induction (% of DP) with and without FK506 treatment. Data are compiled from 2 independent experiments, and each dot corresponds to a sample (+ FK506 *n* = 3, control *n* = 2). **h**, Frequency of the indicated populations after culture in the presence of the indicated concentration of FK506 for 72 hours. Data is from one experiment (*n* = 3), representative of 2 independent experiments. Each dot represents a thymic slice. Error bars indicate mean ± SEM. Data was analyzed using an ordinary one-way ANOVA. NS indicates not significant.

Genetic disruption of the calcineurin B1 regulatory subunit in thymocytes, or 10-day in vivo treatment with calcineurin inhibitors leads to a developmental defect in DP thymocytes that obscures a possible role of calcineurin downstream of TCR signals during positive selection^41^. Because mature SPs first appear shortly after birth in mice, we used ex vivo culture of thymic tissue slices from postnatal day 1 mice to inhibit TCR signaling during a new wave of CD4 and CD8 SP development. Thymic slices cultured for 0 or 24 h contained mostly DP thymocytes, whereas frequencies of CD4 SP, CD8 SP, CD4^+^CD8^lo^ and CD4 SM increased between 48 and 96 h (Extended Data Fig. 7a,b). As expected, CD8^+^ T cell development was slightly delayed compared to that of CD4^+^ T cells^21,42^ (Extended Data Fig. 7b). Treatment of wild-type cultures with 200 ng ml^−1^ or less of the calcineurin inhibitor cyclosporin A (CsA) for 96 h did not impact the relative size of most thymocyte populations while leading to a selective and dose-dependent reduction in CD4^+^CD8^lo^ and CD4 SM thymocytes (Fig. 5a). A similar reduction in CD4^+^CD8^lo^ thymocytes was observed in CsA-treated MHCI^−/−^ cultures, whereas neonatal slice cultures from MHCII^−/−^ mice had a reduced CD4^+^CD8^lo^ population that was not impacted by CsA (Extended Data Fig. 7c). Time course analyses in wild-type cultures showed that the reduction in CD4^+^CD8^lo^ and CD4 SM cells became significant after 72 and 48 h of culture, respectively (Fig. 5b).

To investigate why CsA reduced CD4^+^CD8^lo^ and CD4 SM, without impacting the overall number of CD4 SP, we used EdU to label a cohort of proliferating DP thymocytes that just completed TCRβ selection, and we followed those cells for 2 days in the presence or absence of calcineurin blockade (Extended Data Fig. 7d,e). We used the calcineurin inhibitor FK506, which blocks positive selection without the loss of DP thymocytes observed with CsA^43^. Adult AND mice were injected i.p. with 1 dose of EdU followed by i.p. injection with FK506 every 24 h starting 16 h post-EdU administration. In control mice that were injected with EdU but not FK506, the percentage of EdU^+^ CD4 SP increased from ~2% to 4% between 24 and 48 h (Fig. 5c), reflecting the conversion of labeled thymocytes from DP to CD4 SP during the time course. FK506 treatment had no significant impact on the overall percentage of DP or CD4 SP thymocytes (Extended Data Fig. 7f), and a 24-h treatment had no significant impact on the number of EdU^+^ DP or CD4 SP compared to samples without FK506 (Fig. 5c). However, a 48-h treatment with FK506 led to a significant reduction in the EdU^+^ CD4 SP, CD4^+^CD8^lo^ and DP thymocytes (Fig. 5c), suggesting a reduction of newly developed CD4 SP. To confirm that the reduction in CD4 SP development was not an indirect consequence of impaired ERK activation^41^, we stimulated thymocytes from FK506-treated mice by TCR crosslinking for 2 min, followed by flow cytometry to detect phosphorylated ERK (p-ERK). Strong p-ERK induction in DP thymocytes was detected in 24- and 48-h FK506-treated mice, similar to untreated controls (Extended Data Fig. 7g). Together these data indicated that blockade of calcineurin activation downstream of TCR during positive selection prevented new CD4 SP development but did not interfere with already selected CD4 SP thymocytes.

To test whether CsA treatment prevented CD4 development by interfering with GATA3 induction, we quantified GATA3 expression in neonatal slice cultures treated with CsA for 48 h. We observed a significant reduction in GATA3 expression in CD69^+^ DP from CsA-treated cultures compared to untreated cultures (Fig. 5d). In addition, neonatal slice cultures treated with moderate concentrations (0.4–6 ng ml^−1^) of FK506 exhibited a loss of CD4^+^CD8^lo^ and CD4 SM thymocytes without a significant loss of mature CD4 or CD8 SP (Extended Data Fig. 7h), similar to CsA. FK506 treatment also led to a significant reduction in GATA3 protein expression in CD69^+^ DP (Fig. 5e). Together, these data implicated the calcineurin-NFAT-GATA3 axis as a link between TCR signals downstream of MHCII recognition and commitment to the CD4^+^ T cell lineage.

### Calcineurin blockade selectively impacts the CD4 audition

**Fig. 6:**
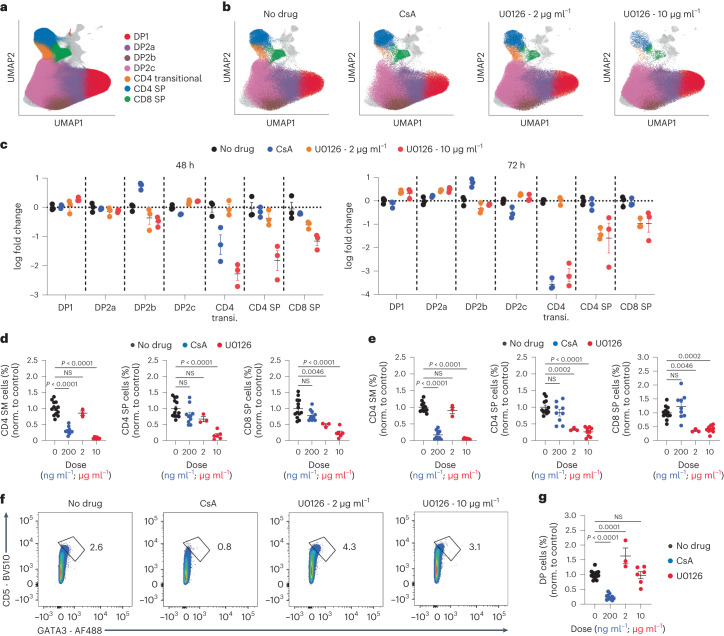
Calcineurin inhibition selectively impacts the CD4 audition. **a**, UMAP plots of individual thymocytes from thymic slices of postnatal (day 1) wild-type mice cultured with no drug or CsA (200 ng ml^−1^) or U0126 (2 μg ml^−1^ or 10 μg ml^−1^) for 48 or 72 h. DP and SP populations are highlighted. **b**, UMAP plots for the 48-h time point separated into no drug, 200 ng ml^−1^ CsA, 2 μg ml^−1^ or 10 μg ml^−1^ U0126 conditions as in **a**. **c**, Scatter plots showing the log fold change in cell-type proportions relative to no-drug control for DP1, DP2a, DP2b, DP2c, CD4 transitional, CD4 SP and CD8 SP cell clusters for no drug, 200 ng ml^−1^ CsA, 2 μg ml^−1^ or 10 μg ml^−1^ U0126 conditions. Each dot is a separate thymic slice, and data are normalized to the mean of the no-drug condition. Error bars indicate mean ± s.e.m. **d**,**e**, Frequency (% of live cells) in CD4 SM, CD4 SP and CD8 SP populations normalized to untreated control at 48 h (**d**) or 72 h (**e**) of culture as in **a**, assessed by manual gating (Extended Data Fig. 7a). Error bars indicate mean ± standard error of the mean. **f**,**g**, Representative flow cytometry plots (**f**) and compiled data (**g**) showing the expression of CD5 and GATA3 in gated DP thymocytes from thymic slices cultured for 48 h with no drug, 200 ng ml^−1^ CsA, 2 μg ml^−1^ or 10 μg ml^−1^ U0126 as in **a**. **a**–**c**, Data are from one representative experiment out of two. **d**,**e**,**g**, Each dot represents a thymic slice; data are compiled from three independent experiments for CsA (200 ng ml^−1^), *n* = 10; one experiment for U0126 low (2 μg ml^−1^), *n* = 3; two independent experiments for U0126 high (10 μg ml^−1^), *n* = 6; and four independent experiments for no drug, *n* = 13.

**Extended Data Fig. 8:**
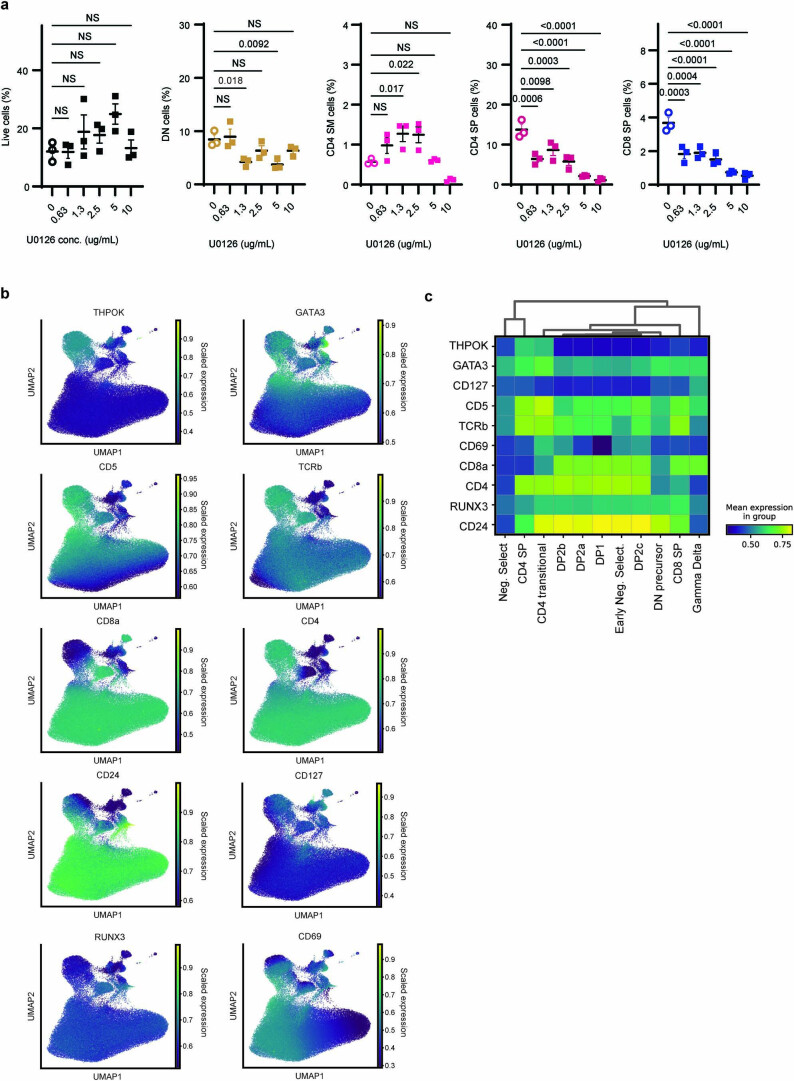
Multidimensional computation gating of neonatal slice cultures with calcineurin or MEK blockade. Thymic tissue slices were prepared from postnatal (day 1) mice and cultured with either calcineurin inhibitor CsA or MEK inhibitor U0126. **a**, U0126-treated thymic slices were incubated in concentrations ranging from 10 μg ml^-1^ to 0.63 μg ml^-1^. Plots show frequency (%) out of live cells. Each dot represents a thymic slice (*n* = 3). Plots are representative of 3 independent experiments. **b**, UMAP plots of multidimensional flow cytometry data colored by scaled expression of each flow cytometry marker. Data are pooled samples from the representative experiment shown in Fig. 6a–c and Extended Data Fig. 9. **c**, Heatmap of relative protein expression of markers in each subset shown in Fig. 6a–c and Extended Data Fig. 9.

**Extended Data Fig. 9:**
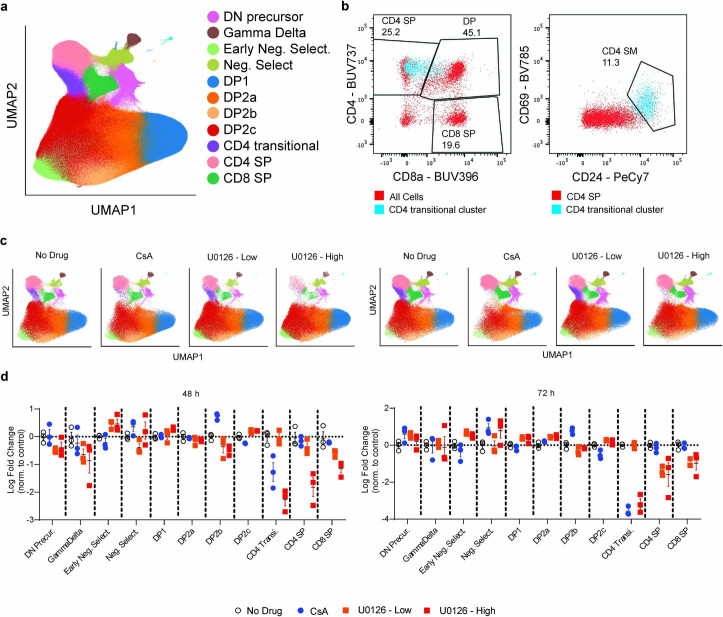
Calcineurin inhibition selectively impacts the CD4 audition. Thymic tissue slices were prepared from postnatal (day 1) mice and cultured with either calcineurin inhibitor CsA (200 ng ml^-1^) or MEK inhibitor U0126 (2 μg ml^-1^ or 10 μg ml^-1^). Thymic slices were collected at 48 or 72 hours, stained with fluorescent antibodies and analyzed by either manual or computational, multidimensional gating. **a**, UMAP based on multidimensional flow cytometry data from all samples. **b**, Cells from the CD4 transitional cluster defined by computational gating (blue) are superimposed on live gated cells (red, left panel) or CD4^+^CD8^-^ gated cells (red, right panel) for comparison with manual gating strategy. A 48-hour, no drug control sample is shown. **c**, UMAP plot by the indicated experimental condition. **d**, Scatter plots showing the log fold change in cell type proportion relative to no drug control for indicated cell clusters for each condition, separated by time (left panel is 48 hours; right panel is 72 hours). Error bars indicate mean ± SEM. Data is from one representative experiment out of two.

To investigate whether the calcineurin-NFAT branch of the TCR signaling pathway had a selective role in the CD4 audition, we compared the impact of calcineurin versus MEK inhibition in neonatal thymic slice cultures. We used relatively low CsA (200 ng ml^−1^) and U0126 (2 or 10 μg ml^−1^) concentrations based on titration experiments (Extended Data Fig. 8a) to avoid off-target effects. We combined flow cytometric analyses of seven cell surface proteins (CD4, CD8α, CD5, TCRβ, CD69, CD24 and CD127) and three lineage-defining transcription factors (GATA3, THPOK and RUNX3) with a computational multidimensional gating approach by unsupervised clustering to define continuous developmental intermediates in an unbiased manner. These analyses identified populations that largely overlapped with the CD4 SP, CD8 SP and DP populations defined by manual gating, as well as a transitional population that largely overlapped with the CD4 SM population and also included some CD4^+^CD8^lo^ cells (Fig. 6a and Extended Data Figs. 8b,c and 9a,b). Smaller populations of αβTCR^−^ and mature, unconventional T cells were also detected (Extended Data Fig. 9a). Wild-type cultures treated with CsA for 48 or 72 h had a significant reduction in the CD4 transitional population, with little impact on other populations, compared to untreated controls (Fig. 6b,c and Extended Data Fig. 9c,d). Cultures treated with U0126 at 10 μg ml^−1^ had a loss of CD4 SP and CD8 SP as well as the CD4 transitional population, whereas those treated with 2 μg ml^−1^ U0126 had normal numbers of transitional CD4 cells and slightly reduced CD4 SP and CD8 SP (Fig. 6b,c and Extended Data Fig. 9c,d). Similar results were obtained with manual gating (Fig. 6d,e) and suggested that calcineurin inhibition impacted a relatively restricted temporal window during positive selection that corresponded to the CD4 audition, whereas MEK inhibition impacted all stages of positive selection, including CD8 specification.

Calcineurin inhibition by CsA also impacted two computationally defined DP clusters (DP2b and DP2c) (Fig. 6c), that differed in their relative expression of CD5 (Extended Data Fig. 8c). Because CD5, along with GATA3, was one of the putative NFAT targets based on transcription factor enrichment analyses (Fig. 4e and Supplementary Data 12), we used manual gating in DP thymocytes to compare the expression of CD5 and GATA3 in neonatal slice cultures. We observed a small population of DP thymocytes with high expression of CD5 and GATA3 in wild-type cultures, which was proportionally decreased by treatment with CsA for 48 h, and increased by treatment with 2 μg ml^−1^ U0126 (Fig. 6f,g). Together, these data indicated that calcineurin-NFAT promoted strong induction of GATA3 and CD5 during the CD4 audition, thus promoting CD4^+^ T cell fate commitment, whereas MEK-ERK signaling provided more general differentiation and survival signals throughout positive selection.

## Discussion

Here, we applied single-cell multiomic analysis to generate a high-resolution timeline of RNA and surface protein expression throughout T cell maturation in the thymus. We identified an initial CD4 auditioning phase in which both CD4-fated and CD8-fated cells undergo a parallel induction of a CD4^+^ T cell differentiation program during a first TCR signaling wave, followed by a second TCR signaling wave that is specific for CD8-fated cells and overlaps with induction of a CD8^+^ T cell differentiation program. Our data confirmed and extended earlier analyses based on more limited sets of markers^15,21,28,42,44^, providing a comprehensive picture of events during CD4^+^ and CD8^+^ T cell development that can serve as a resource for future studies.

We used a high-resolution timeline to dissect the activity of TCR-regulated transcription factors during lineage commitment. Previous studies showed that the MEK-ERK branch of the TCR signaling pathway has a crucial role during positive selection^9,45–47^, the NF-κB branch is not required for positive selection^48^ and the role of the calcineurin-NFAT branch is unclear^41^. Our analyses showed that NFAT activity is likely to account for some of the earliest lineage-specific RNA differences and was most prominent during the CD4 audition, whereas the inferred activity of NF-κB and the MEK-ERK regulated factors AP-1 and EGR1/2 occurred throughout positive selection. Although previous work showed that long-term loss of calcineurin-NFAT activity impairs the ability of DP thymocytes to activate ERK upon TCR triggering^41^, we found that short-term, low-dose exposure to calcineurin inhibitors, conditions that did not impair ERK activation, prevented new development of mature CD4^+^ T cells and decreased the expression of GATA3 in DP thymocytes. These data are consistent with earlier studies implicating NFAT as a positive regulator of GATA3^39,40^, and GATA3 as a major driver of the CD4 fate^49^, which points to a TCR–calcium–calcineurin–NFAT–GATA3 axis in driving CD4^+^ T cell lineage commitment.

The timeline of RNA and protein expression changes presented here provided a useful framework for understanding how TCR specificity for MHCI versus MHCII directs T cell fate. In particular, the notion of successive windows of opportunity for CD4^+^, followed by CD8^+^ T cell fate determination, corresponding with distinct waves of TCR signaling is consistent with a ‘sequential selection’ model for lineage determination^50^. In this model, the CD4 audition serves as an initial selection step to ensure a match between CD4 coreceptor expression and an MHCII-specific TCR, whereas the second TCR signaling wave provides an additional selection step to ensure a match between CD8 expression and an MHCI-specific TCR. During the CD4 audition, thymocytes bearing MHCII-specific TCRs experience a relatively sustained first wave of TCR signaling, allowing them to lock in the CD4 fate. On the other hand, thymocytes bearing MHCI-specific TCRs experience a more transient first signaling wave, due in part to the drop in CD8 coreceptor expression (in line with kinetic signaling^3^), resulting in a failed CD4 audition. After the failed audition, MHCI-specific thymocytes experience a second wave of TCR signaling driving CD8 fate specification. The notion of a second TCR-driven selection stage for CD8-fated cells fits with prior evidence for a prolonged requirement for TCR signaling for CD8^+^ T cell development^5–7,9^ but is at odds with the kinetic signaling model, which invokes a complete loss of TCR signals and an exclusive role for cytokine signals during CD8^+^ T cell lineage specification^3^.

Although the current study focused on T cell lineage commitment, the approach used here has broader utility for studying developmental systems. The simultaneous measurement of RNA and protein not only allowed us to track the differences in relative timing of RNA and protein expression events but also can inform the design of high-dimensional flow cytometry studies for further analyses of developmental intermediates. Future work that integrates spatial measurements with our transcriptomic and proteomic profiles would provide valuable information about how signals from the tissue environments impact cell fate decisions.

## Methods

### Mice

All animal care and procedures were carried out in accordance with guidelines approved by the Institutional Animal Care and Use Committees at the University of California, Berkeley and at BioLegend, Inc. Wild-type (B6) (C57BL/6, 000664), *B2m*^–/–^ (B6.129P2-B2m^tm1Unc^/DcrJ, 002087; referred to as MHCI^−/−^), OT-I (C57BL/6-Tg(TcraTcrb)1100Mjb/J, 003831), and OT-II (B6.Cg-Tg(TcraTcrb)425Cbn/J, 004194) were obtained from The Jackson Laboratory. MHCII^−/−^ (*H2-Ab1*^−/−^, also known as *I-Aβ*^−/−^) mice have been previously described^52^. AND TCRtg *Rag1*^−/−^ mice and F5 TCRtg *Rag1*^−/−^ mice were generated by crossing AND TCRtg (B10.Cg-Tg(TcrAND)53Hed/J, 002761)^53^ and F5 TCRtg (C57BL/6-Tg(CD2-TcraF5,CD2-TcrbF5)1Kio)^54^ mice with *Rag1*^−/−^ mice (Rag1^−/−^B6.129S7-Rag1^tm1Mom^) as previously described^7^).

### CITE-seq experiment

All mice used in CITE-seq experiments were females between 4 and 8 weeks of age. Samples are further described in Supplementary Data 2. Mice were group housed with enrichment and segregated by sex in standard cages on ventilated racks at an ambient temperature of 26 °C and 40% humidity. Mice were kept in a dark/light cycle of 12 h on and 12 h off and given access to food and water ad libitum. For cell preparation, mice were euthanized and thymi were harvested, placed in RPMI with 10% FBS medium on ice, mechanically dissociated with a syringe plunger and passed through a 70 μm strainer to generate a single-cell suspension.

For antibody panel preparation, we prepared a panel containing 111 antibodies (TotalSeq-A mouse antibody panel 1, BioLegend, 900003217), which are enumerated in Supplementary Data 1. Immediately before cell staining, we centrifuged the antibody panel for 10 min at 14,000*g* to remove antibody aggregates. We then performed a buffer exchange on the supernatant using a 50 kDa Amicon spin column (Millipore, UFC505096) following the manufacturer’s protocol to transfer antibodies into RPMI with 10% FBS.

To enrich for positively selecting thymocytes in MHC-deficient and some wild-type samples (Supplementary Data 2), live, single TCRβ^+^CD5^+^ thymocytes were sorted by FACS. We took advantage of the fact that cells were already stained with TotalSeq (oligonucleotide-conjugated) antibodies and therefore designed oligonucleotide-fluorophore conjugates complementary to the TotalSeq barcodes (5′-CACTGAGCTGTGGAA-AlexaFluor488-3′ for CD5; 5′-TCCCATAGGATGGAA-AlexaFluor647-3′ for TCRβ). Before cell staining, the TotalSeq antibody panel was mixed with oligonucleotide-fluorophore conjugates in a 1:1.5 molar ratio. This mixture was incubated for 15 min at room temperature to allow for oligonucleotide hybridization and then transferred to ice. Cells were then stained with the antibody/oligonucleotide-fluorophore mixture according to the TotalSeq protocol. Cells were stained, washed and resuspended in RPMI with 10% FBS to maintain viability. Cells were sorted using a BD FACSAria Fusion (BD Biosciences).

The CITE-seq experiment was performed following the TotalSeq protocol. Cells were stained, washed and resuspended in RPMI with 10% FBS to maintain viability. We followed the 10x Genomics Chromium Single Cell 3′ v3 protocol to prepare RNA and antibody-derived-tag (ADT) libraries^55^.

RNA and ADT libraries were sequenced with either an Illumina NovaSeq S1 or an Illumina NovaSeq S4. Reads were processed with Cell Ranger v.3.1.0 with feature barcoding, where RNA reads were mapped to the mouse mm10–2.1.0 reference (10x Genomics, STAR aligner^56^) and antibody reads were mapped to known barcodes (Supplementary Data 1). No read depth normalization was applied when aggregating samples.

### CITE-seq data preprocessing

Before analysis with totalVI, we performed preliminary quality control and feature selection on the CITE-seq data. Cells with a high percentage of UMIs from mitochondrial genes (>15% of a cell’s total UMI count) were removed. We also removed cells expressing <200 genes, and retained cells with protein library size between 1,000 and 10,000 UMI counts. We removed cells in which fewer than 70 proteins were detected of the 111 measured in the panel. An initial gene filter removed genes expressed in fewer than four cells. The top 5,000 highly variable genes (HVGs) were selected by the Seurat v3 method^57^ as implemented by scVI^58^. In addition to HVGs, we also selected genes encoding proteins in the measured antibody panel and a manually selected set of genes of interest. After all filtering, the CITE-seq dataset contained a total of 72,042 cells, 5,125 genes and 111 proteins.

### totalVI analysis of all CITE-seq data

We ran totalVI on CITE-seq data after filtering (described above), using a 20-dimensional latent space, a learning rate of 0.004, and early stopping with default parameters. Each 10x lane was treated as a batch. When generating denoised gene and protein values, we applied the *transform_batch* parameter^17^ to view all denoised values in the context of wild-type samples.

To conduct cell annotation, we stratified cells of the thymus into cell types and states based on the totalVI latent space, taking advantage of both RNA and protein information. We first clustered cells in the totalVI latent space with the Scanpy^59^ implementation of the Leiden algorithm^60^ at resolution 0.6, resulting in 18 clusters. We repeated this approach to subcluster cells. We used Vision^61^ with default parameters for data exploration. Subclusters were manually annotated based on curated lists of cell-type markers^17,18^, resulting in 20 annotated clusters (excluding one cluster annotated as doublets). We visualized the totalVI latent space in two dimensions using the Scanpy^59^ implementation of the UMAP algorithm^51^.

In addition to thymocyte populations previously described, we identified two distinct thymocyte clusters undergoing negative selection, based on expression of *Bcl2l11* (BIM), *Nr4a1* (NUR77) and *Ik2f2* (HELIOS)^62^ (Extended Data Fig. 1b). The first cluster (Neg. Sel. (1)) emerged from DP (Sig.) adjacent to a cluster of dying cells, and possessed markers of an early wave of negative selection^62^ like upregulated *Pdcd1* (PD-1) and downregulated *Cd4*/CD4 and *Cd8*/CD8 (Fig. 1d and Extended Data Fig. 1b,c). The second (Neg. Sel. (2)) emerged from immature CD4^+^ T cells and possessed markers of a late wave of negative selection^62^ like upregulated *Tnfrsf18* (CD357/GITR) and *Tnfrsf4* (CD134/OX40) (Extended Data Fig. 1b,c). *Foxp3*^+^ regulatory T cells clustered near mature conventional CD4^+^ T cells and Neg. Sel. (2) (Fig. 1a). We also detected γδ T cells, NKT cells, B cells, myeloid cells, erythrocytes, a thymocyte population with high expression of interferon response genes^63^ and a population of mature T cells that had returned to cycling following the cell cycle pause during thymocyte development (Fig. 1a).

We conducted a one-vs-all differential expression test between all annotated cell types, excluding clusters annotated as doublets or dying cells. We identified cell-type markers by filtering for significance (log(Bayes factor) > 2.0 for genes, log(Bayes factor) >1.0 for proteins), effect size (median log fold change (LFC) > 0.2 for both genes and proteins), and the proportion of expressing cells (detected expression in >10% of the relevant population for genes), and sorting by the median LFC. For marker visualization, we selected the top four (if existing) differentially expressed genes and proteins per cell type, arranged by the cell type in which the LFC was highest.

### totalVI analysis of positive selection subset of CITE-seq data

To further analyze thymocyte populations with a focus on positively selected cells, we selected the following annotated clusters: Signaled DP, Immature CD4, Immature CD8, Mature CD4, Mature CD8, Interferon signature cells^63^, Negative selection (wave 2), and T_reg_ cells. With an interest in the variation within thymocyte populations (rather than all cells in the thymus), we selected the top 5,000 HVGs in this subset, as well as genes encoding proteins in the measured antibody panel and a manually selected set of genes of interest. This resulted in a CITE-seq dataset containing 35,943 cells, 5,108 genes and 111 proteins. We ran totalVI on this subset dataset and generated denoised values as described above. We performed Leiden clustering and visualized the totalVI latent space in two dimensions using UMAP as described above.

After visualizing the totalVI latent space of the thymocyte subset, we applied additional filters to restrict to cells on the CD4-CD8 developmental trajectory. We used two resolutions of Leiden clustering (0.6 and 1.4) and sub-clustering as described above to identify and remove clusters of negatively selected cells, T_reg_ cells, gamma-delta-like cells, mature cycling cells, and outlier clusters of doublets, interferon signature cells, and CD8-transgenic-specific outlier cells. After filtering, this dataset contained 29,408 cells that were used for downstream analysis. Differential expression testing of positively selecting thymocytes using pseudotime information is described below.

### Pseudotime inference

Slingshot^19^ was selected for pseudotime inference based on its superior performance in a comprehensive benchmarking study^20^. Slingshot pseudotime was derived from the UMAP projection of the totalVI latent space. The starting point was assigned to DP cells, and two endpoints were assigned to mature CD4^+^ and CD8^+^ T cells. Slingshot pseudotime derived from the full 20-dimensional totalVI latent space was highly correlated with that from the 2-dimensional space (Extended Data Fig. 2a), supporting our use of the 2D-derived pseudotime values for ease of visualization and analysis.

Initial lineage assignment of cells was made on the basis of their genotype (CD4^+^ T cell lineage for MHCI^−/−^, AND, and OT-II mice, CD8^+^ T cell lineage for MHCII^−/−^, F5, and OT-I mice, unassigned for wild-type mice). However, small numbers of cells in MHC-deficient and TCRtg mice develop along the alternative lineage (particularly in TCRtgs that are *Rag* sufficient, which might express an endogenous TCR in addition to the transgenic TCR). We therefore added an additional filter of Slingshot lineage assignment weight > 0.5. Cells with a Slingshot lineage assignment weight of < 0.5 along the expected lineage based on genotype were excluded from the remaining pseudotime-based analysis.

### In silico flow cytometry and gating

To perform in silico flow cytometry, totalVI denoised protein counts were log-transformed and visualized in biaxial-style scatter plots. Gates in biaxial plots were determined based on contours of cell density. An approximate alignment of gated populations to pseudotime was generated by identifying thresholds classifying adjacent populations in pseudotime by maximizing the Youden criteria.

### Adult thymocyte population analysis with fluorescence-based flow cytometry

For thymocyte population analysis in adult mice, 6- to 8-week-old eight-week-old wild-type, MHCI^−/−^ or MHCII^−/−^ mice (described above) were used. Thymi were analyzed from eight mice per genotype (four male and four female). All antibodies are described in Supplementary Data 1.

Thymi were mechanically dissociated into a single-cell suspension, depleted of red blood cells using ACK Lysis Buffer (0.15 M NH_4_Cl, 1 mM KHC_3_ and 0.1 mM Na_2_EDTA). Cells were filtered, washed and counted before being stained with a live/dead stain; Zombie NIR Fixable Viability Kit (BioLegend). Samples were blocked with anti-CD16/32 (2.4G2) and stained with surface antibodies against CD4, CD8, TCRβ, CD5, CD69 and CD127 (IL-7Ra) in FACS buffer (1% BSA in PBS) containing Brilliant Stain Buffer Plus (BD Biosciences). Intracellular staining for GATA3, THPOK, and RUNX3 was performed using the eBioscience FOXP3/Transcription Factor Staining Buffer Set (Thermo Fisher Scientific). All antibodies were purchased from BD Biosciences, BioLegend or eBioscience. Single-stain samples and fluorescence minus one (FMO) controls were used to establish PMT voltages, gating and compensation parameters. Cells were processed using a BD LSRFortessa or BD LSRFortessa X20 flow cytometer and analyzed using FlowJo software (Tree Star). Gates defining all populations were based on in silico-derived gates for all described proteins with the exception of CD127 in the CD4 SM, CD8 SM, CD4 Mat and CD8 Mat populations. In these cases, the CD127 fluorescent antibody did not have comparable sensitivity to the CD127 CITE-seq measurement and was therefore excluded.

### Differential expression analysis of positively selecting thymocytes with totalVI

Temporal features (that is, features that are differentially expressed over time) were determined by a totalVI one-vs-all DE test within each lineage between binned units of pseudotime. DE criteria (as above) included filters for significance (log(Bayes factor) >2.0 for genes, log(Bayes factor) > 1.0 for proteins), effect size (median log fold change >0.2 for both genes and proteins), and the proportion of expressing cells (detected expression in > 5% of the relevant population for genes). Top temporal genes were selected as the unique set among the top three differentially expressed genes per time that were differentially expressed in both lineages.

Differences between lineages were determined by a totalVI within-cluster DE test, where clusters were binned units in pseudotime and the condition was lineage assignment (that is, cells within a given unit of pseudotime were compared between lineages). Criteria for DE were the same as above.

To cluster differentially expressed genes into patterns, totalVI denoised gene expression values were standard scaled, reduced dimensions across cells using PCA, and clustered genes using the Leiden algorithm^60^ as implemented by Scanpy^59^. For features differentially expressed between lineages, the genes upregulated within a lineage were clustered according to expression within the lineage in which they were upregulated.

To test for enrichment of TCR signaling in differentially expressed gene clusters, we performed a hypergeometric test (phyper). TCR signaling genes were compiled from Netpath^34^ and a set of genes activated upon stimulation in DP thymocytes^64^. The background set included all genes considered in DE analysis. *P* values were adjusted by the Benjamini-Hochberg procedure.

### Transcription factor enrichment analysis

To perform transcription factor enrichment analysis with ChEA3^33^, we first selected target gene sets as genes differentially upregulated in one lineage relative to the other in each unit of pseudotime, filtered for significance (log(Bayes factor) >2.0), effect size (median log-transformed fold change >0.2), and detected expression in >5% of the population of interest. For each target gene set, transcription factors were scored for enrichment by the integrated mean ranking across all ChEA3 gene set libraries (MeanRank) based on the top performance of this ranking method^33^. ChEA3 analysis on gene clusters was performed as above, but using gene clusters as the target gene set.

To generate an overall ranking of transcription factors for their likely involvement in CD4-CD8 lineage commitment, we focused on enrichment in the three units of pseudotime before master regulator differential expression in each lineage (that is, in the CD4^+^ T cell lineage, the relevant pseudotime units are 4, 5 and 6, before the differential expression of *Zbtb7b* differential expression at pseudotime 7; in the CD8^+^ T cell lineage, the relevant pseudotime units are 5, 6 and 7, before the differential expression of *Runx3* at pseudotime 8). We excluded the pseudotime unit containing master regulator differential expression from the ranking, as genes differentially expressed at this time could be the result of the master regulator itself enforcing lineage-specific changes rather than the factors driving initial commitment to a lineage. The pseudotime unit containing master regulator differential expression is included in Fig. 4e for visualization, but did not contribute to the ranked order of transcription factors. We also excluded earlier units of pseudotime since these times included very few ( < 15) significantly different genes between the lineages. Finally, pseudotime bins in which a transcription factor was not expressed in at least 5% of the population of interest, did not contribute towards that transcription factor’s ranking. The overall ranking of candidate driver transcription factors was then generated by taking the mean of ranks across the relevant pseudotime units. Note that lower rank and lower score are better (meaning more enrichment).

Transcription factors were annotated by whether they had a known association with TCR signaling. A list of molecules involved in TCR signaling were curated from the NetPath database of molecules involved in the TCR signaling pathway and the NetPath database of genes transcriptionally upregulated by the TCR signaling pathway^34^. Additional genes related to TCR signaling were curated from literature sources^49,65–68^. Transcription factors were also annotated by whether they were known to target either *Gata3*, *Zbtb7b* or *Runx3* according to ChEA3 databases (that is, *Gata3*, *Zbtb7b* or *Runx3* appeared in the overlapping gene list for the transcription factor of interest in any ChEA3 query).

### Neonatal thymic slice experiments

For neonatal thymic slice experiments, postnatal day 1 (P1) wild-type, MHCI^−/−^ or MHCII^−/−^ male and female mice (described above) were used.

Thymic slices were prepared as previously described^69,70^, with minor modifications to adjust for the smaller size of neonatal thymi compared to those of adults. Thymic lobes were dissected, removed of connective tissue, embedded in 4% low melting point agarose (GTG-NuSieve Agarose, Lonza) and sectioned into 500 μM slices using a vibratome (VT1000S, Leica). Slices were placed onto 0.4 μM transwell inserts (Corning, 353090) and cultured in 6-well tissue culture plates containing 1 mL of complete RPMI medium (RPMI-1640 (Corning), 10% FBS (Thermo Fisher Scientific), 100 U ml^−1^ penicillin/streptomycin (Gibco), 1X L-glutamine (Gibco), 55 µM 2-mercaptoethanol (Gibco). Slices were cultured for indicated periods of time at 37 °C, 5% CO_2_, before being prepared and analyzed by flow cytometry. Due to the practical limitations of using a single pup as a biological replicate, a litter of pups were harvested for thymic slices (approximately six pups/litter and four slices/pup) and three or four thymic slices were randomly allocated to each condition. For neonatal slice data in Figs. 5 and 6, each dot represented a single thymic slice (*n* = a slice). Statistical analysis was conducted on slices pooled from independent experiments. For neonatal slice cultures containing Cyclosporin A (CsA; Millipore-Sigma, 239835), CsA was serially diluted to indicated concentrations (50–800 ng ml^−1^) and added directly to the culture medium. FK506 (Tacrolimus; InvivoGen, inh-fk5-5) and U0126 (InvivoGen, tlrl-u0126) were serially diluted in indicated concentrations (0.39-6.3 ng ml^-1^ and 0.63-10 µg ml^−1^, respectively) and added directly to culture medium.

Thymic slices were mechanically dissociated into a single-cell suspension, then filtered, washed and counted before being stained with a live dead/stain; Propidium Iodine (BioLegend), Ghost Violet 510 (Tonbo), Zombie NIR, or Zombie UV Fixable Viability Kit (BioLegend). Samples were blocked with anti-CD16/32 (2.4G2) and stained with surface antibodies against CD4, CD8, TCRβ, and CD69 in FACS buffer (1% BSA in PBS) containing Brilliant Stain Buffer Plus (BD Biosciences). Intracellular staining for GATA3, RUNX3, and THPOK was performed using the eBioscience FoxP3/Transcription Factor Staining Buffer Set (Thermo Fisher Scientific). All antibodies were purchased from BD Biosciences, BioLegend or eBioscience. Single-stain samples and fluorescence minus one (FMO) controls were used to establish PMT voltages, gating and compensation parameters. Cells were processed using a BD LSRFortessa or BD LSRFortessa X20 flow cytometer and analyzed using FlowJo software (Tree Star).

### Computational multidimensional analyses of flow cytometry data

FCS files were loaded into python using flowIO. Compensation was performed using manually determined compensation values. Data was loaded into Scanpy^59^ for further processing. Permissive manual gating in python was performed using physical dimension (FSC-W, FSC-A, SSC-A) on manual inspection and dead cells were filtered out based on live/dead staining. Fluorescent channels were normalized to a range of [0, 1]. Clustering was performed using PARC^71^ with a resolution_parameter = 1.5, keep_all_local_dist=False and jac_std_global = 0.15. This yielded 34 clusters. Clusters were merged based on manual inspections of all fluorescent channels and merging was not performed if at least one fluorescent channel was differentially expressed between two clusters. We used PAGA^72^ initialization for UMAP embedding. PAGA was computed using 30 nearest neighbors in expression space using cosine distance. For UMAP embedding, we used the following parameters: n_neighbors = 30, metric=’euclidean’, min_dist = 0.3, init_pos=’paga’. For display of proportional changes in cluster frequency we divided the number of cells in each cluster by the total number of cells in the respective sample. We divided those values by the mean over the proportion in the respective cluster in the no drug sample and took the logarithm of this ratio to yield the log fold enrichment of the respective cluster. Seaborn was used for visualization. All computational gates were validated by manual inspection in FlowJo.

### In vivo EdU labeling and calcineurin blockade in adult mice

Four- to eight-week-old male and female AND *Rag1*^−/−^ mice (described above) were intraperitoneally injected with 2 mg EdU (Thermo Fisher Scientific, A10044) in the evening. The next morning (16 h later), mice were injected with 5 μg FK506 (Invitrogen, INH-FK5-5). Thymi were taken for flow cytometry 24 or 48 h after FK506 was administered. Thymi were dissociated and 2 × 10^6^ cells were surface stained for flow cytometry as described above. After surface staining, cells were split, and 1 × 10^6^ were processed using Click-iT EdU Pacific Blue Flow Cytometry Assay Kit (Thermo Fisher Scientific, C10418). The other 1 × 10^6^ were subjected to anti-CD3 crosslinking and p-ERK staining as described below. Flow cytometry and data analysis were performed as described above.

### In vitro TCR activation and staining for p-ERK

Approximately 1 × 10^6^ surface stained thymocytes were washed and resuspended in approximately 240 μl serum-free media. Per sample, 10 μl anti-CD3e antibody (clone 145-2C11, Invitrogen 14-0031-85) was added to reach a final concentration of 20 mg ml^−1^. Working quickly, 7 μl of anti-Armenian hamster IgG crosslinker (Jackson ImmunoResearch Laboratories 127-0051-160) was added to each sample, briefly vortexed, and placed in a 37 °C water bath for 3 min for in vitro TCR activation. For fixation, 1:1 volume of 4% paraformaldehyde was added to each tube and incubated at room temperature for 10 min before being washed in PBS. Cells were resuspended in 900 μl ice-cold methanol by gentle pipetting, and incubated on ice for 30 min. After three washes in PBS, cells were incubated at 4 °C overnight p-ERK antibody (1:20 dilution, BioLegend, 675504). Samples were washed and resuspended for analysis. Flow cytometry and data analysis were performed as described above.

### Statistical analyses

Data were analyzed using Prism software (GraphPad). Comparisons were performed using an unpaired *t*-test, one- or two-way analysis of variance, where indicated in the figure legends. For all statistical models and tests described above, the significance is displayed as follows: *P < 0.05, **P < 0.01, ***P < 0.001, ****P < 0.0001. Animals were randomly assigned to experimental or control groups. No statistical methods were used to pre-determine sample sizes but our sample sizes are similar to those reported in previous publications^7,31^. Data distribution was assumed to be normal, but this was not formally tested. No animals or data points were excluded from the analyses.

### Reporting summary

Further information on research design is available in the Nature Portfolio Reporting Summary linked to this article.

## Online content

Any methods, additional references, Nature Portfolio reporting summaries, source data, extended data, supplementary information, acknowledgements, peer review information; details of author contributions and competing interests; and statements of data and code availability are available at 10.1038/s41590-023-01584-0.

### Supplementary information

Supplementary Information Supplementary Information contains a resource of protein and gene expression over pseudotime by genotype. Features are totalVI denoised expression values scaled per feature and smoothed by loess curves. Proteins include all proteins differentially expressed between lineages or over pseudotime in either lineage with criteria for significance (log(Bayes factor) > 0.5) and effect size (median log fold change > 0.2). RNA includes all genes differentially expressed between lineages or over pseudotime in either lineage with criteria for significance (log(Bayes factor) > 2.0), effect size (median log fold change > 0.2), and the proportion of expressing cells (detected expression in > 5% of the relevant population)

Reporting Summary

Supplementary Data 1 Antibodies used in this study.

Supplementary Data 2 CITE-seq sample information.

Supplementary Data 3 DE test results for totalVI one-versus-all DE test between annotated thymus populations.

Supplementary Data 4 Lineage information by genotype.

Supplementary Data 5 DE test results for totalVI DE test across pseudotime within the CD4^+^ T cell lineage.

Supplementary Data 6 DE test results for totalVI DE test across pseudotime within the CD8^+^ T cell lineage.

Supplementary Data 7 DE test results for totalVI DE test within pseudotime and between CD4^+^ and CD8^+^ T cell lineages.

Supplementary Data 8 Cluster assignments for genes upregulated in the CD4^+^ T cell lineage from the totalVI DE test within pseudotime and between CD4^+^ and CD8^+^ T cell lineages.

Supplementary Data 9 Cluster assignments for genes upregulated in the CD8^+^ T cell lineage from the totalVI DE test within pseudotime and between CD4^+^ and CD8^+^ T cell lineages.

Supplementary Data 10 ChEA3 results for the CD4^+^ T cell lineage by pseudotime.

Supplementary Data 11 ChEA3 test results for the CD8^+^ T cell lineage by pseudotime.

Supplementary Data 12 ChEA3 test results for the CD4^+^ T cell lineage by gene cluster.

Supplementary Data 13 ChEA3 test results for the CD8^+^ T cell lineage by gene cluster.

## Data Availability

CITE-seq data discussed in this manuscript have been deposited in the NCBI Gene Expression Omnibus (GEO) and are accessible through accession number GSE186078. The data can be explored interactively with Vision at http://s133.cs.berkeley.edu:9001/Results.html (positive selection subset) and http://s133.cs.berkeley.edu:9002/Results.html (full dataset).

## Code availability

The code to reproduce the results in this manuscript is available at https://github.com/YosefLab/Thymus_CITE-seq and has been deposited at 10.5281/zenodo.8102050 (ref. ^73^).

## References

[1] Germain RN (2002). T-cell development and the CD4–CD8 lineage decision. Nat. Rev. Immunol..

[2] Xiong Y, Bosselut R (2012). CD4–CD8 differentiation in the thymus: connecting circuits and building memories. Curr. Opin. Immunol..

[3] Singer A (2008). Lineage fate and intense debate: Myths, models and mechanisms of CD4- versus CD8-lineage choice. Nat. Rev. Immunol..

[4] Shinzawa M (2022). Reversal of the T cell immune system reveals the molecular basis for T cell lineage fate determination in the thymus. Nat. Immunol..

[5] Kisielow P, Miazek A (1995). Positive selection of T cells: rescue from programmed cell death and differentiation require continual engagement of the T cell receptor. J. Exp. Med..

[6] Liu X, Bosselut R (2004). Duration of TCR signaling controls CD4-CD8 lineage differentiation in vivo. Nat. Immunol..

[7] Au-Yeung BB (2014). Quantitative and temporal requirements revealed for Zap70 catalytic activity during T cell development. Nat. Immunol..

[8] Sinclair C, Seddon B (2014). Overlapping and asymmetric functions of TCR Signaling during Thymic Selection of CD4 and CD8 Lineages. J. Immunol..

[9] McNeil LK, Starr TK, Hogquist KA (2005). A requirement for sustained ERK signaling during thymocyte positive selection in vivo. Proc. Natl Acad. Sci. USA.

[10] Taniuchi I (2016). Views on helper/cytotoxic lineage choice from a bottom-up approach. Immunol. Rev..

[11] Park JE (2020). A cell atlas of human thymic development defines T cell repertoire formation. Science (1979).

[12] Lavaert M (2020). Integrated scRNA-Seq identifies human postnatal thymus seeding progenitors and regulatory dynamics of differentiating immature thymocytes. Immunity.

[13] Zhou W (2019). Single-cell analysis reveals regulatory gene expression dynamics leading to lineage commitment in early T cell development. Cell Syst..

[14] Chopp LB (2020). An integrated epigenomic and transcriptomic map of mouse and human ab T cell development article an integrated epigenomic and transcriptomic map of mouse and human ab T cell development. Immunity.

[15] Karimi MM (2021). The order and logic of CD4 versus CD8 lineage choice and differentiation in mouse thymus. Nat. Commun..

[16] Stoeckius M (2017). Simultaneous epitope and transcriptome measurement in single cells. Nat. Methods.

[17] Gayoso A (2021). Joint probabilistic modeling of single-cell multi-omic data with totalVI. Nat. Methods.

[18] Hogquist K, Xing Y, Hsu F-C, Shapiro VS (2015). T cell adolescence: Maturation events beyond positive selection. J. Immunol..

[19] Street K (2018). Slingshot: Cell lineage and pseudotime inference for single-cell transcriptomics. BMC Genomics.

[20] Saelens W, Cannoodt R, Todorov H, Saeys Y (2019). A comparison of single-cell trajectory inference methods. Nat. Biotechnol..

[21] Saini M (2010). Regulation of Zap70 expression during thymocyte development enables temporal separation of CD4 and CD8 repertoire selection at different signaling thresholds. Sci. Signal..

[22] Hu Q (2012). Examination of thymic positive and negative selection by flow cytometry. J. Vis. Exp..

[23] Lundberg K, Heath W, Köntgen F, Carbone FR, Shortman K (1995). Intermediate steps in positive selection: differentiation of CD4^+^8^int^ TCRint thymocytes into CD4-8^+^TCR^hi^ thymocytes. J. Exp. Med..

[24] Lucas B, Germain RN (1996). Unexpectedly complex regulation of CD4/CD8 coreceptor expression supports a revised model for CD4^+^CD8^+^ thymocyte differentiation. Immunity.

[25] Chan SH, Cosgrove D, Waltzinger C, Benoist C, Mathis D (1993). Another view of the selective model of thymocyte selection. Cell.

[26] Marodon G, Rocha B (1994). Generation of mature T cell populations in the thymus: CD4 or CD8 down-regulation occurs at different stages of thymocyte differentiation. Eur. J. Immunol..

[27] Wang L (2008). Distinct functions for the transcription factors GATA-3 and ThPOK during intrathymic differentiation of CD4^+^ T cells. Nat. Immunol..

[28] Egawa T, Littman DR (2008). ThPOK acts late in specification of the helper T cell lineage and suppresses Runx-mediated commitment to the cytotoxic T cell lineage. Nat. Immunol..

[29] Kovanen PE (2008). T-cell development and function are modulated by dual specificity phosphatase DUSP5. J. Biol. Chem..

[30] Tanzola MB, Kersh GJ (2006). The dual specificity phosphatase transcriptome of the murine thymus. Mol. Immunol..

[31] Lutes LK (2021). T cell self-reactivity during thymic development dictates the timing of positive selection. Elife.

[32] Choi S, Cornall R, Lesourne R, Love PE (2017). THEMIS: Two models, different thresholds. Trends Immunol..

[33] Keenan AB (2019). ChEA3: transcription factor enrichment analysis by orthogonal omics integration. Nucleic Acids Res..

[34] Kandasamy K (2010). NetPath: A public resource of curated signal transduction pathways. Genome Biol..

[35] Navarro MN, Cantrell DA (2014). Serine-threonine kinases in TCR signaling. Nat. Immunol..

[36] Hogquist KA, Jameson SC (2014). The self-obsession of T cells: how TCR signaling thresholds affect fate ‘decisions’ and effector function. Nat. Immunol..

[37] Malissen B, Grégoire C, Malissen M, Roncagalli R (2014). Integrative biology of T cell activation. Nat. Immunol..

[38] Chakraborty AK, Weiss A (2014). Insights into the initiation of TCR signaling. Nat. Immunol..

[39] Gimferrer I (2011). Regulation of GATA-3 expression during CD4 lineage differentiation. J. Immunol..

[40] Scheinman EJ, Avni O (2009). Transcriptional regulation of Gata3 in T helper cells by the integrated activities of transcription factors downstream of the interleukin-4 receptor and T cell receptor. J. Biol. Chem..

[41] Gallo EM (2007). Calcineurin sets the bandwidth for discrimination of signals during thymocyte development. Nature.

[42] Lucas B, Vasseur F, Penit C (1993). Normal sequence of phenotypic transitions in one cohort of 5-bromo-2’-deoxyuridine-pulse-labeled thymocytes. Correlation with T cell receptor expression. J. Immunol..

[43] Wang CR (1995). T cell receptor-mediated signaling events in CD4^+^CD8^+^ thymocytes undergoing thymic selection: requirement of calcineurin activation for thymic positive selection but not negative selection. J. Exp. Med..

[44] Muroi S (2008). Cascading suppression of transcriptional silencers by ThPOK seals helper T cell fate. Nat. Immunol..

[45] Sharp LL, Schwarz DA, Bott CM, Marshall CJ, Hedrick SM (1997). The influence of the MAPK pathway on T cell lineage commitment. Immunity.

[46] Wilkinson B, Kaye J (2001). Requirement for sustained MAPK signaling in both CD4 and CD8 lineage commitment: A threshold model. Cell Immunol..

[47] Daniels MA (2006). Thymic selection threshold defined by compartmentalization of Ras/MAPK signalling. Nature.

[48] Webb LV, Ley SC, Seddon B (2016). TNF activation of NF-κB is essential for development of single-positive thymocytes. J. Exp. Med..

[49] Wang L, Xiong Y, Bosselut R (2010). Tenuous paths in unexplored territory: From T cell receptor signaling to effector gene expression during thymocyte selection. Semin. Immunol..

[50] Steier, Z., Kim, E. J. Y. K., Aylard, D. A. & Robey, E. A. The CD4 versus CD8 T cell fate decision: a multiomics-informed perspective. *Annu. Rev. Immunol.* **42**, 10.1146/annurev-immunol-083122-040929 (2024). 10.1146/annurev-immunol-083122-040929 38271641

[51] Becht E (2019). Dimensionality reduction for visualizing single-cell data using UMAP. Nat. Biotechnol..

[52] Grusby MJ, Johnson RS, Papaioannou VE, Glimcher LH (1991). Depletion of CD4^+^ T cells in major histocompatibility complex class II-deficient mice. Science.

[53] Kaye J (1989). Selective development of CD4^+^ T cells in transgenic mice expressing a class II MHC-restricted antigen receptor. Nature.

[54] Mamalaki C (1992). Thymic depletion and peripheral activation of class I major histocompatibility complex-restricted T cells by soluble peptide in T-cell receptor transgenic mice. Proc. Natl Acad. Sci. USA.

[55] Zheng GXY (2017). Massively parallel digital transcriptional profiling of single cells. Nat. Commun..

[56] Dobin A (2013). STAR: ultrafast universal RNA-seq aligner. Bioinformatics.

[57] Stuart T (2019). Comprehensive Integration of Single-Cell Data. Cell.

[58] Lopez R, Regier J, Cole MB, Jordan MI, Yosef N (2018). Deep generative modeling for single-cell transcriptomics. Nat. Methods.

[59] Wolf FA, Angerer P, Theis FJ (2018). SCANPY: Large-scale single-cell gene expression data analysis. Genome Biol..

[60] Traag VA, Waltman L, van Eck NJ (2019). From Louvain to Leiden: guaranteeing well-connected communities. Sci. Rep..

[61] DeTomaso D (2019). Functional interpretation of single cell similarity maps. Nat. Commun..

[62] Daley SR, Hu DY, Goodnow CC (2013). Helios marks strongly autoreactive CD4^+^ T cells in two major waves of thymic deletion distinguished by induction of PD-1 or NF-κB. J. Exp. Med..

[63] Xing Y, Wang X, Jameson SC, Hogquist KA (2016). Late stages of T cell maturation in the thymus involve NF-κB and tonic type I interferon signaling. Nat. Immunol..

[64] Mingueneau M, Jiang W, Feuerer M, Mathis D, Benoist C (2012). Thymic negative selection is functional in NOD mice. J. Exp. Med..

[65] Shao H, Kono DH, Chen LY, Rubin EM, Kaye J (1997). Induction of the early growth response (Egr) family of transcription factors during thymic selection. J. Exp. Med..

[66] Wong WF (2014). T-cell receptor signaling induces proximal Runx1 transactivation via a calcineurin–NFAT pathway. Eur. J. Immunol..

[67] López-Rodríguez C, Aramburu J, Berga-Bolaños R (2015). Transcription factors and target genes of pre-TCR signaling. Cell. Mol. Life Sci..

[68] Hedrick SM, Michelini RH, Doedens AL, Goldrath AW, Stone EL (2012). FOXO transcription factors throughout T cell biology. Nat. Rev. Immunol..

[69] Dzhagalov IL, Melichar HJ, Ross JO, Herzmark P, Robey EA (2012). Two-photon imaging of the immune system. Curr. Protoc. Cytom..

[70] Ross JO, Melichar HJ, Halkias J, Robey EA (2015). Studying T cell development in thymic slices. T-Cell Dev.: Methods Protoc..

[71] Stassen SV (2020). PARC: ultrafast and accurate clustering of phenotypic data of millions of single cells. Bioinformatics.

[72] Wolf FA (2019). PAGA: graph abstraction reconciles clustering with trajectory inference through a topology preserving map of single cells. Genome Biol..

[73] Steier, Z. YosefLab/Thymus_CITE-seq: Thymus_CITE-seq Reproducibility (v1.0.0). *Zenodo*10.5281/zenodo.8102050 (2023).

[74] Liberzon A (2011). Molecular signatures database (MSigDB) 3.0. Bioinformatics.

