# Supplementary material for: Single-cell multiomic analysis of thymocyte development reveals drivers of CD4+ T cell and CD8+ T cell lineage commitment
Source: Nat Immunol. 2023 Aug 14;24(9):1579–90. doi: 10.1038/s41590-023-01584-0 (PMC10457207; doi:10.1038/s41590-023-01584-0)
Supplement: Supplementary file 1 — Supplementary Information contains a resource of protein and gene expression over pseudotime by genotype. Features are totalVI denoised expression values scaled per feature and smoothed by loess curves. Proteins include all proteins differentially expressed between lineages or over pseudotime in either lineage with criteria for significance (log(Bayes factor) > 0.5) and effect size (median log fold change > 0.2). RNA includes all genes differentially expressed between lineages or over pseudotime in either lineage with criteria for significance (log(Bayes factor) > 2.0), effect size (median log fold change > 0.2), and the proportion of expressing cells (detected expression in > 5% of the relevant population) [file 41590_2023_1584_MOESM1_ESM.pdf]

nature immunology

Resource

https://doi.org/10.1038/s41590-023-01584-0

Single-cell multiomic analysis of thymocyte development reveals drivers of CD4^{+} T cell and CD8^{+} T cell lineage commitment

In the format provided by the authors and unedited

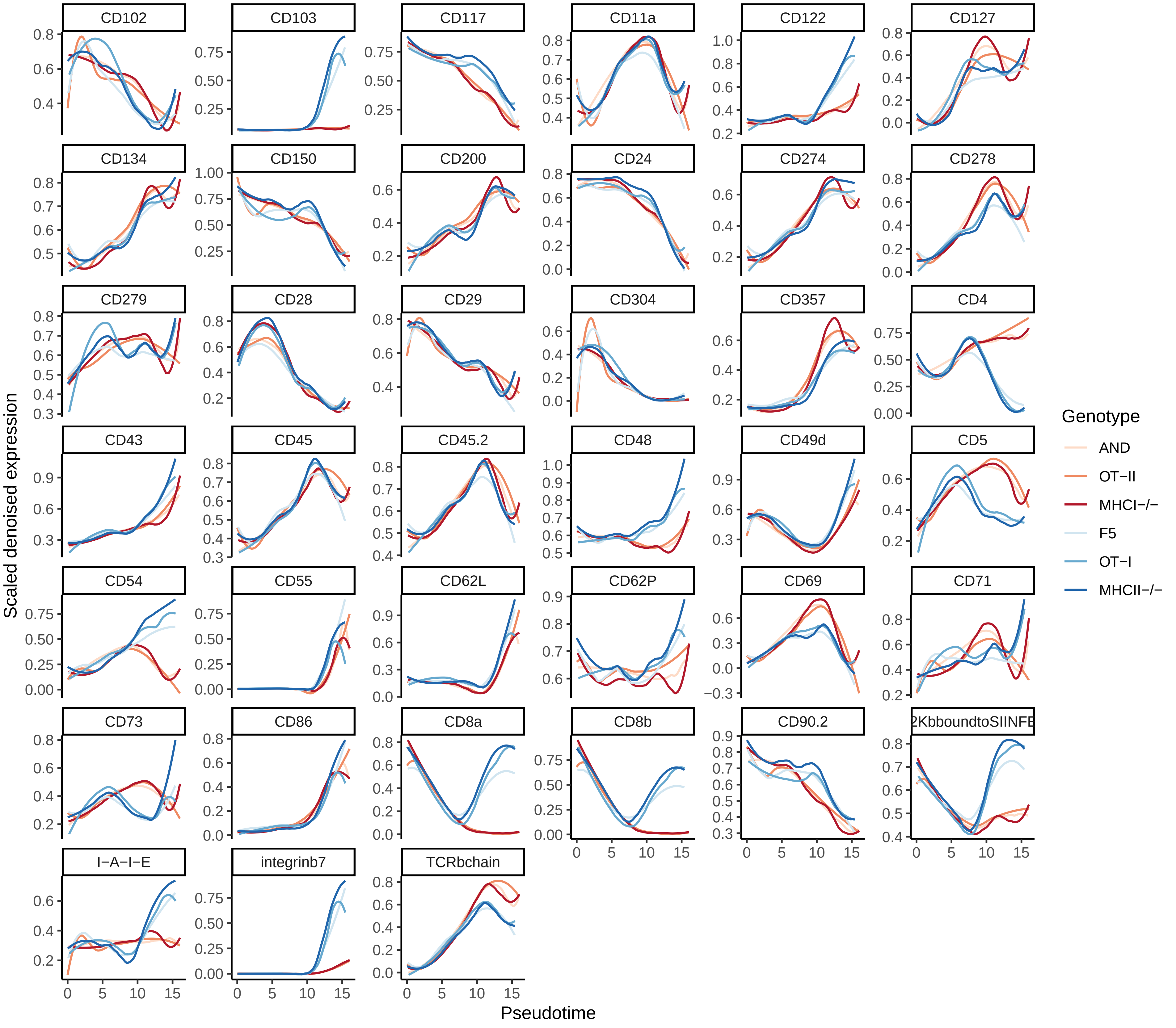

CD102
CD103
CD117
CD11a
CD122
CD127
CD134
CD150
CD200
CD24
CD274
CD278
CD279
CD28
CD29
CD304
CD357
CD4
CD43
CD45
CD45.2
CD48
CD49d
CD5
CD54
CD55
CD62L
CD62P
CD69
CD71
CD73
CD86
CD8a
CD8b
CD90.2
2KbboundtoSIINFE
I−A−I−E
integrinb7
TCRbchain
Scaled denoised expression
Pseudotime
Genotype
AND
OT−II
MHCI−/−
F5
OT−I
MHCII−/−

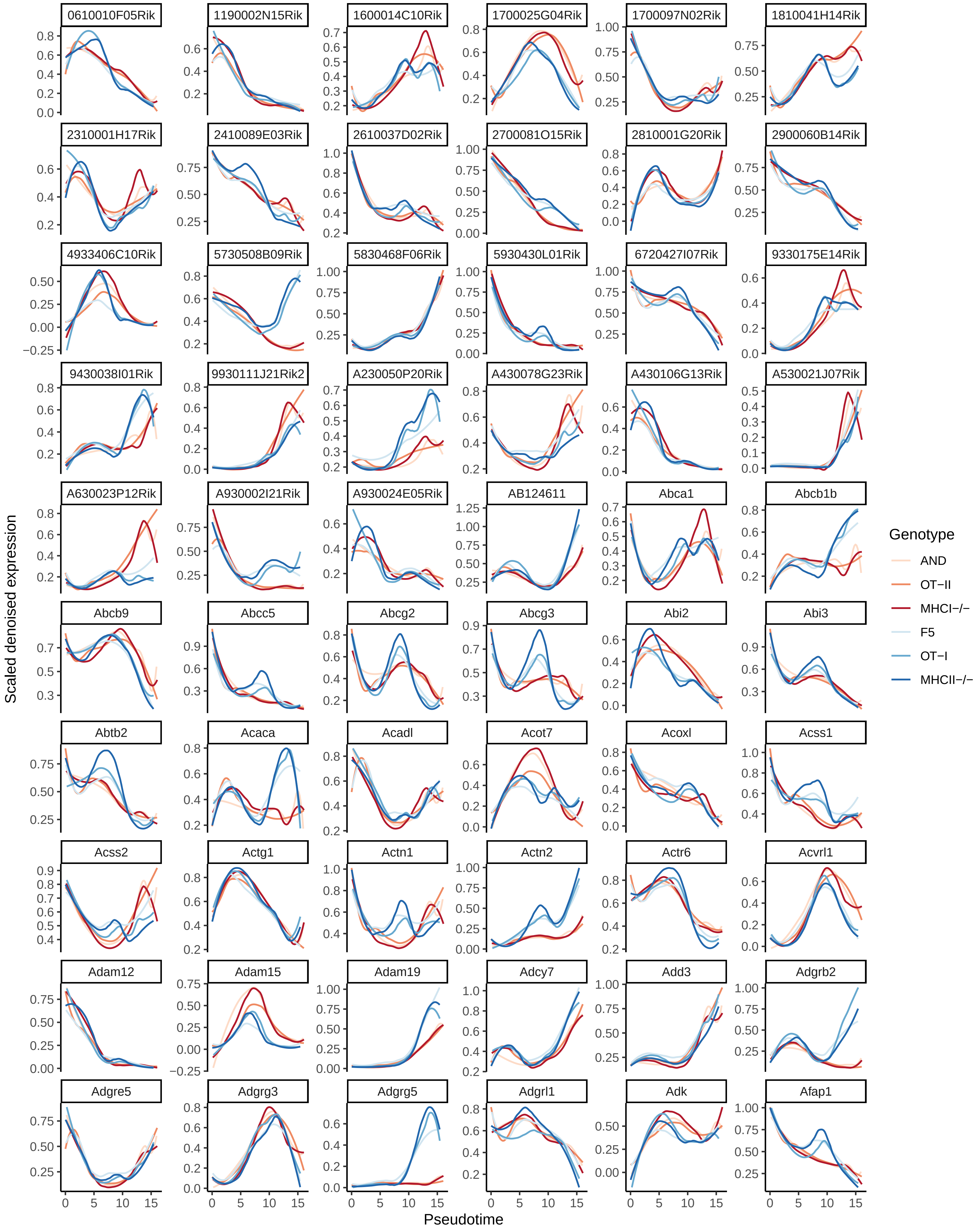
0610010F05Rik
1190002N15Rik
1600014C10Rik
1700025G04Rik
1700097N02Rik
1810041H14Rik
2310001H17Rik
2410089E03Rik
2610037D02Rik
2700081O15Rik
2810001G20Rik
2900060B14Rik
4933406C10Rik
5730508B09Rik
5830468F06Rik
5930430L01Rik
6720427I07Rik
9330175E14Rik
9430038I01Rik
9930111J21Rik2
A230050P20Rik
A430078G23Rik
A430106G13Rik
A530021J07Rik
A630023P12Rik
A930002I21Rik
A930024E05Rik
AB124611
Abca1
Abcb1b
Abcb9
Abcc5
Abcg2
Abcg3
Abi2
Abi3
Abtb2
Acaca
Acadl
Acot7
Acoxl
Acss1
Acss2
Actg1
Actn1
Actn2
Actr6
Acvrl1
Adam12
Adam15
Adam19
Adcy7
Add3
Adgrb2
Adgre5
Adgrg3
Adgrg5
Adgrl1
Adk
Afap1
Scaled denoised expression
Pseudotime
Genotype
AND
OT−II
MHCI−/−
F5
OT−I
MHCII−/−

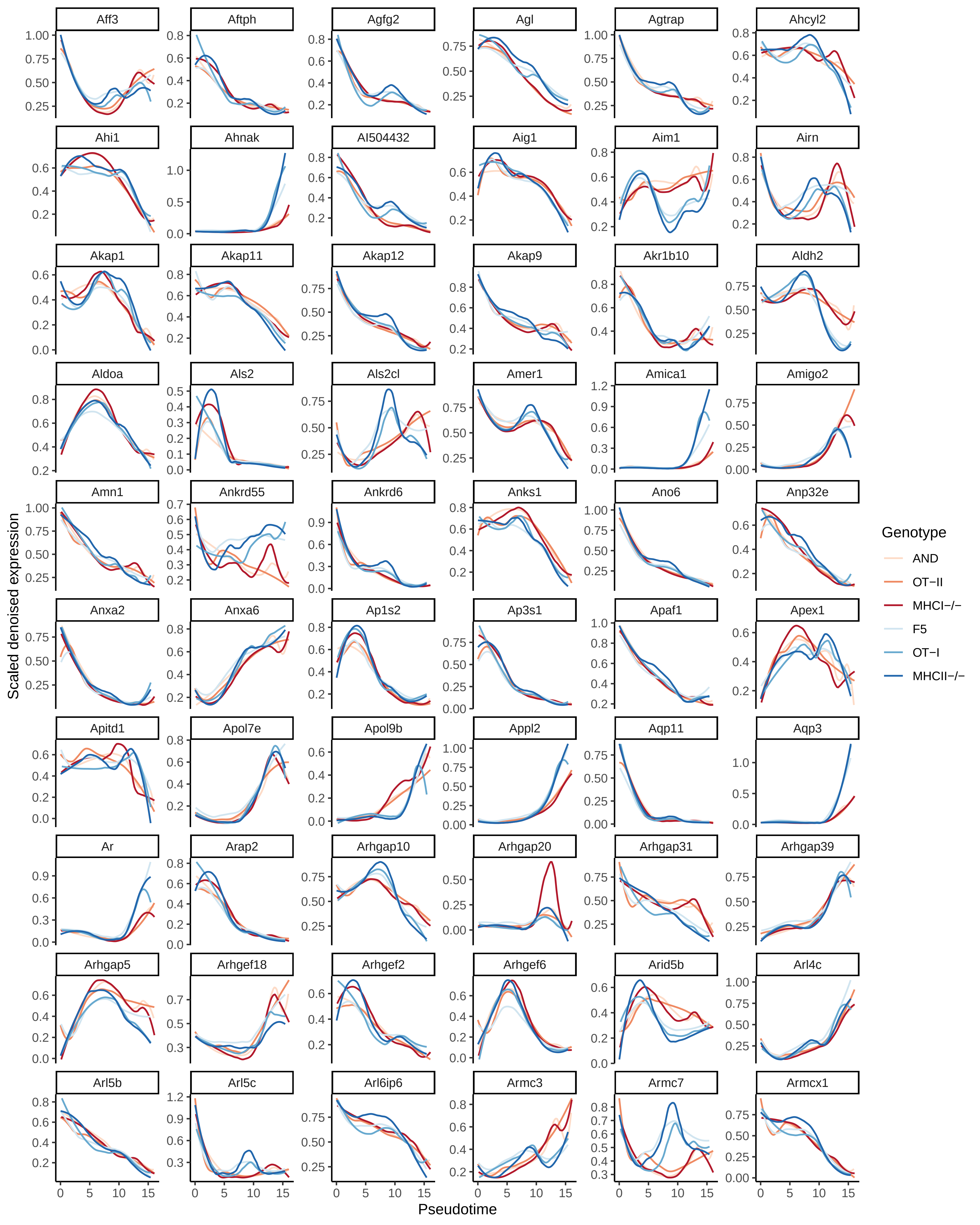

Aff3
Aftph
Agfg2
Agl
Agtrap
Ahcyl2
Ahi1
Ahnak
AI504432
Aig1
Aim1
Airn
Akap1
Akap11
Akap12
Akap9
Akr1b10
Aldh2
Aldoa
Als2
Als2cl
Amer1
Amica1
Amigo2
Amn1
Ankrd55
Ankrd6
Anks1
Ano6
Anp32e
Anxa2
Anxa6
Ap1s2
Ap3s1
Apaf1
Apex1
Apitd1
Apol7e
Apol9b
Appl2
Aqp11
Aqp3
Ar
Arap2
Arhgap10
Arhgap20
Arhgap31
Arhgap39
Arhgap5
Arhgef18
Arhgef2
Arhgef6
Arid5b
Arl4c
Arl5b
Arl5c
Arl6ip6
Armc3
Armc7
Armcx1
Scaled denoised expression
Pseudotime
Genotype
AND
OT−II
MHCI−/−
F5
OT−I
MHCII−/−

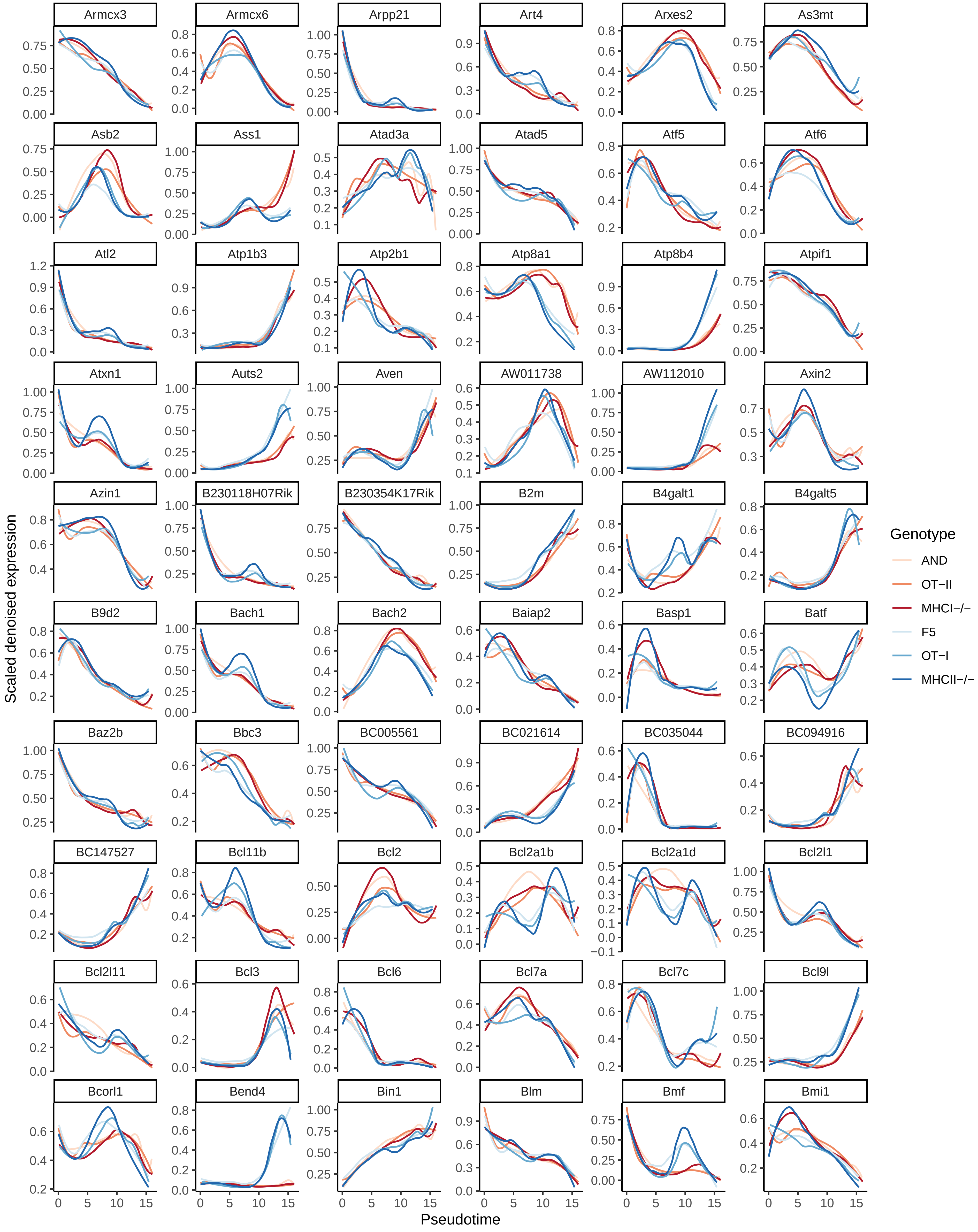
Armcx3
Armcx6
Arpp21
Art4
Arxes2
As3mt
Asb2
Ass1
Atad3a
Atad5
Atf5
Atf6
Atl2
Atp1b3
Atp2b1
Atp8a1
Atp8b4
Atpif1
Atxn1
Auts2
Aven
AW011738
AW112010
Axin2
Azin1
B230118H07Rik
B230354K17Rik
B2m
B4galt1
B4galt5
B9d2
Bach1
Bach2
Baiap2
Basp1
Batf
Baz2b
Bbc3
BC005561
BC021614
BC035044
BC094916
BC147527
Bcl11b
Bcl2
Bcl2a1b
Bcl2a1d
Bcl2l1
Bcl2l11
Bcl3
Bcl6
Bcl7a
Bcl7c
Bcl9l
Bcorl1
Bend4
Bin1
Blm
Bmf
Bmi1
Scaled denoised expression
Pseudotime
Genotype
AND
OT−II
MHCI−/−
F5
OT−I
MHCII−/−

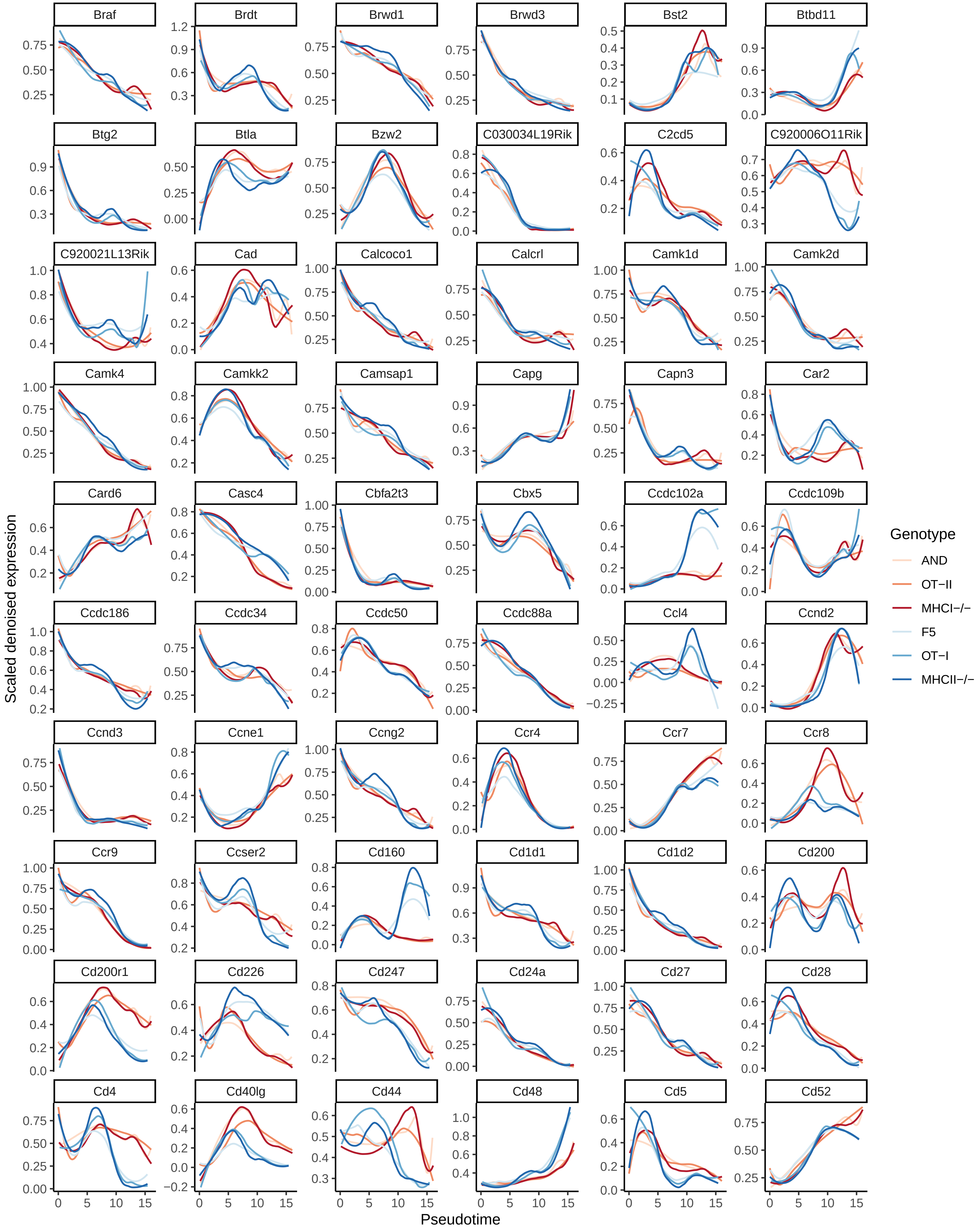
Braf
Brdt
Brwd1
Brwd3
Bst2
Btbd11
Btg2
Btla
Bzw2
C030034L19Rik
C2cd5
C920006O11Rik
C920021L13Rik
Cad
Calcoco1
Calcrl
Camk1d
Camk2d
Camk4
Camkk2
Camsap1
Capg
Capn3
Car2
Card6
Casc4
Cbfa2t3
Cbx5
Ccdc102a
Ccdc109b
Ccdc186
Ccdc34
Ccdc50
Ccdc88a
Ccl4
Ccnd2
Ccnd3
Ccne1
Ccng2
Ccr4
Ccr7
Ccr8
Ccr9
Ccser2
Cd160
Cd1d1
Cd1d2
Cd200
Cd200r1
Cd226
Cd247
Cd24a
Cd27
Cd28
Cd4
Cd40lg
Cd44
Cd48
Cd5
Cd52
Scaled denoised expression
Pseudotime
Genotype
AND
OT−II
MHCI−/−
F5
OT−I
MHCII−/−

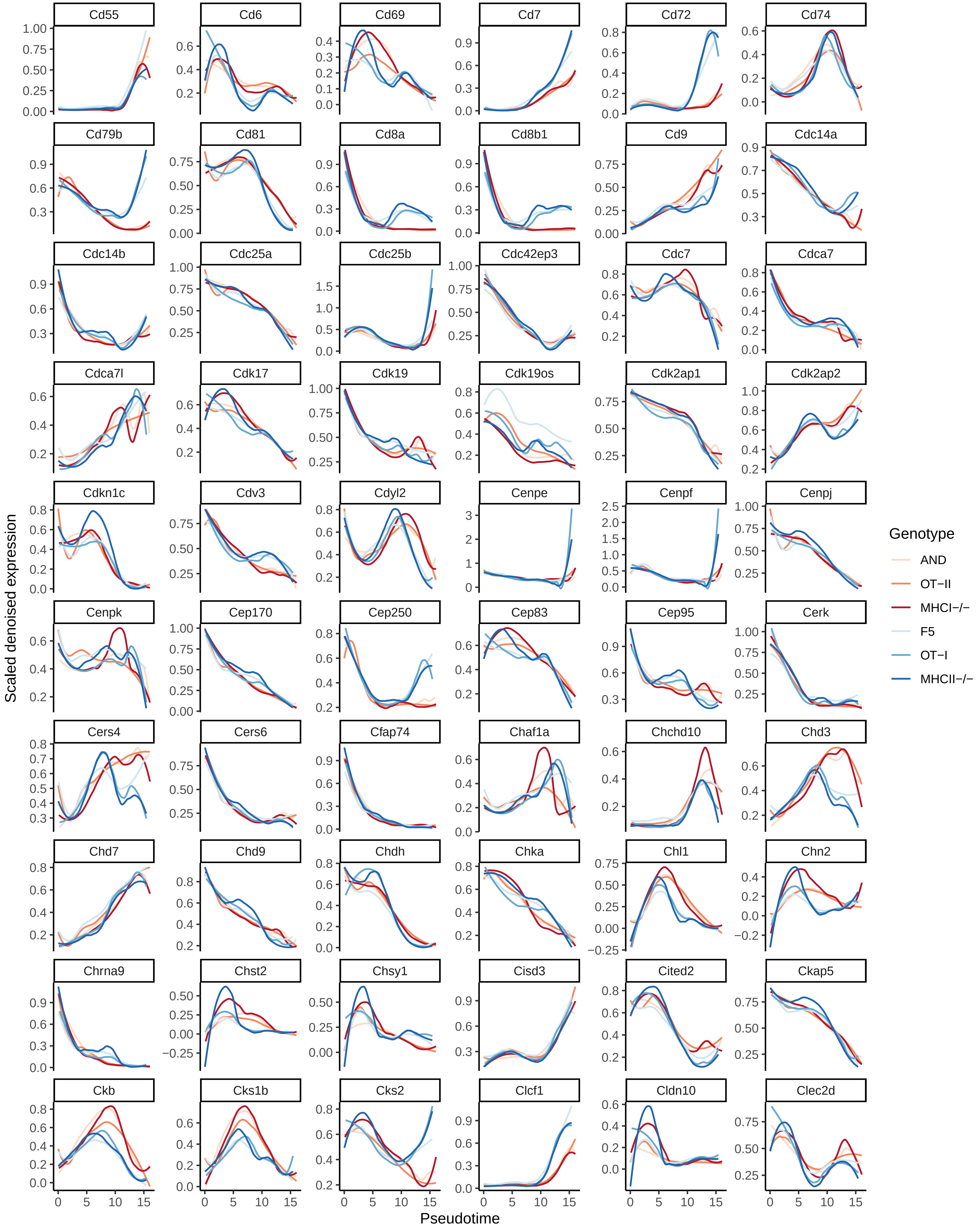
Cd55
Cd6
Cd69
Cd7
Cd72
Cd74
Cd79b
Cd81
Cd8a
Cd8b1
Cd9
Cdc14a
Cdc14b
Cdc25a
Cdc25b
Cdc42ep3
Cdc7
Cdca7
Cdca7l
Cdk17
Cdk19
Cdk19os
Cdk2ap1
Cdk2ap2
Cdkn1c
Cdv3
Cdyl2
Cenpe
Cenpf
Cenpj
Cenpk
Cep170
Cep250
Cep83
Cep95
Cerk
Cers4
Cers6
Cfap74
Chaf1a
Chchd10
Chd3
Chd7
Chd9
Chdh
Chka
Chl1
Chn2
Chrna9
Chst2
Chsy1
Cisd3
Cited2
Ckap5
Ckb
Cks1b
Cks2
Clcf1
Cldn10
Clec2d
Scaled denoised expression
Pseudotime
Genotype
AND
OT−II
MHCI−/−
F5
OT−I
MHCII−/−

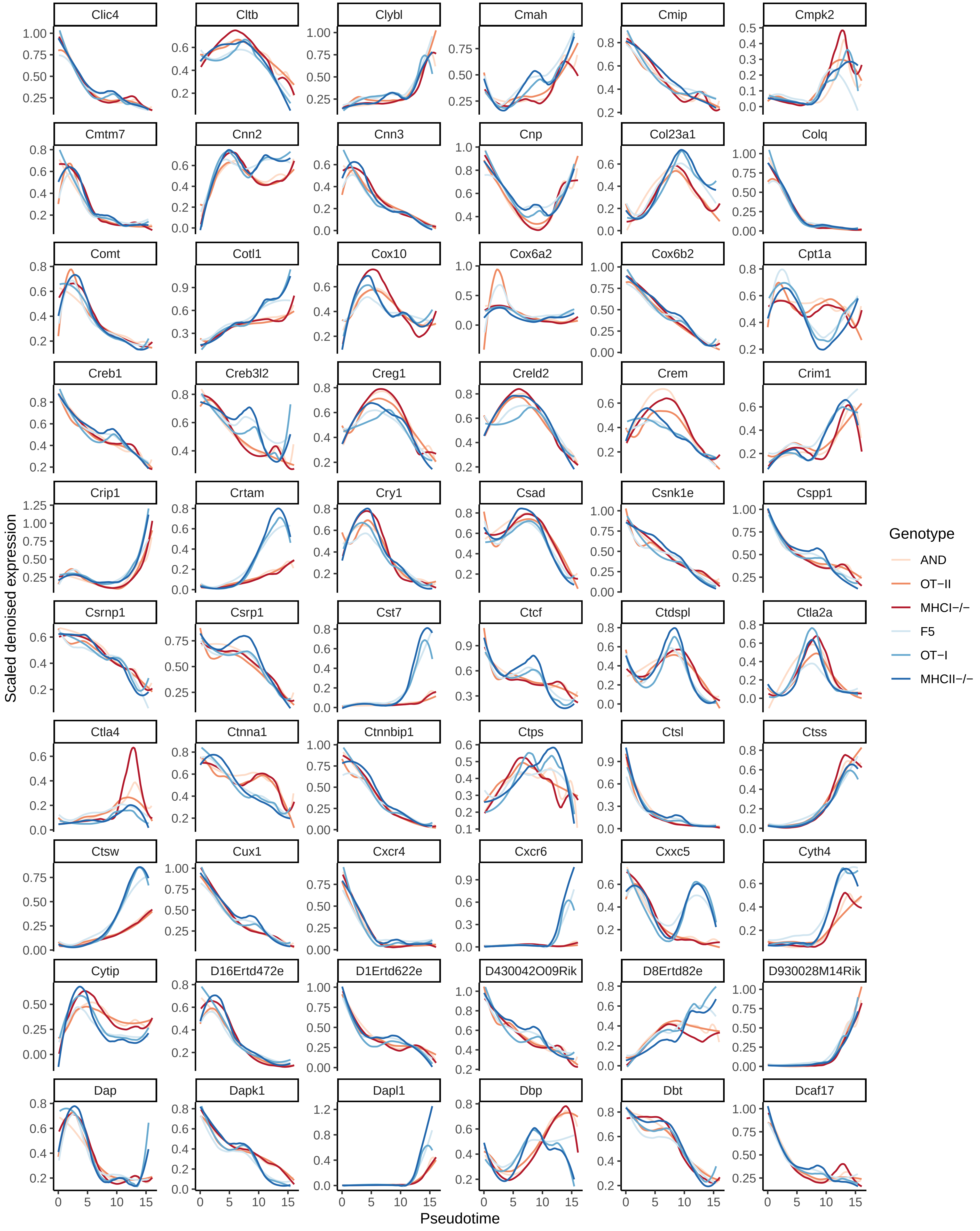

Clic4
Cltb
Clybl
Cmah
Cmip
Cmpk2
Cmtm7
Cnn2
Cnn3
Cnp
Col23a1
Colq
Comt
Cotl1
Cox10
Cox6a2
Cox6b2
Cpt1a
Creb1
Creb3l2
Creg1
Creld2
Crem
Crim1
Crip1
Crtam
Cry1
Csad
Csnk1e
Cspp1
Csrnp1
Csrp1
Cst7
Ctcf
Ctdspl
Ctla2a
Ctla4
Ctnna1
Ctnnbip1
Ctps
Ctsl
Ctss
Ctsw
Cux1
Cxcr4
Cxcr6
Cxxc5
Cyth4
Cytip
D16Ertd472e
D1Ertd622e
D430042O09Rik
D8Ertd82e
D930028M14Rik
Dap
Dapk1
Dapl1
Dbp
Dbt
Dcaf17
Scaled denoised expression
Pseudotime
Genotype
AND
OT−II
MHCI−/−
F5
OT−I
MHCII−/−

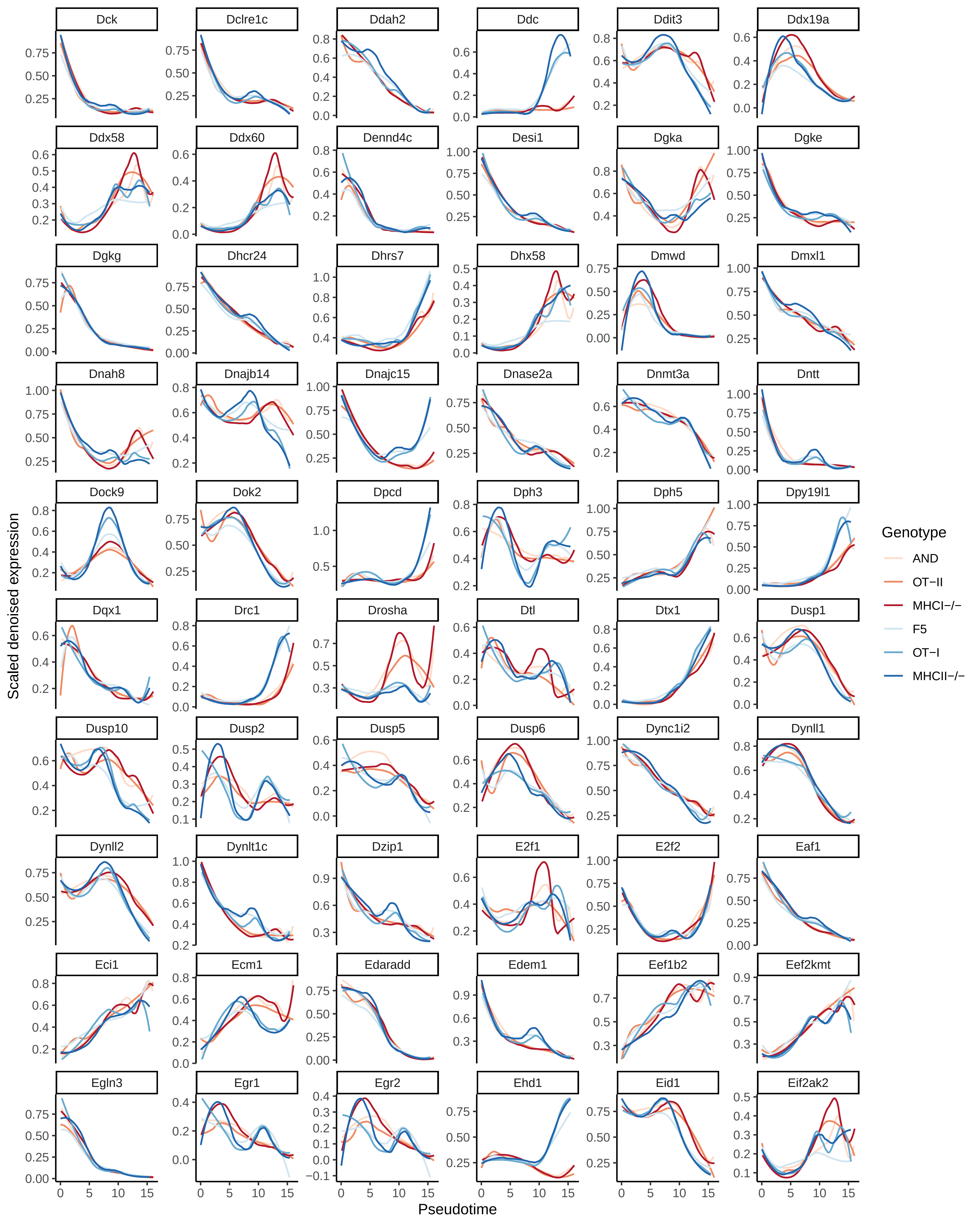

Dck
Dclre1c
Ddah2
Ddc
Ddit3
Ddx19a
Ddx58
Ddx60
Dennd4c
Desi1
Dgka
Dgke
Dgkg
Dhcr24
Dhrs7
Dhx58
Dmwd
Dmxl1
Dnah8
Dnajb14
Dnajc15
Dnase2a
Dnmt3a
Dntt
Dock9
Dok2
Dpcd
Dph3
Dph5
Dpy19l1
Dqx1
Drc1
Drosha
Dtl
Dtx1
Dusp1
Dusp10
Dusp2
Dusp5
Dusp6
Dync1i2
Dynll1
Dynll2
Dynlt1c
Dzip1
E2f1
E2f2
Eaf1
Eci1
Ecm1
Edaradd
Edem1
Eef1b2
Eef2kmt
Egln3
Egr1
Egr2
Ehd1
Eid1
Eif2ak2
Scaled denoised expression
Pseudotime
Genotype
AND
OT−II
MHCI−/−
F5
OT−I
MHCII−/−

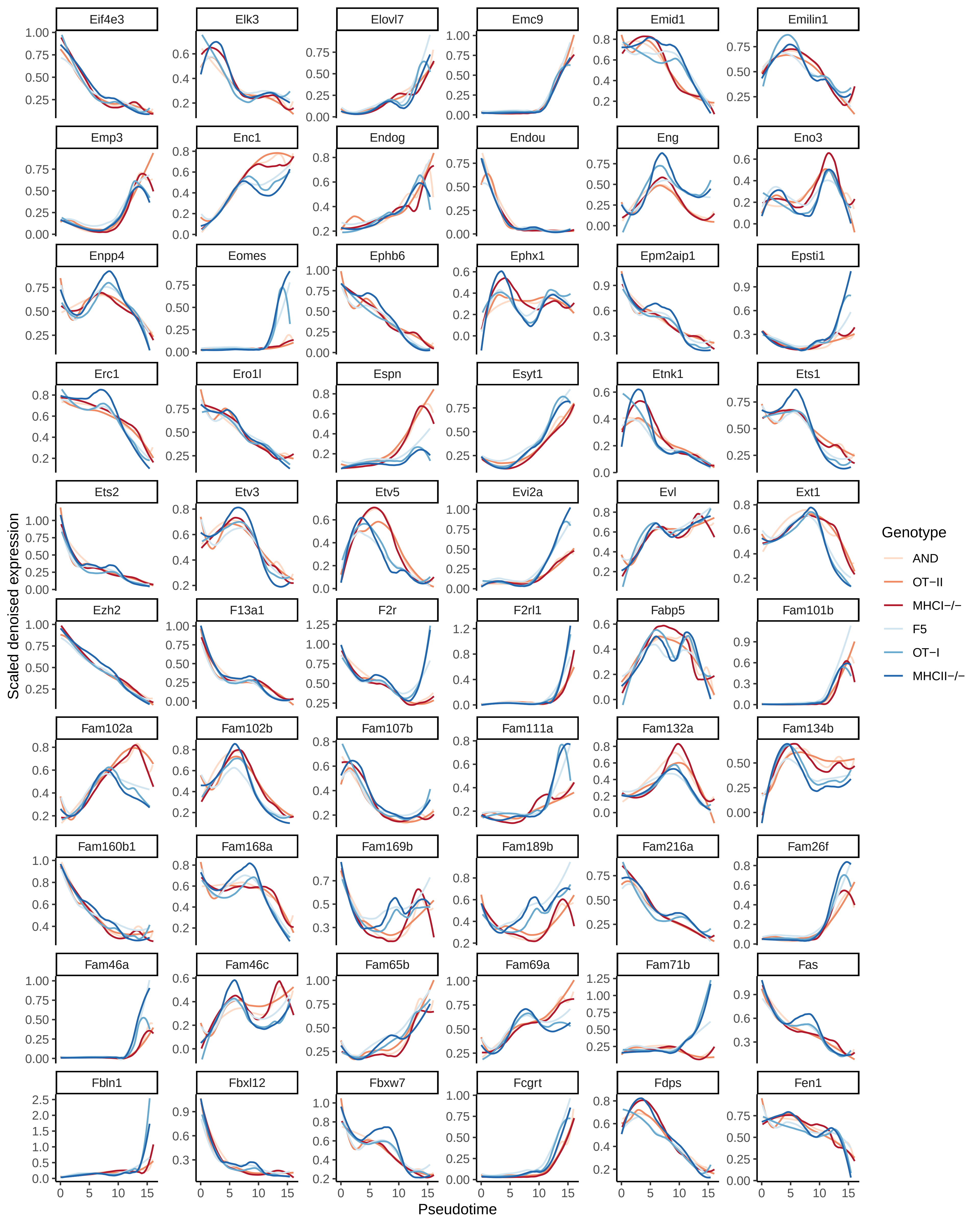
Eif4e3
Elk3
Elovl7
Emc9
Emid1
Emilin1
Emp3
Enc1
Endog
Endou
Eng
Eno3
Enpp4
Eomes
Ephb6
Ephx1
Epm2aip1
Epsti1
Erc1
Ero1l
Espn
Esyt1
Etnk1
Ets1
Ets2
Etv3
Etv5
Evi2a
Evl
Ext1
Ezh2
F13a1
F2r
F2rl1
Fabp5
Fam101b
Fam102a
Fam102b
Fam107b
Fam111a
Fam132a
Fam134b
Fam160b1
Fam168a
Fam169b
Fam189b
Fam216a
Fam26f
Fam46a
Fam46c
Fam65b
Fam69a
Fam71b
Fas
Fbln1
Fbxl12
Fbxw7
Fcgrt
Fdps
Fen1
Scaled denoised expression
Pseudotime
Genotype
AND
OT−II
MHCI−/−
F5
OT−I
MHCII−/−

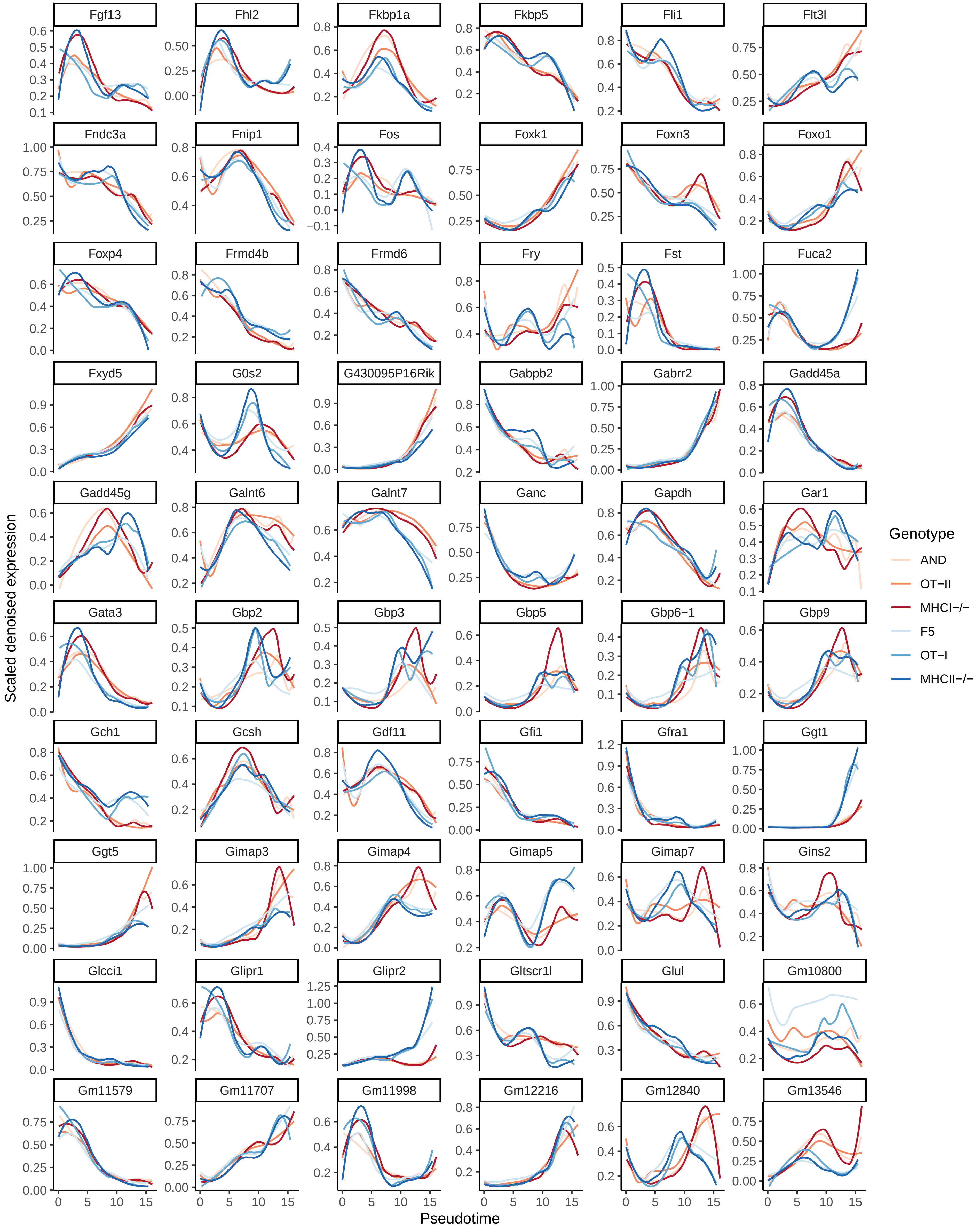

Fgf13
Fhl2
Fkbp1a
Fkbp5
Fli1
Flt3l
Fndc3a
Fnip1
Fos
Foxk1
Foxn3
Foxo1
Foxp4
Frmd4b
Frmd6
Fry
Fst
Fuca2
Fxyd5
G0s2
G430095P16Rik
Gabpb2
Gabrr2
Gadd45a
Gadd45g
Galnt6
Galnt7
Ganc
Gapdh
Gar1
Gata3
Gbp2
Gbp3
Gbp5
Gbp6−1
Gbp9
Gch1
Gcsh
Gdf11
Gfi1
Gfra1
Ggt1
Ggt5
Gimap3
Gimap4
Gimap5
Gimap7
Gins2
Glcci1
Glipr1
Glipr2
Gltscr1l
Glul
Gm10800
Gm11579
Gm11707
Gm11998
Gm12216
Gm12840
Gm13546
Scaled denoised expression
Pseudotime
Genotype
AND
OT−II
MHCI−/−
F5
OT−I
MHCII−/−

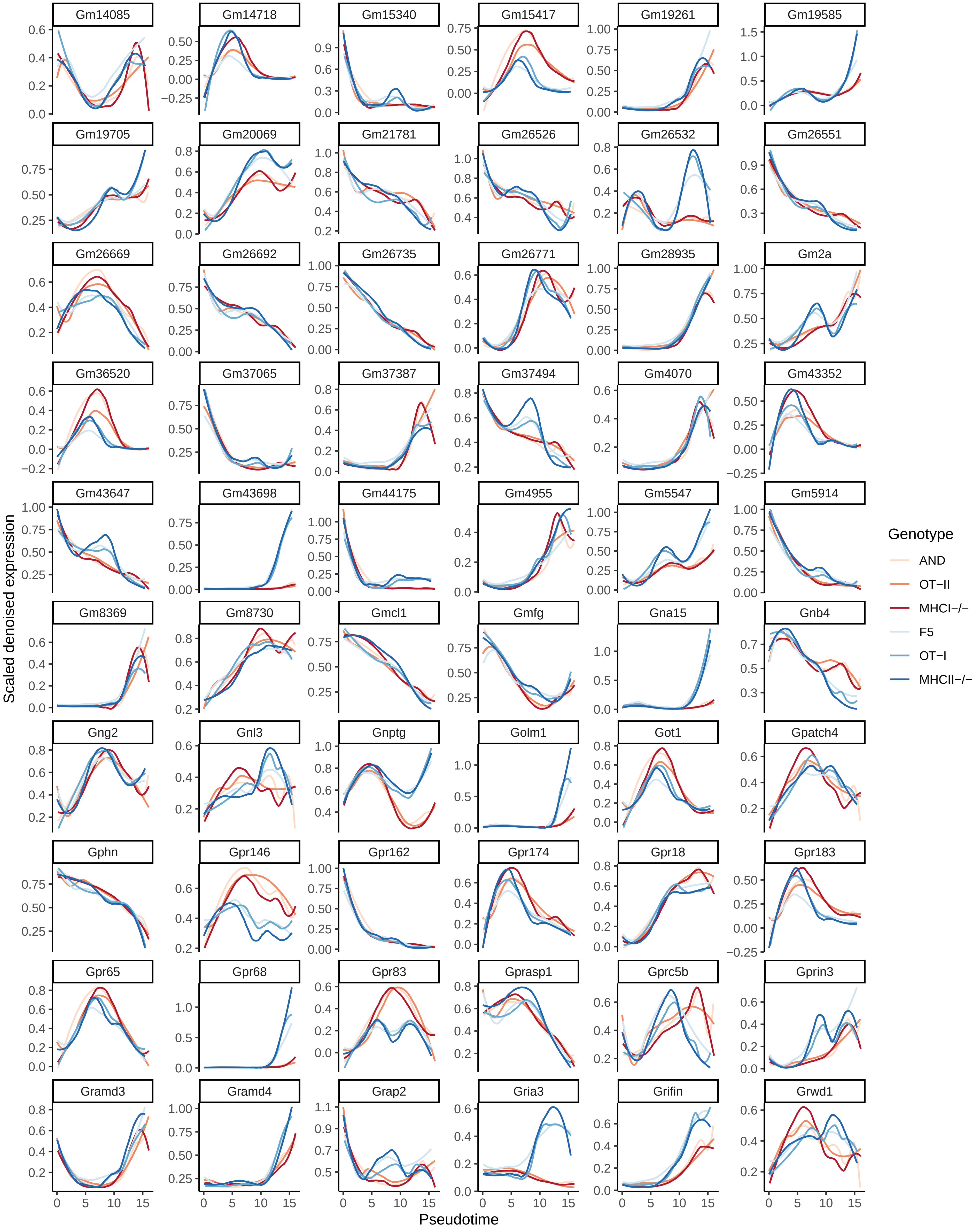
Gm14085
Gm14718
Gm15340
Gm15417
Gm19261
Gm19585
Gm19705
Gm20069
Gm21781
Gm26526
Gm26532
Gm26551
Gm26669
Gm26692
Gm26735
Gm26771
Gm28935
Gm2a
Gm36520
Gm37065
Gm37387
Gm37494
Gm4070
Gm43352
Gm43647
Gm43698
Gm44175
Gm4955
Gm5547
Gm5914
Gm8369
Gm8730
Gmcl1
Gmfg
Gna15
Gnb4
Gng2
Gnl3
Gnptg
Golm1
Got1
Gpatch4
Gphn
Gpr146
Gpr162
Gpr174
Gpr18
Gpr183
Gpr65
Gpr68
Gpr83
Gprasp1
Gprc5b
Gprin3
Gramd3
Gramd4
Grap2
Gria3
Grifin
Grwd1
Scaled denoised expression
Pseudotime
Genotype
AND
OT−II
MHCI−/−
F5
OT−I
MHCII−/−

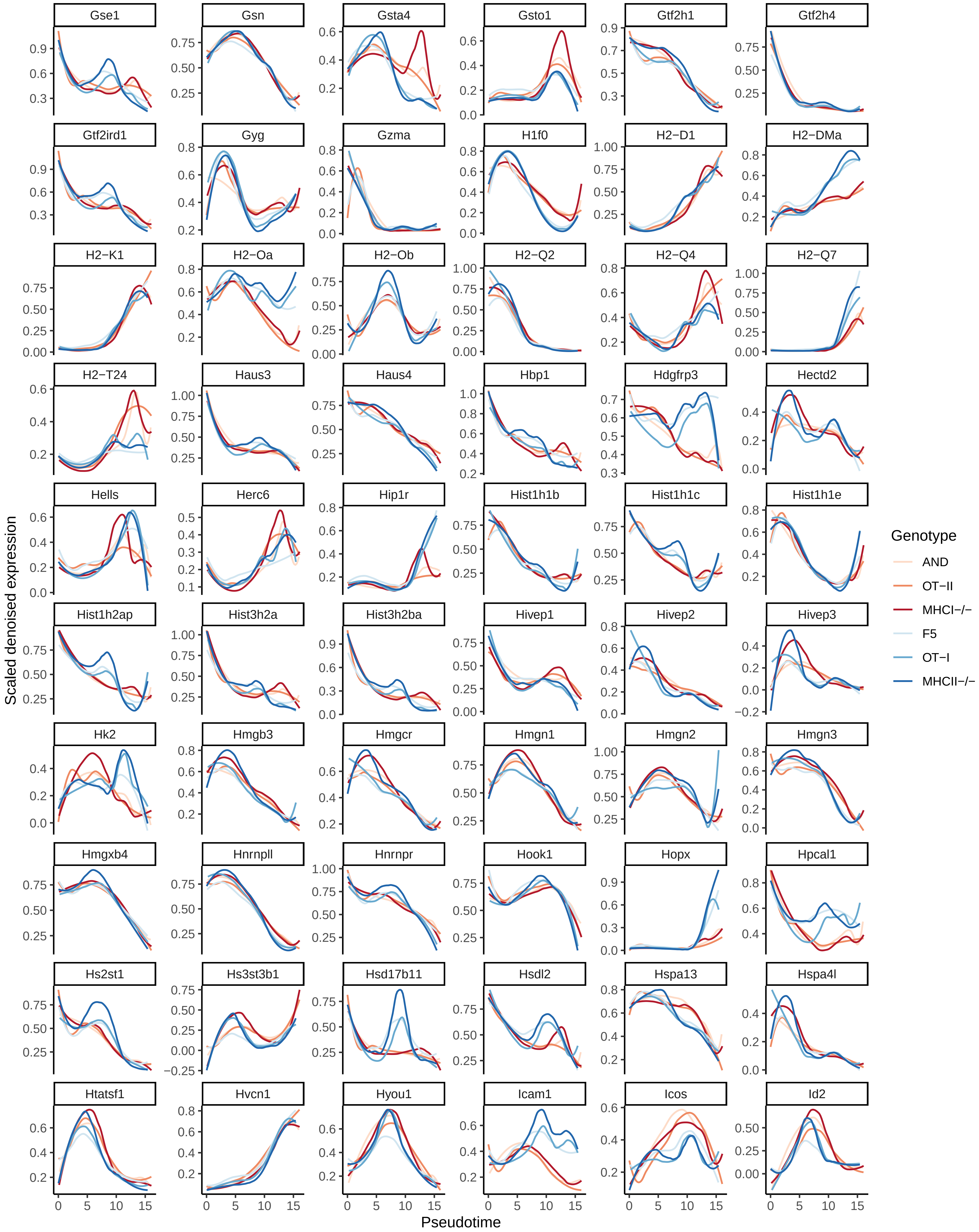

Gse1
Gsn
Gsta4
Gsto1
Gtf2h1
Gtf2h4
Gtf2ird1
Gyg
Gzma
H1f0
H2−D1
H2−DMa
H2−K1
H2−Oa
H2−Ob
H2−Q2
H2−Q4
H2−Q7
H2−T24
Haus3
Haus4
Hbp1
Hdgfrp3
Hectd2
Hells
Herc6
Hip1r
Hist1h1b
Hist1h1c
Hist1h1e
Hist1h2ap
Hist3h2a
Hist3h2ba
Hivep1
Hivep2
Hivep3
Hk2
Hmgb3
Hmgcr
Hmgn1
Hmgn2
Hmgn3
Hmgxb4
Hnrnpll
Hnrnpr
Hook1
Hopx
Hpcal1
Hs2st1
Hs3st3b1
Hsd17b11
Hsdl2
Hspa13
Hspa4l
Htatsf1
Hvcn1
Hyou1
Icam1
Icos
Id2
Scaled denoised expression
Pseudotime
Genotype
AND
OT−II
MHCI−/−
F5
OT−I
MHCII−/−

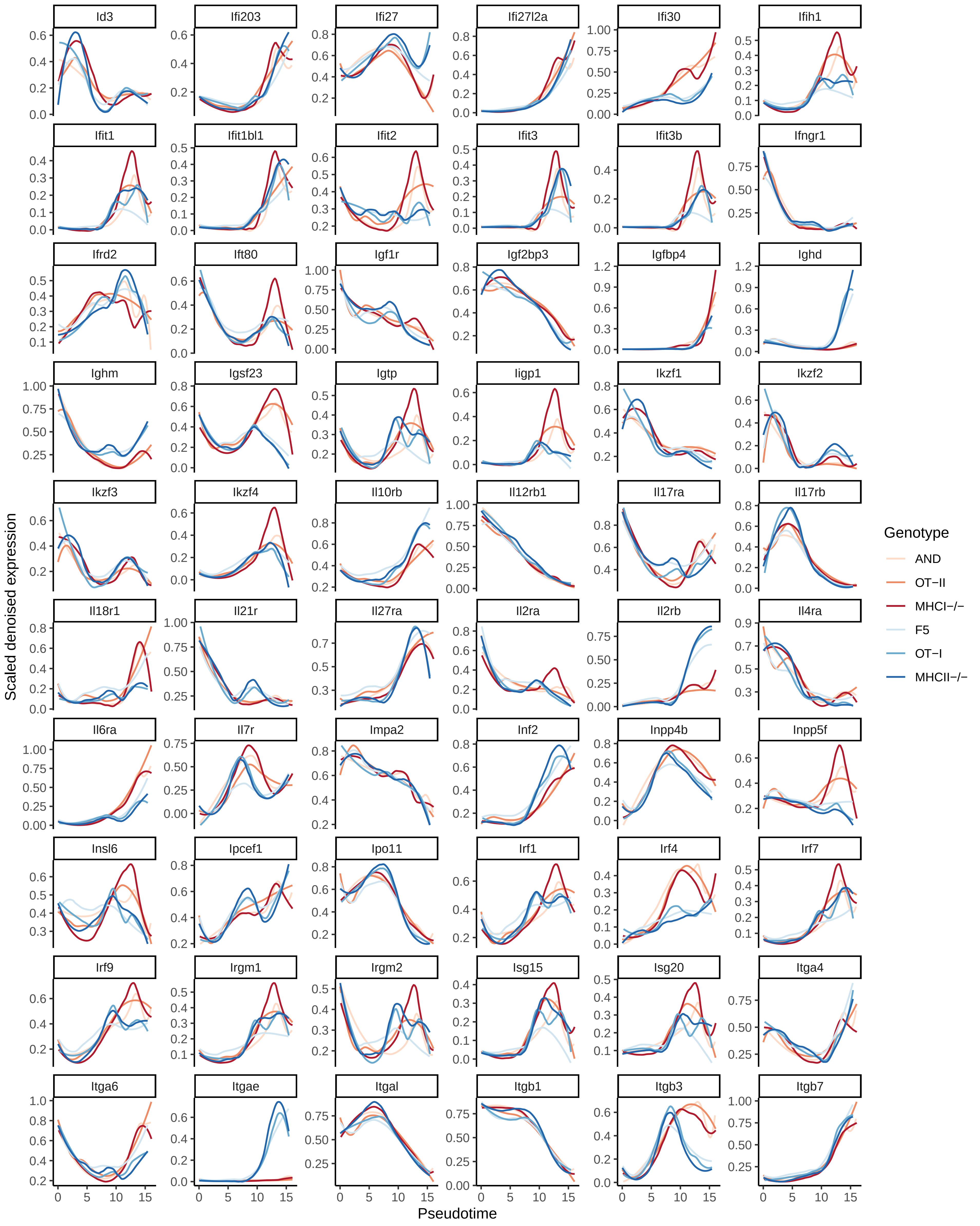
Id3
Ifi203
Ifi27
Ifi27l2a
Ifi30
Ifih1
Ifit1
Ifit1bl1
Ifit2
Ifit3
Ifit3b
Ifngr1
Ifrd2
Ift80
Igf1r
Igf2bp3
Igfbp4
Ighd
Ighm
Igsf23
Igtp
Iigp1
Ikzf1
Ikzf2
Ikzf3
Ikzf4
Il10rb
Il12rb1
Il17ra
Il17rb
Il18r1
Il21r
Il27ra
Il2ra
Il2rb
Il4ra
Il6ra
Il7r
Impa2
Inf2
Inpp4b
Inpp5f
Insl6
Ipcef1
Ipo11
Irf1
Irf4
Irf7
Irf9
Irgm1
Irgm2
Isg15
Isg20
Itga4
Itga6
Itgae
Itgal
Itgb1
Itgb3
Itgb7
Scaled denoised expression
Pseudotime
Genotype
AND
OT−II
MHCI−/−
F5
OT−I
MHCII−/−

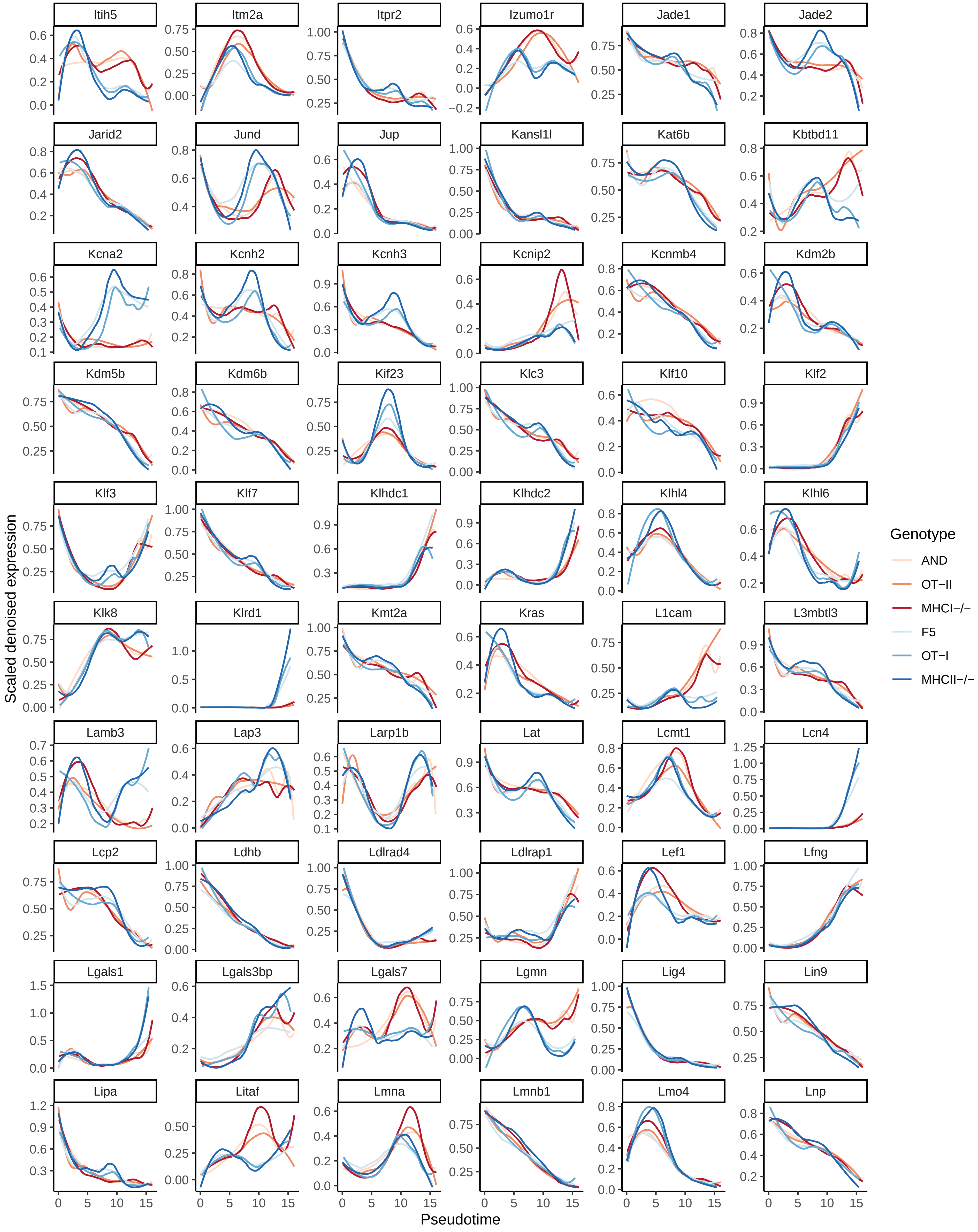
Itih5
Itm2a
Itpr2
Izumo1r
Jade1
Jade2
Jarid2
Jund
Jup
Kansl1l
Kat6b
Kbtbd11
Kcna2
Kcnh2
Kcnh3
Kcnip2
Kcnmb4
Kdm2b
Kdm5b
Kdm6b
Kif23
Klc3
Klf10
Klf2
Klf3
Klf7
Klhdc1
Klhdc2
Klhl4
Klhl6
Klk8
Klrd1
Kmt2a
Kras
L1cam
L3mbtl3
Lamb3
Lap3
Larp1b
Lat
Lcmt1
Lcn4
Lcp2
Ldhb
Ldlrad4
Ldlrap1
Lef1
Lfng
Lgals1
Lgals3bp
Lgals7
Lgmn
Lig4
Lin9
Lipa
Litaf
Lmna
Lmnb1
Lmo4
Lnp
Scaled denoised expression
Pseudotime
Genotype
AND
OT−II
MHCI−/−
F5
OT−I
MHCII−/−

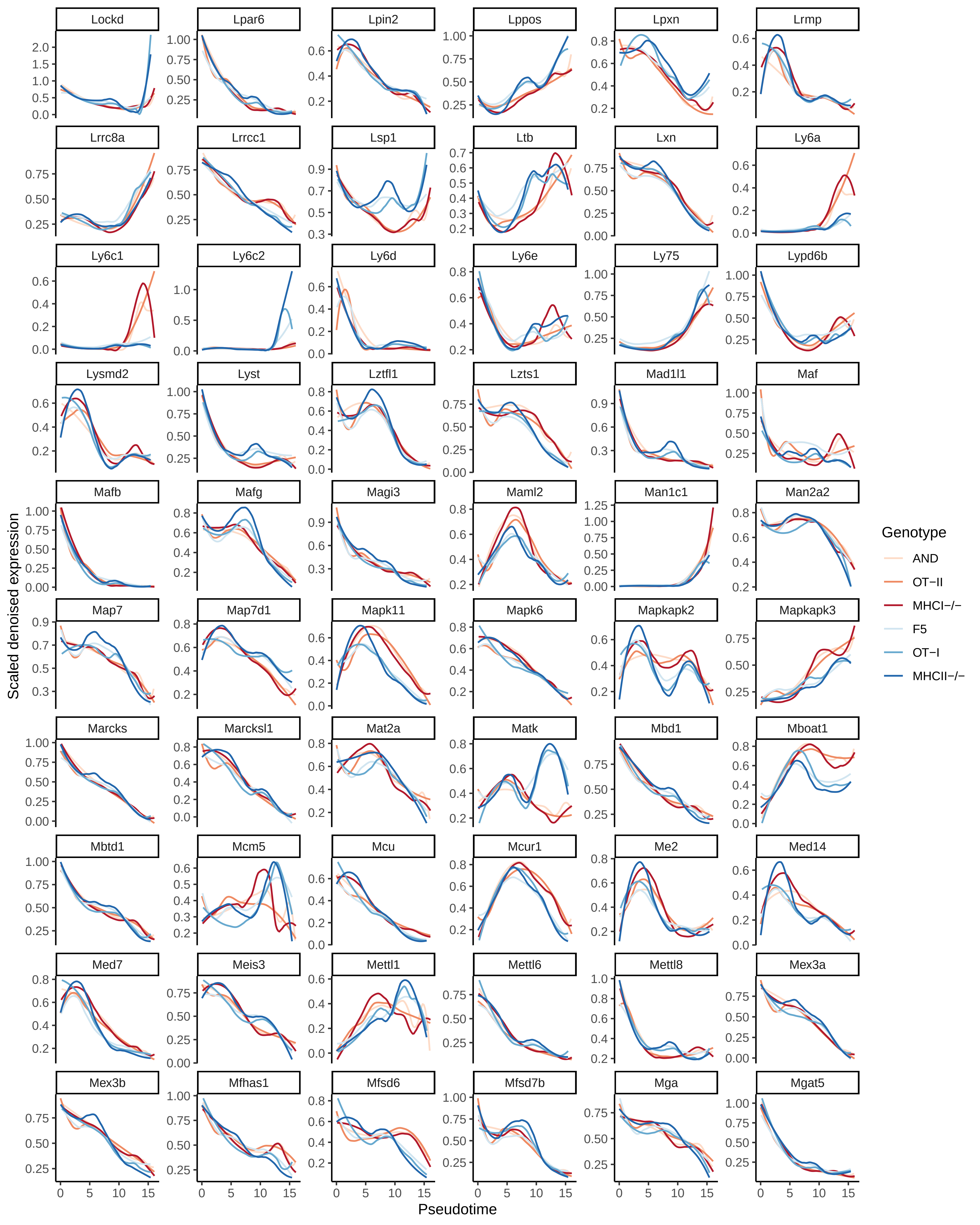

Lockd
Lpar6
Lpin2
Lppos
Lpxn
Lrmp
Lrrc8a
Lrrcc1
Lsp1
Ltb
Lxn
Ly6a
Ly6c1
Ly6c2
Ly6d
Ly6e
Ly75
Lypd6b
Lysmd2
Lyst
Lztfl1
Lzts1
Mad1l1
Maf
Mafb
Mafg
Magi3
Maml2
Man1c1
Man2a2
Map7
Map7d1
Mapk11
Mapk6
Mapkapk2
Mapkapk3
Marcks
Marcksl1
Mat2a
Matk
Mbd1
Mboat1
Mbtd1
Mcm5
Mcu
Mcur1
Me2
Med14
Med7
Meis3
Mettl1
Mettl6
Mettl8
Mex3a
Mex3b
Mfhas1
Mfsd6
Mfsd7b
Mga
Mgat5
Scaled denoised expression
Pseudotime
Genotype
AND
OT−II
MHCI−/−
F5
OT−I
MHCII−/−

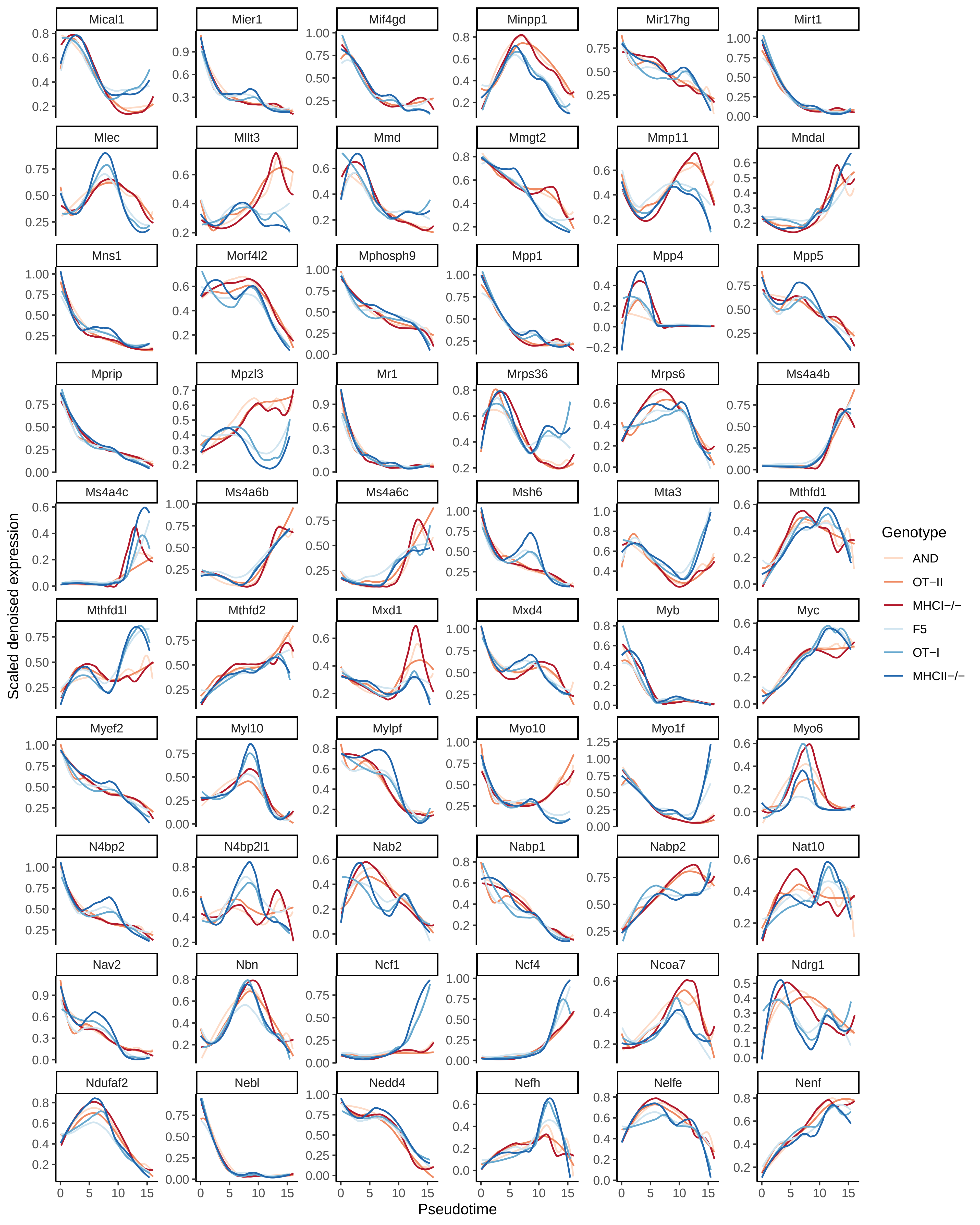
Mical1
Mier1
Mif4gd
Minpp1
Mir17hg
Mirt1
Mlec
Mllt3
Mmd
Mmgt2
Mmp11
Mndal
Mns1
Morf4l2
Mphosph9
Mpp1
Mpp4
Mpp5
Mprip
Mpzl3
Mr1
Mrps36
Mrps6
Ms4a4b
Ms4a4c
Ms4a6b
Ms4a6c
Msh6
Mta3
Mthfd1
Mthfd1l
Mthfd2
Mxd1
Mxd4
Myb
Myc
Myef2
Myl10
Mylpf
Myo10
Myo1f
Myo6
N4bp2
N4bp2l1
Nab2
Nabp1
Nabp2
Nat10
Nav2
Nbn
Ncf1
Ncf4
Ncoa7
Ndrg1
Ndufaf2
Nebl
Nedd4
Nefh
Nelfe
Nenf
Scaled denoised expression
Pseudotime
Genotype
AND
OT−II
MHCI−/−
F5
OT−I
MHCII−/−

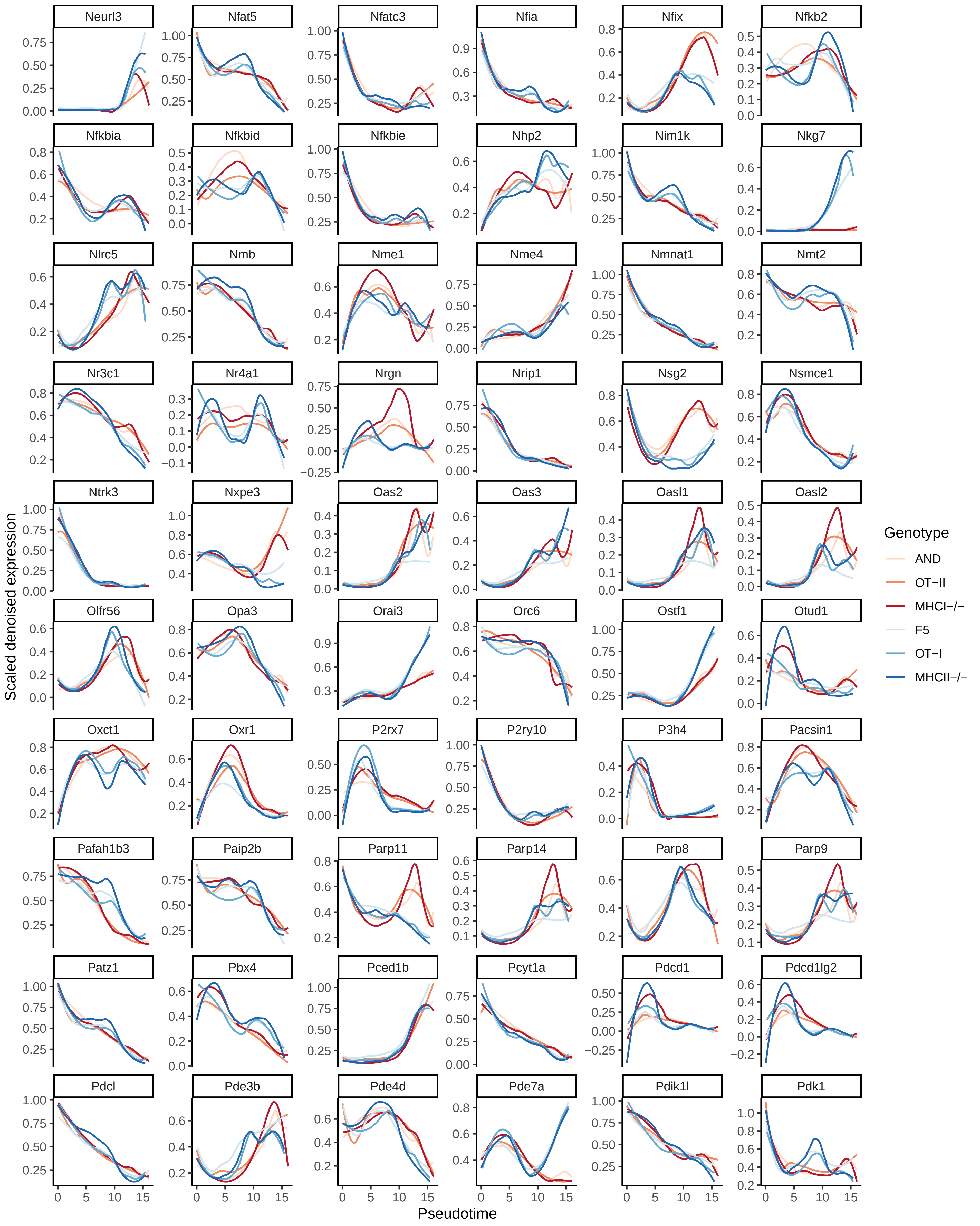
Neurl3
Nfat5
Nfatc3
Nfia
Nfix
Nfkb2
Nfkbia
Nfkbid
Nfkbie
Nhp2
Nim1k
Nkg7
Nlrc5
Nmb
Nme1
Nme4
Nmnat1
Nmt2
Nr3c1
Nr4a1
Nrgn
Nrip1
Nsg2
Nsmce1
Ntrk3
Nxpe3
Oas2
Oas3
Oasl1
Oasl2
Olfr56
Opa3
Orai3
Orc6
Ostf1
Otud1
Oxct1
Oxr1
P2rx7
P2ry10
P3h4
Pacsin1
Pafah1b3
Paip2b
Parp11
Parp14
Parp8
Parp9
Patz1
Pbx4
Pced1b
Pcyt1a
Pdcd1
Pdcd1lg2
Pdcl
Pde3b
Pde4d
Pde7a
Pdik1l
Pdk1
Scaled denoised expression
Pseudotime
Genotype
AND
OT−II
MHCI−/−
F5
OT−I
MHCII−/−

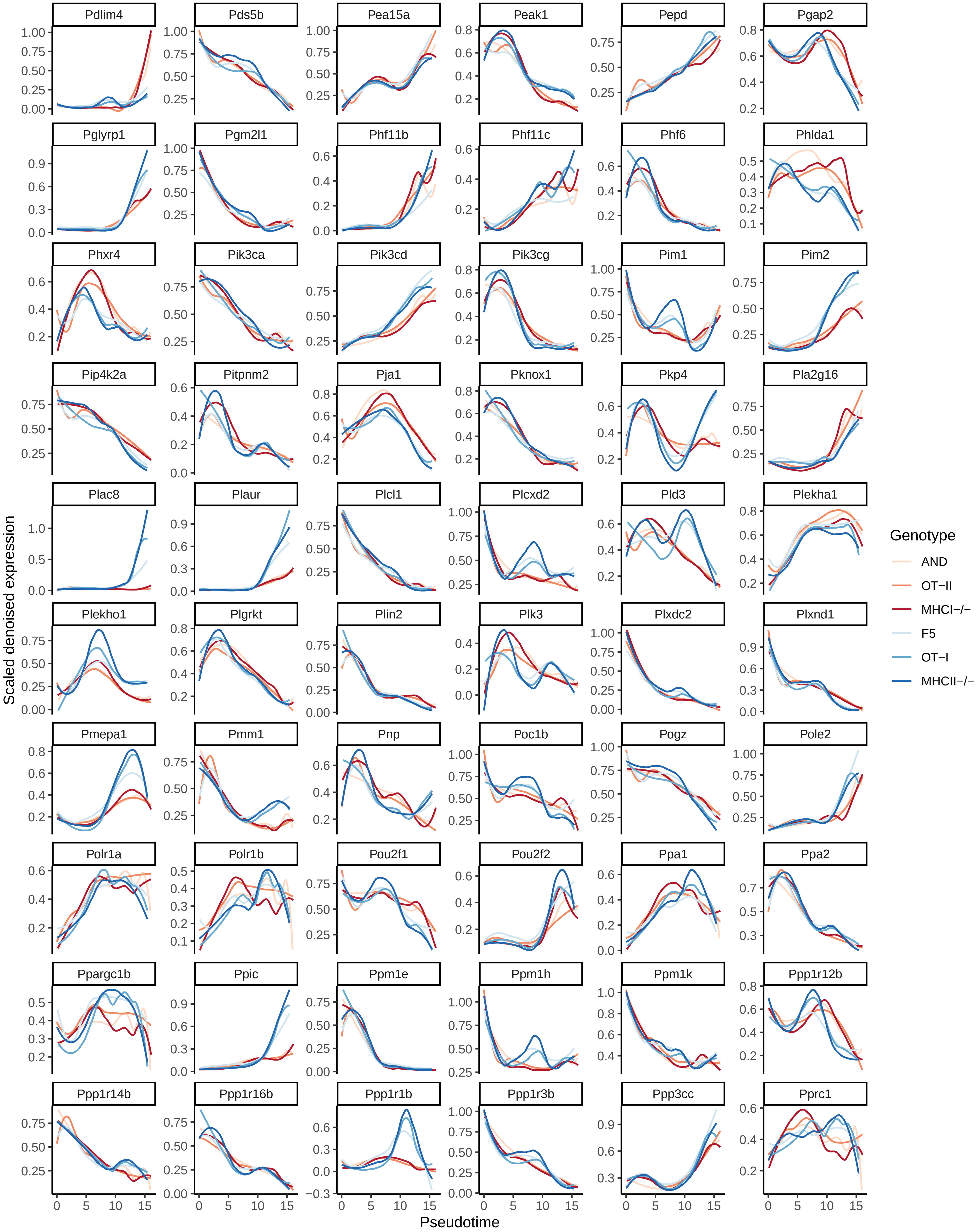

Pdlim4
Pds5b
Pea15a
Peak1
Pepd
Pgap2
Pglyrp1
Pgm2l1
Phf11b
Phf11c
Phf6
Phlda1
Phxr4
Pik3ca
Pik3cd
Pik3cg
Pim1
Pim2
Pip4k2a
Pitpnm2
Pja1
Pknox1
Pkp4
Pla2g16
Plac8
Plaur
Plcl1
Plcxd2
Pld3
Plekha1
Plekho1
Plgrkt
Plin2
Plk3
Plxdc2
Plxnd1
Pmepa1
Pmm1
Pnp
Poc1b
Pogz
Pole2
Polr1a
Polr1b
Pou2f1
Pou2f2
Ppa1
Ppa2
Ppargc1b
Ppic
Ppm1e
Ppm1h
Ppm1k
Ppp1r12b
Ppp1r14b
Ppp1r16b
Ppp1r1b
Ppp1r3b
Ppp3cc
Pprc1
Scaled denoised expression
Pseudotime
Genotype
AND
OT−II
MHCI−/−
F5
OT−I
MHCII−/−

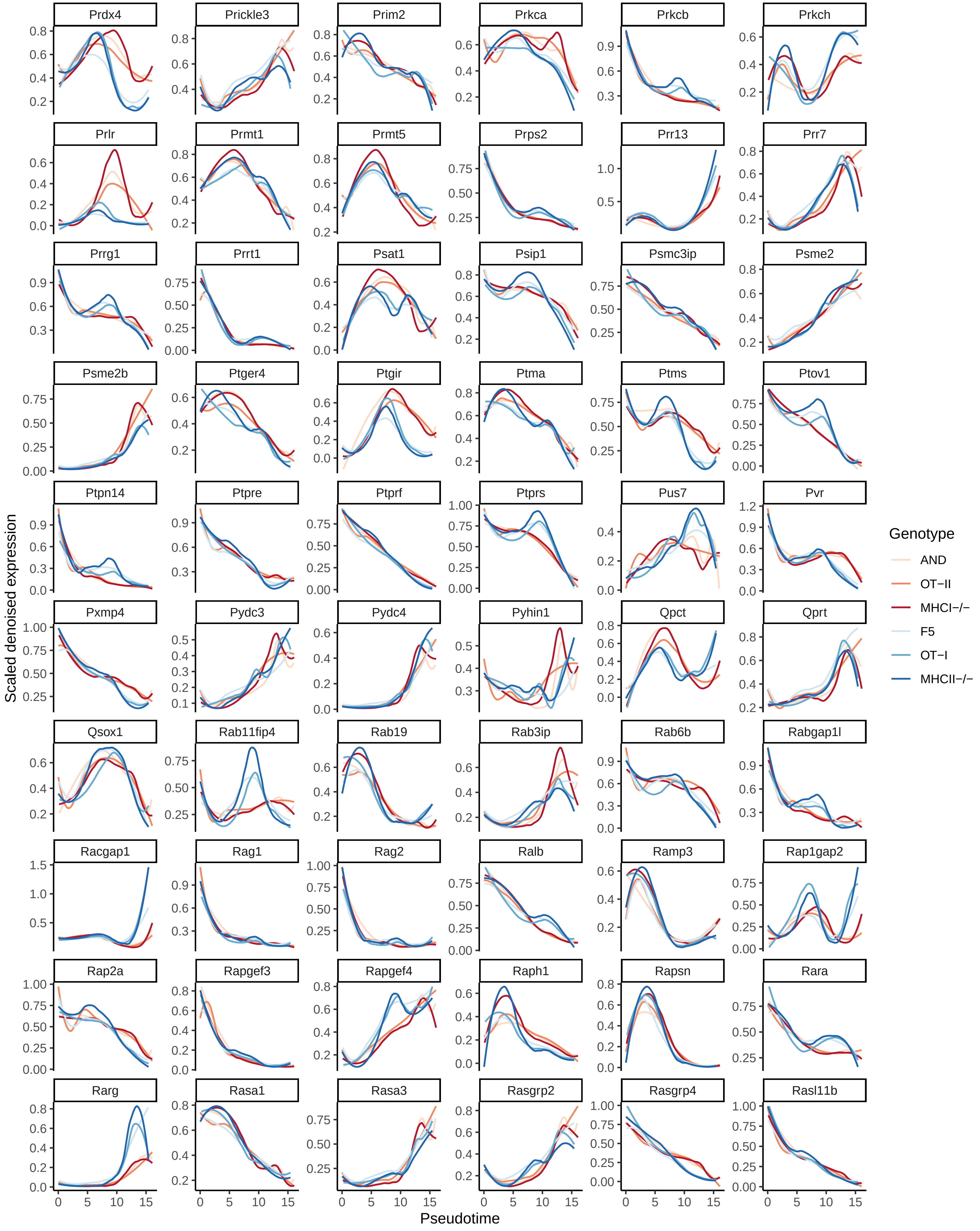

Prdx4
Prickle3
Prim2
Prkca
Prkcb
Prkch
Prlr
Prmt1
Prmt5
Prps2
Prr13
Prr7
Prrg1
Prrt1
Psat1
Psip1
Psmc3ip
Psme2
Psme2b
Ptger4
Ptgir
Ptma
Ptms
Ptov1
Ptpn14
Ptpre
Ptprf
Ptprs
Pus7
Pvr
Pxmp4
Pydc3
Pydc4
Pyhin1
Qpct
Qprt
Qsox1
Rab11fip4
Rab19
Rab3ip
Rab6b
Rabgap1l
Racgap1
Rag1
Rag2
Ralb
Ramp3
Rap1gap2
Rap2a
Rapgef3
Rapgef4
Raph1
Rapsn
Rara
Rarg
Rasa1
Rasa3
Rasgrp2
Rasgrp4
Rasl11b
Scaled denoised expression
Pseudotime
Genotype
AND
OT−II
MHCI−/−
F5
OT−I
MHCII−/−

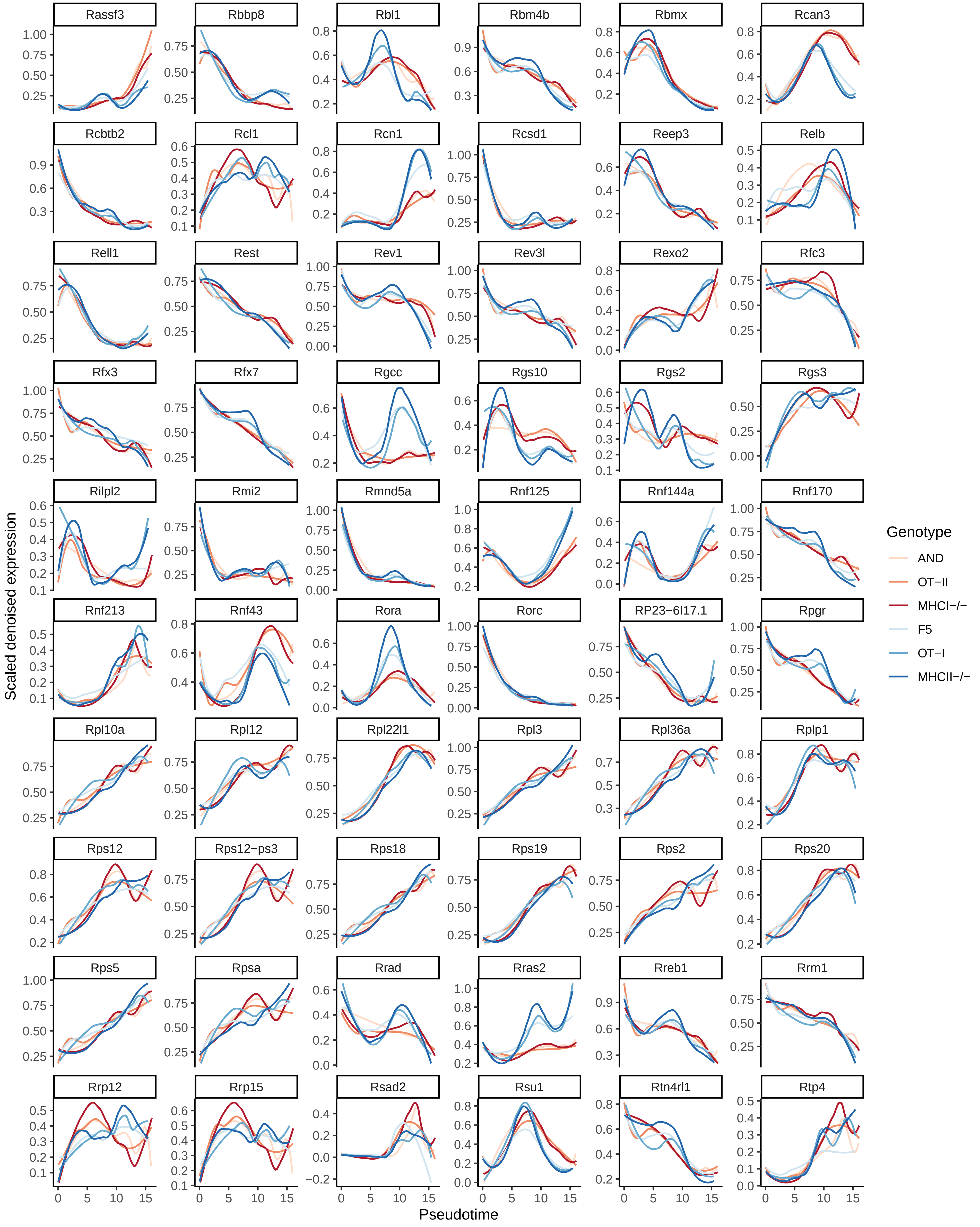

Rassf3
Rbbp8
Rbl1
Rbm4b
Rbmx
Rcan3
Rcbtb2
Rcl1
Rcn1
Rcsd1
Reep3
Relb
Rell1
Rest
Rev1
Rev3l
Rexo2
Rfc3
Rfx3
Rfx7
Rgcc
Rgs10
Rgs2
Rgs3
Rilpl2
Rmi2
Rmnd5a
Rnf125
Rnf144a
Rnf170
Rnf213
Rnf43
Rora
Rorc
RP23−6I17.1
Rpgr
Rpl10a
Rpl12
Rpl22l1
Rpl3
Rpl36a
Rplp1
Rps12
Rps12−ps3
Rps18
Rps19
Rps2
Rps20
Rps5
Rpsa
Rrad
Rras2
Rreb1
Rrm1
Rrp12
Rrp15
Rsad2
Rsu1
Rtn4rl1
Rtp4
Scaled denoised expression
Pseudotime
Genotype
AND
OT−II
MHCI−/−
F5
OT−I
MHCII−/−

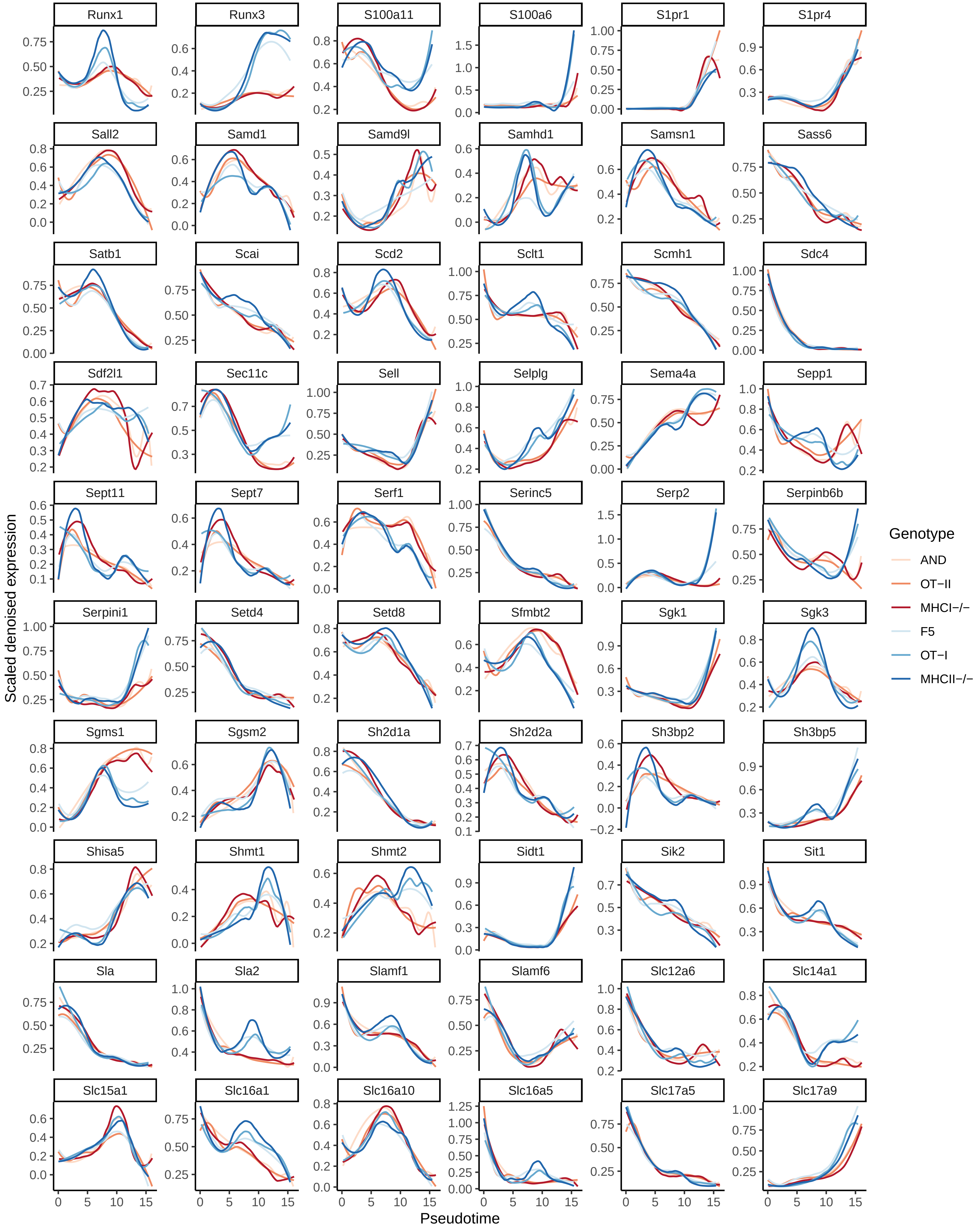
Runx1
Runx3
S100a11
S100a6
S1pr1
S1pr4
Sall2
Samd1
Samd9l
Samhd1
Samsn1
Sass6
Satb1
Scai
Scd2
Sclt1
Scmh1
Sdc4
Sdf2l1
Sec11c
Sell
Selplg
Sema4a
Sepp1
Sept11
Sept7
Serf1
Serinc5
Serp2
Serpinb6b
Serpini1
Setd4
Setd8
Sfmbt2
Sgk1
Sgk3
Sgms1
Sgsm2
Sh2d1a
Sh2d2a
Sh3bp2
Sh3bp5
Shisa5
Shmt1
Shmt2
Sidt1
Sik2
Sit1
Sla
Sla2
Slamf1
Slamf6
Slc12a6
Slc14a1
Slc15a1
Slc16a1
Slc16a10
Slc16a5
Slc17a5
Slc17a9
Scaled denoised expression
Pseudotime
Genotype
AND
OT−II
MHCI−/−
F5
OT−I
MHCII−/−

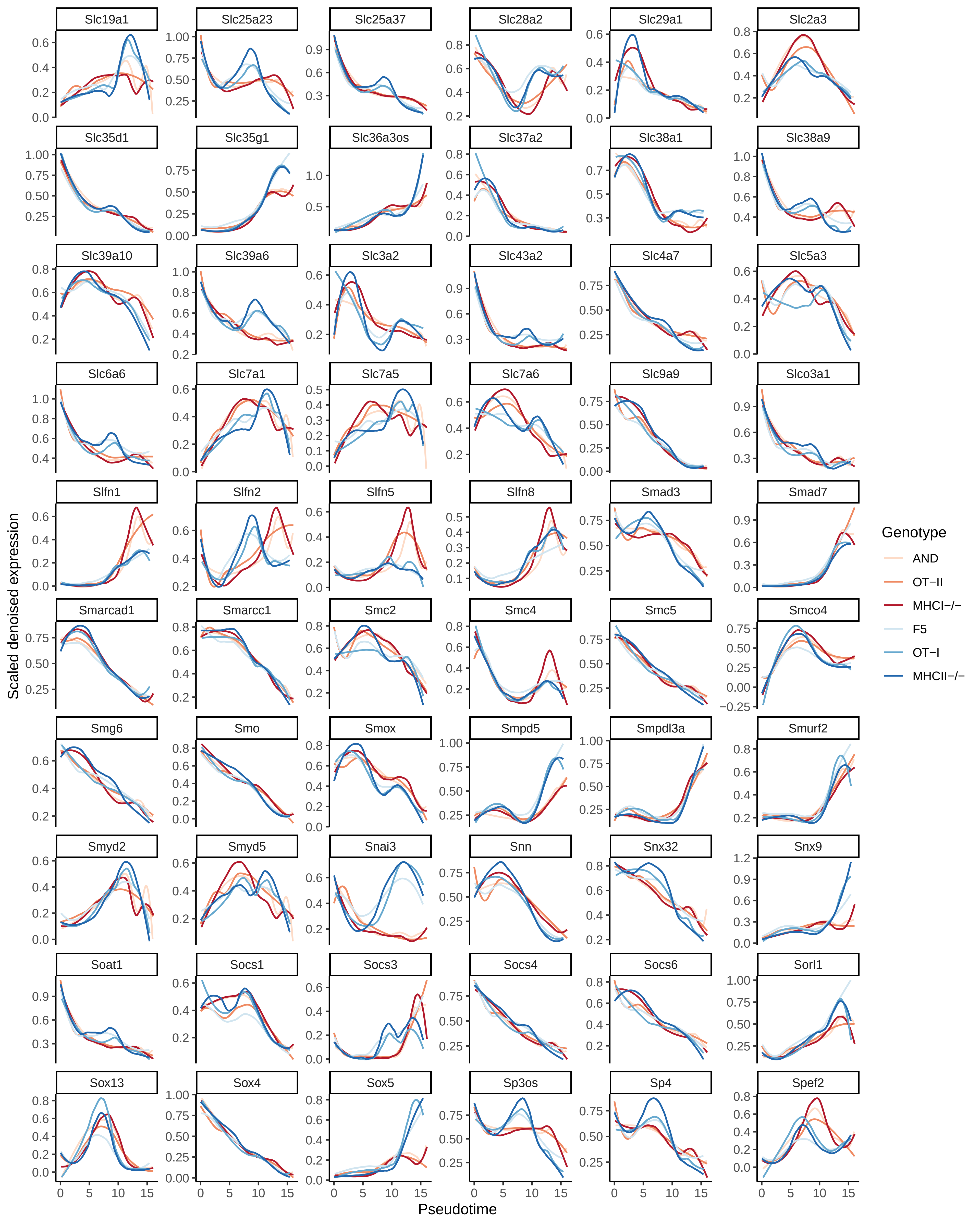

Slc19a1
Slc25a23
Slc25a37
Slc28a2
Slc29a1
Slc2a3
Slc35d1
Slc35g1
Slc36a3os
Slc37a2
Slc38a1
Slc38a9
Slc39a10
Slc39a6
Slc3a2
Slc43a2
Slc4a7
Slc5a3
Slc6a6
Slc7a1
Slc7a5
Slc7a6
Slc9a9
Slco3a1
Slfn1
Slfn2
Slfn5
Slfn8
Smad3
Smad7
Smarcad1
Smarcc1
Smc2
Smc4
Smc5
Smco4
Smg6
Smo
Smox
Smpd5
Smpdl3a
Smurf2
Smyd2
Smyd5
Snai3
Snn
Snx32
Snx9
Soat1
Socs1
Socs3
Socs4
Socs6
Sorl1
Sox13
Sox4
Sox5
Sp3os
Sp4
Spef2
Scaled denoised expression
Pseudotime
Genotype
AND
OT−II
MHCI−/−
F5
OT−I
MHCII−/−

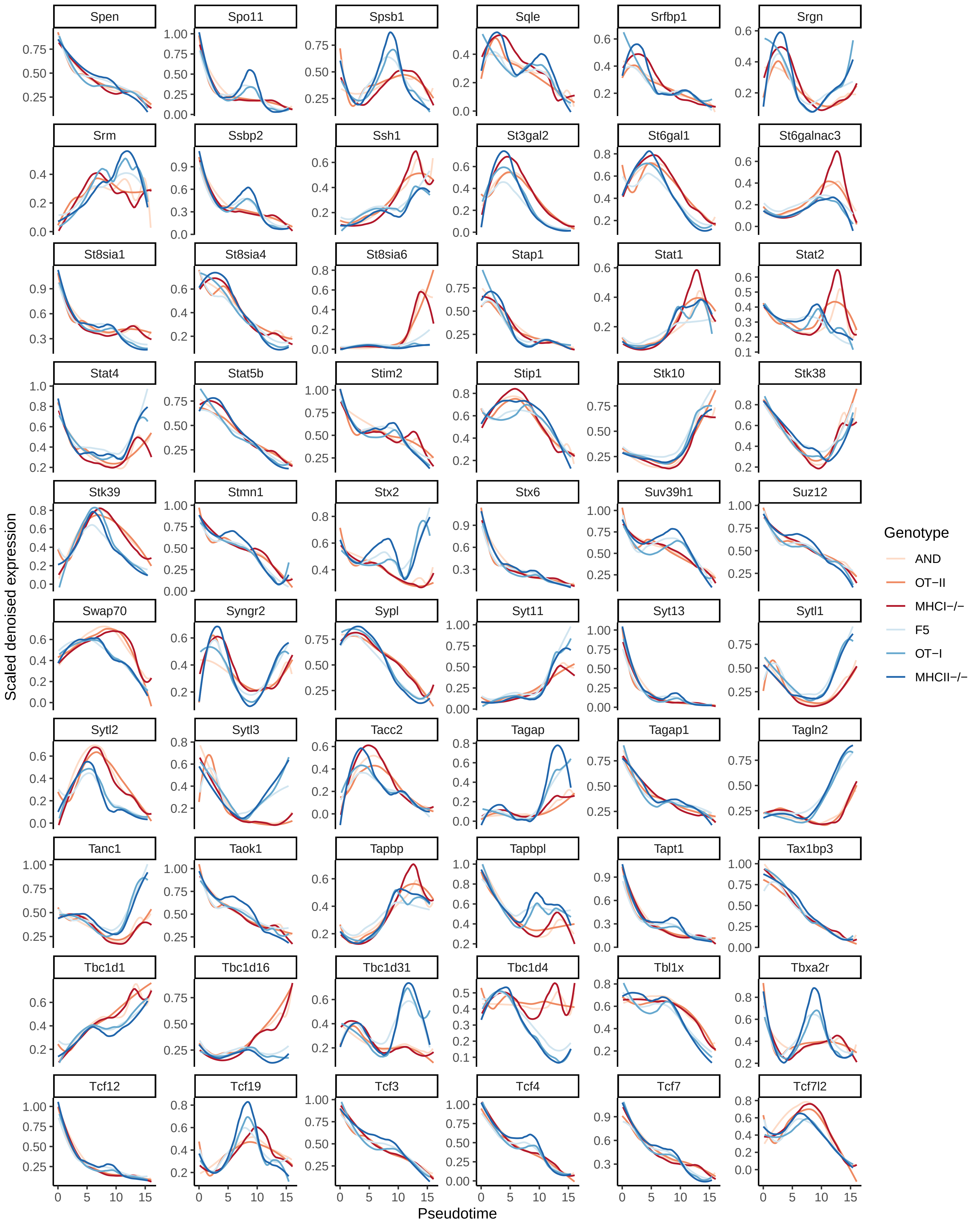

Spen
Spo11
Spsb1
Sqle
Srfbp1
Srgn
Srm
Ssbp2
Ssh1
St3gal2
St6gal1
St6galnac3
St8sia1
St8sia4
St8sia6
Stap1
Stat1
Stat2
Stat4
Stat5b
Stim2
Stip1
Stk10
Stk38
Stk39
Stmn1
Stx2
Stx6
Suv39h1
Suz12
Swap70
Syngr2
Sypl
Syt11
Syt13
Sytl1
Sytl2
Sytl3
Tacc2
Tagap
Tagap1
Tagln2
Tanc1
Taok1
Tapbp
Tapbpl
Tapt1
Tax1bp3
Tbc1d1
Tbc1d16
Tbc1d31
Tbc1d4
Tbl1x
Tbxa2r
Tcf12
Tcf19
Tcf3
Tcf4
Tcf7
Tcf7l2
Scaled denoised expression
Pseudotime
Genotype
AND
OT−II
MHCI−/−
F5
OT−I
MHCII−/−

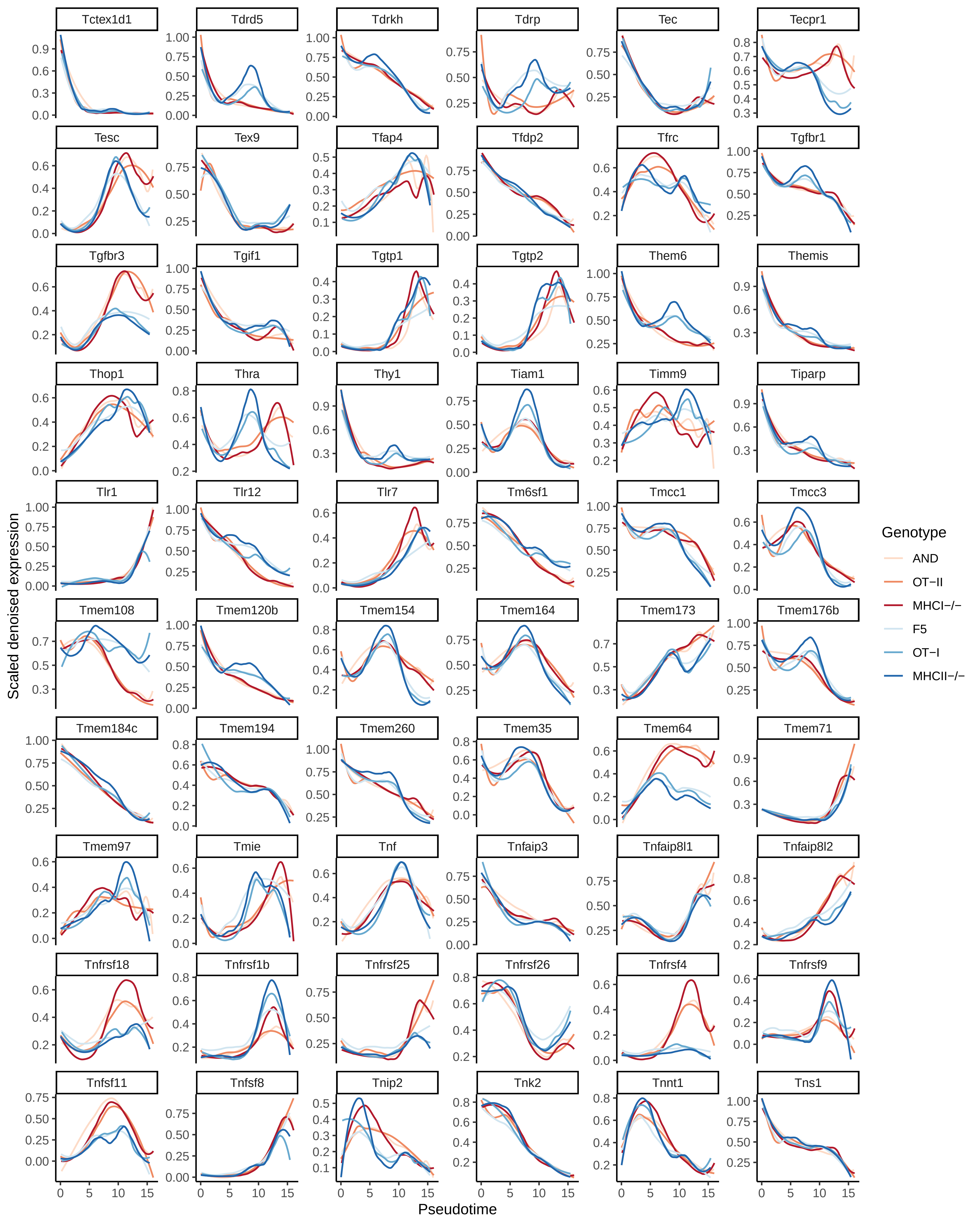

Tctex1d1
Tdrd5
Tdrkh
Tdrp
Tec
Tecpr1
Tesc
Tex9
Tfap4
Tfdp2
Tfrc
Tgfbr1
Tgfbr3
Tgif1
Tgtp1
Tgtp2
Them6
Themis
Thop1
Thra
Thy1
Tiam1
Timm9
Tiparp
Tlr1
Tlr12
Tlr7
Tm6sf1
Tmcc1
Tmcc3
Tmem108
Tmem120b
Tmem154
Tmem164
Tmem173
Tmem176b
Tmem184c
Tmem194
Tmem260
Tmem35
Tmem64
Tmem71
Tmem97
Tmie
Tnf
Tnfaip3
Tnfaip8l1
Tnfaip8l2
Tnfrsf18
Tnfrsf1b
Tnfrsf25
Tnfrsf26
Tnfrsf4
Tnfrsf9
Tnfsf11
Tnfsf8
Tnip2
Tnk2
Tnnt1
Tns1
Scaled denoised expression
Pseudotime
Genotype
AND
OT−II
MHCI−/−
F5
OT−I
MHCII−/−

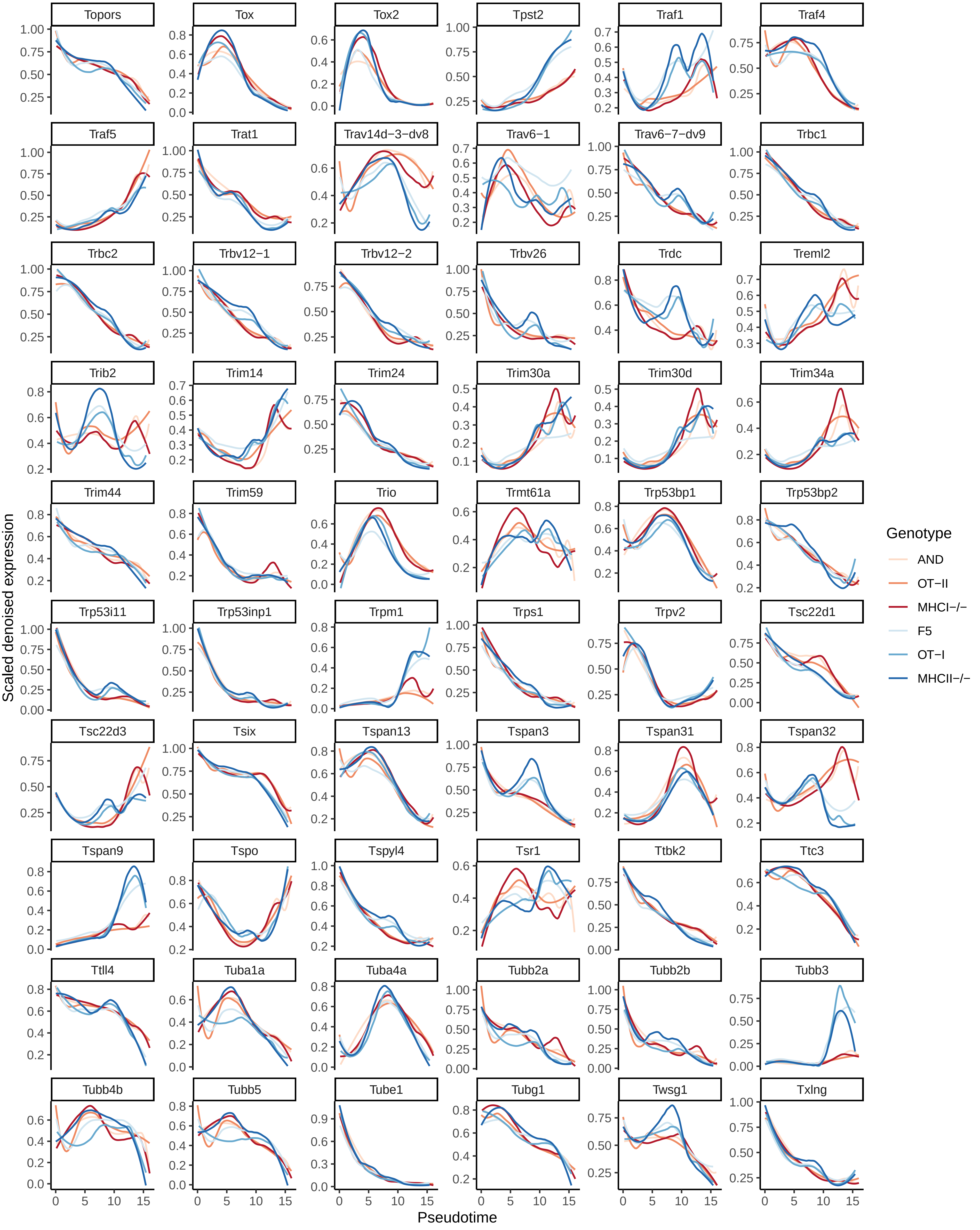
Topors
Tox
Tox2
Tpst2
Traf1
Traf4
Traf5
Trat1
Trav14d−3−dv8
Trav6−1
Trav6−7−dv9
Trbc1
Trbc2
Trbv12−1
Trbv12−2
Trbv26
Trdc
Treml2
Trib2
Trim14
Trim24
Trim30a
Trim30d
Trim34a
Trim44
Trim59
Trio
Trmt61a
Trp53bp1
Trp53bp2
Trp53i11
Trp53inp1
Trpm1
Trps1
Trpv2
Tsc22d1
Tsc22d3
Tsix
Tspan13
Tspan3
Tspan31
Tspan32
Tspan9
Tspo
Tspyl4
Tsr1
Ttbk2
Ttc3
Ttll4
Tuba1a
Tuba4a
Tubb2a
Tubb2b
Tubb3
Tubb4b
Tubb5
Tube1
Tubg1
Twsg1
Txlng
Scaled denoised expression
Pseudotime
Genotype
AND
OT−II
MHCI−/−
F5
OT−I
MHCII−/−

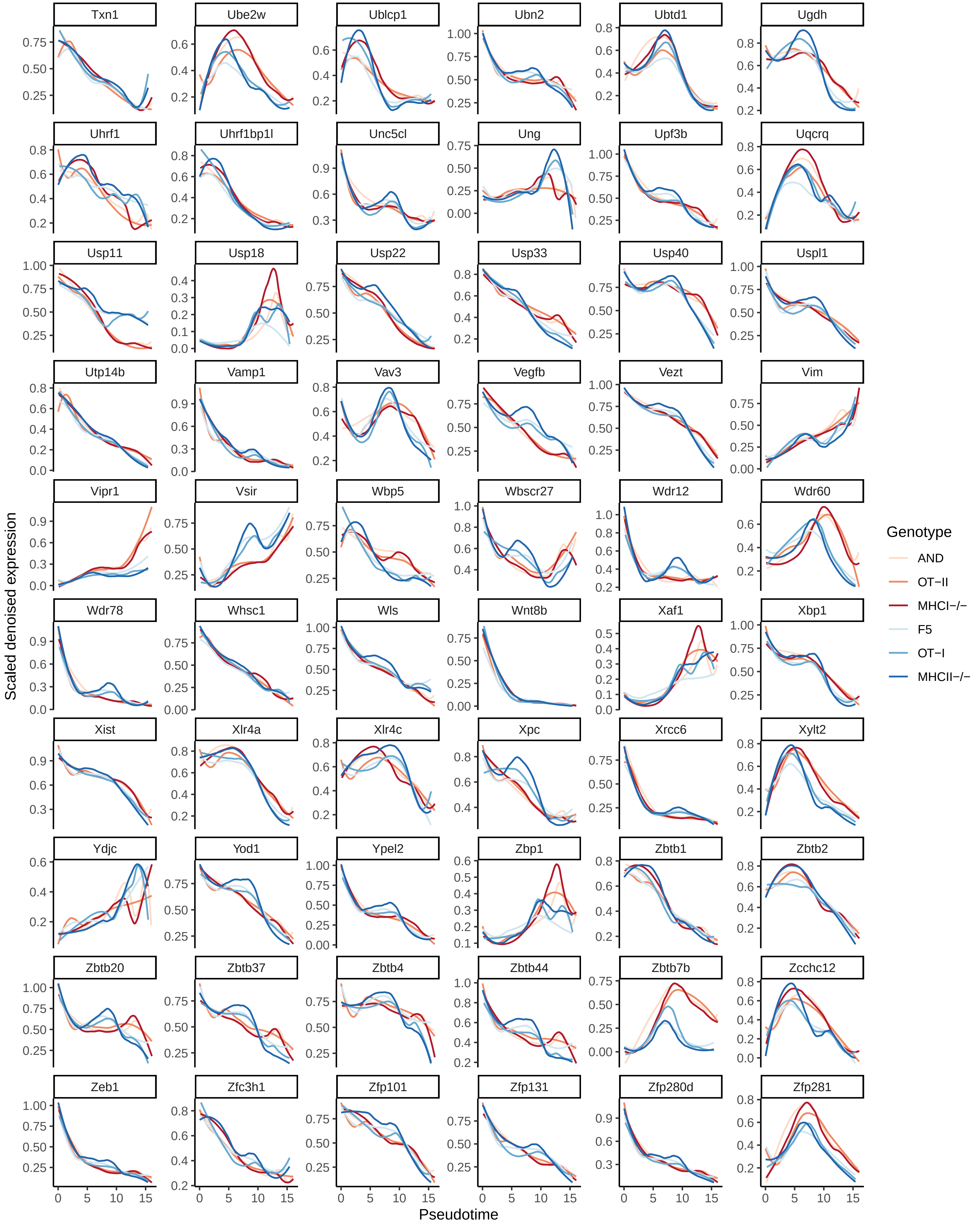
Txn1
Ube2w
Ublcp1
Ubn2
Ubtd1
Ugdh
Uhrf1
Uhrf1bp1l
Unc5cl
Ung
Upf3b
Uqcrq
Usp11
Usp18
Usp22
Usp33
Usp40
Uspl1
Utp14b
Vamp1
Vav3
Vegfb
Vezt
Vim
Vipr1
Vsir
Wbp5
Wbscr27
Wdr12
Wdr60
Wdr78
Whsc1
Wls
Wnt8b
Xaf1
Xbp1
Xist
Xlr4a
Xlr4c
Xpc
Xrcc6
Xylt2
Ydjc
Yod1
Ypel2
Zbp1
Zbtb1
Zbtb2
Zbtb20
Zbtb37
Zbtb4
Zbtb44
Zbtb7b
Zcchc12
Zeb1
Zfc3h1
Zfp101
Zfp131
Zfp280d
Zfp281
Scaled denoised expression
Pseudotime
Genotype
AND
OT−II
MHCI−/−
F5
OT−I
MHCII−/−

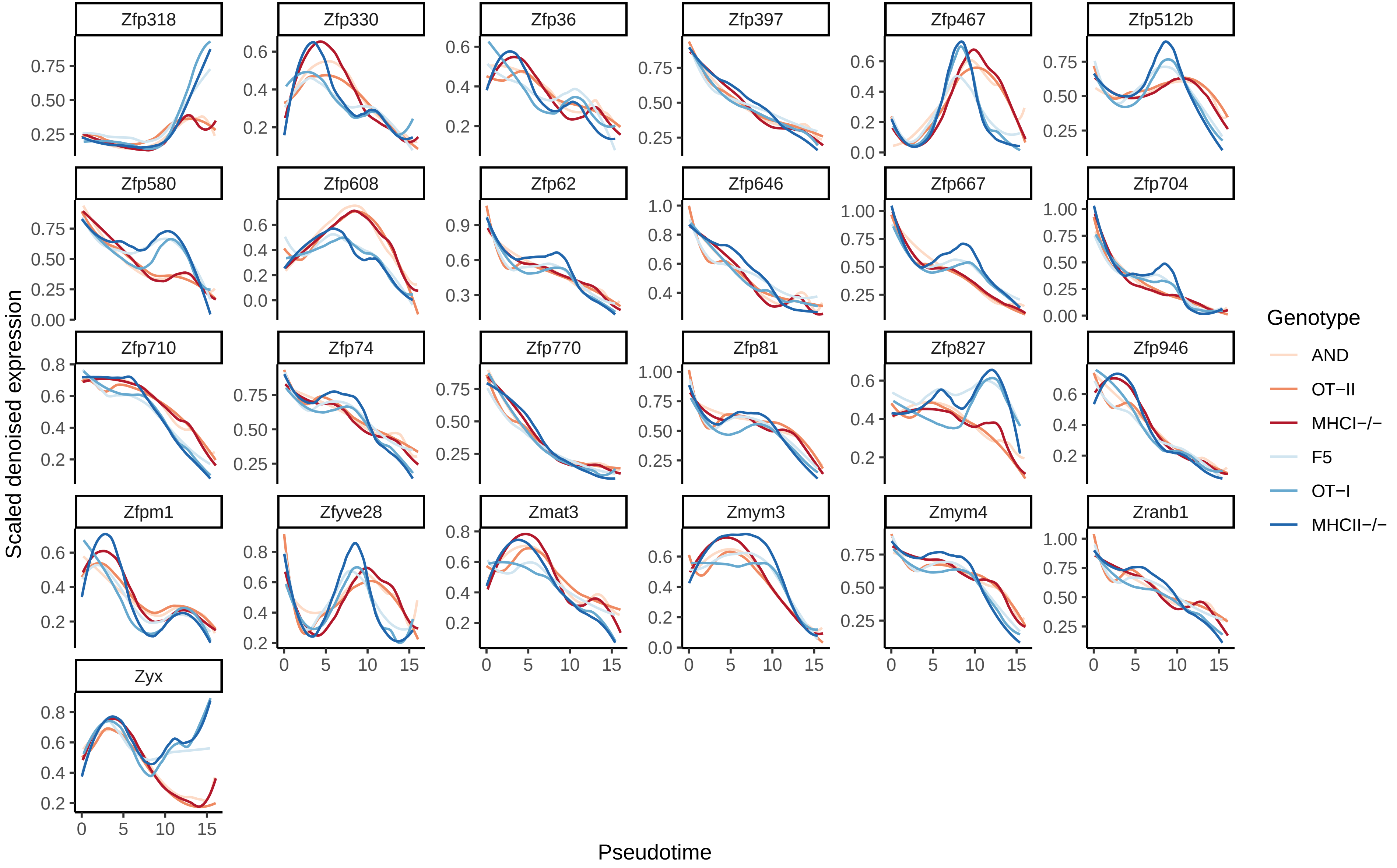

Zfp318
Zfp330
Zfp36
Zfp397
Zfp467
Zfp512b
Zfp580
Zfp608
Zfp62
Zfp646
Zfp667
Zfp704
Zfp710
Zfp74
Zfp770
Zfp81
Zfp827
Zfp946
Zfpm1
Zfyve28
Zmat3
Zmym3
Zmym4
Zranb1
Zyx
Scaled denoised expression
Pseudotime
Genotype
AND
OT−II
MHCI−/−
F5
OT−I
MHCII−/−
