# Supplementary material for: Single-cell multiomic analysis of thymocyte development reveals drivers of CD4+ T cell and CD8+ T cell lineage commitment
Source: Nat Immunol. 2023 Aug 14;24(9):1579–90. doi: 10.1038/s41590-023-01584-0 (PMC10457207; doi:10.1038/s41590-023-01584-0)
Supplement: Supplementary file 2 — Reporting Summary [file 41590_2023_1584_MOESM2_ESM.pdf]

nature portfolio

Corresponding author(s): Aaron Streets, Nir Yosef, Ellen Robey

Last updated by author(s): Jul 8, 2023

# Reporting Summary

Nature Portfolio wishes to improve the reproducibility of the work that we publish. This form provides structure for consistency and transparency in reporting. For further information on Nature Portfolio policies, see our Editorial Policies and the Editorial Policy Checklist.

## Statistics

For all statistical analyses, confirm that the following items are present in the figure legend, table legend, main text, or Methods section.

| n/a | Confirmed | |
|---|---|---|
| ☐ | ☒ | The exact sample size (*n*) for each experimental group/condition, given as a discrete number and unit of measurement |
| ☐ | ☒ | A statement on whether measurements were taken from distinct samples or whether the same sample was measured repeatedly |
| ☐ | ☒ | The statistical test(s) used AND whether they are one- or two-sided<br>*Only common tests should be described solely by name; describe more complex techniques in the Methods section.* |
| ☐ | ☒ | A description of all covariates tested |
| ☐ | ☒ | A description of any assumptions or corrections, such as tests of normality and adjustment for multiple comparisons |
| ☐ | ☒ | A full description of the statistical parameters including central tendency (e.g. means) or other basic estimates (e.g. regression coefficient) AND variation (e.g. standard deviation) or associated estimates of uncertainty (e.g. confidence intervals) |
| ☐ | ☒ | For null hypothesis testing, the test statistic (e.g. *F*, *t*, *r*) with confidence intervals, effect sizes, degrees of freedom and *P* value noted<br>*Give P values as exact values whenever suitable.* |
| ☐ | ☒ | For Bayesian analysis, information on the choice of priors and Markov chain Monte Carlo settings |
| ☒ | ☐ | For hierarchical and complex designs, identification of the appropriate level for tests and full reporting of outcomes |
| ☐ | ☒ | Estimates of effect sizes (e.g. Cohen's *d*, Pearson's *r*), indicating how they were calculated |

*Our web collection on statistics for biologists contains articles on many of the points above.*

## Software and code

Policy information about availability of computer code

| | |
|---|---|
| Data collection | No software was used in data collection. |
| Data analysis | 10x Genomics Cell Ranger v3.1.0<br><br>python 3.7.4<br>anndata==0.6.22.post1<br>leidenalg==0.7.0<br>matplotlib==3.0.3<br>numba==0.45.0<br>numpy==1.18.0<br>pandas==0.25.1<br>scanpy==1.4.5.post2<br>scipy==1.3.1<br>scvi==0.6.5<br>seaborn==0.9.0<br>sparse==0.8.0<br>statsmodels==0.10.1<br>torch==1.3.1<br>umap-learn==0.3.10<br>flowio==1.0.1<br><br>R 3.6.2<br>VISION==2.0.0 |

tidyverse==1.3.0
viridis==0.5.1
pals==1.6
RColorBrewer==1.1.2
scales==1.1.1
DescTools==0.99.36
pROC==1.16.2
slingshot==1.4.0
pheatmap==1.0.12
httr==1.4.1
jsonlite==1.7.2
readxl==1.3.1
openxlsx==4.1.5

Prism==10.0.0

Code is available at https://github.com/YosefLab/Thymus_CITE-seq and has been deposited at https://doi.org/10.5281/zenodo.8102050.

For manuscripts utilizing custom algorithms or software that are central to the research but not yet described in published literature, software must be made available to editors and reviewers. We strongly encourage code deposition in a community repository (e.g. GitHub). See the Nature Portfolio guidelines for submitting code & software for further information.

## Data

Policy information about availability of data

All manuscripts must include a data availability statement. This statement should provide the following information, where applicable:

- Accession codes, unique identifiers, or web links for publicly available datasets
- A description of any restrictions on data availability
- For clinical datasets or third party data, please ensure that the statement adheres to our policy

CITE-seq data discussed in this manuscript have been deposited in the National Center for Biotechnology Information's Gene Expression Omnibus and are accessible to reviewers and editors through accession number GSE186078.

# Field-specific reporting

Please select the one below that is the best fit for your research. If you are not sure, read the appropriate sections before making your selection.

☒ Life sciences ☐ Behavioural & social sciences ☐ Ecological, evolutionary & environmental sciences

For a reference copy of the document with all sections, see nature.com/documents/nr-reporting-summary-flat.pdf

# Life sciences study design

All studies must disclose on these points even when the disclosure is negative.

| | |
|---|---|
| Sample size | No sample size calculation was performed. The sample size was chosen based on availability of materials at the time of the study |
| Data exclusions | No data were excluded from the analysis. Filtering and quality control of single cell sequencing data is described in the Methods. |
| Replication | Experiments were performed on at least two biological replicates per genotype and condition to ensure reproducibility. All attempts at replication were successful. |
| Randomization | Randomization was not relevant to this study since all samples from each experiment were given the same treatment. |
| Blinding | Blinding was not relevant to this study since comparisons were objective and quantiative. For flow cytometry data, manual gates were applied uniformly to control and experimental samples. |

# Reporting for specific materials, systems and methods

We require information from authors about some types of materials, experimental systems and methods used in many studies. Here, indicate whether each material, system or method listed is relevant to your study. If you are not sure if a list item applies to your research, read the appropriate section before selecting a response.

## Materials & experimental systems

| n/a | Involved in the study |
| --- | --- |
| ☐ | ☒ Antibodies |
| ☒ | ☐ Eukaryotic cell lines |
| ☒ | ☐ Palaeontology and archaeology |
| ☐ | ☒ Animals and other organisms |
| ☒ | ☐ Human research participants |
| ☒ | ☐ Clinical data |
| ☒ | ☐ Dual use research of concern |

## Methods

| n/a | Involved in the study |
| --- | --- |
| ☒ | ☐ ChIP-seq |
| ☐ | ☒ Flow cytometry |
| ☒ | ☐ MRI-based neuroimaging |

# Antibodies

| | |
| --- | --- |
| Antibodies used | All antibodies used in this study are described in Supplementary Data 1, including the supplier, catalog number, and clone. |
| Validation | All antibodies used in this study were validated by the manufacturer. |

# Animals and other organisms

Policy information about studies involving animals; ARRIVE guidelines recommended for reporting animal research

| | |
| --- | --- |
| Laboratory animals | WT (B6) (C57BL/6, Stock No.: 000664), β2M-/- (B6.129P2-B2mtm1Unc/DcrJ, Stock No.: 002087; referred to as MHCI-/-), OT-I (C57BL/6-Tg(TcraTcrb)1100Mjb/J, Stock No.: 003831), and OT-II (B6.Cg-Tg(TcraTcrb)425Cbn/J, Stock No.: 004194) were obtained from The Jackson Laboratory. MHCII-/- (I-Aβ-/-) mice have been previously described (Grusby et al., 1991). AND TCRtg RAG1-/- mice and F5 TCRtg RAG1-/- mice were generated by crossing AND TCRtg (B10.Cg-Tg(TcrAND)53Hed/J, Jax Stock No.: 002761; (Kaye et al., 1989)) and F5 TCRtg (C57BL/6-Tg(CD2-TcraF5,CD2-TcrbF5)1Kio; (Mamalaki et al., 1992)) mice with RAG1-/- mice (Rag1-/-B6.129S7-Rag1tm1Mom) as previously described by (Au-Yeung et al., 2014)). All mice used in CITE-seq experiments were females between four and eight weeks of age. Samples are further described in Table S2. Mice were group housed with enrichment and segregated by sex in standard cages on ventilated racks at an ambient temperature of 26°C and 40% humidity. Mice were kept in a dark/light cycle of 12 h on and 12 h off and given access to food and water ad libitum. |
| Wild animals | This study did not involve wild animals. |
| Field-collected samples | This study did not involve samples collected from the field. |
| Ethics oversight | All animal care and procedures were carried out in accordance with guidelines approved by the Institutional Animal Care and Use Committees at the University of California, Berkeley and at BioLegend, Inc. |

Note that full information on the approval of the study protocol must also be provided in the manuscript.

# Flow Cytometry

## Plots

Confirm that:

☒ The axis labels state the marker and fluorochrome used (e.g. CD4-FITC).

☒ The axis scales are clearly visible. Include numbers along axes only for bottom left plot of group (a 'group' is an analysis of identical markers).

☒ All plots are contour plots with outliers or pseudocolor plots.

☒ A numerical value for number of cells or percentage (with statistics) is provided.

## Methodology

| | |
| --- | --- |
| Sample preparation | Cell preparation: Mice were sacrificed, and thymi were harvested, placed in RPMI + 10% FBS medium on ice, mechanically dissociated with a syringe plunger, and passed through a 70 µm strainer to generate a single-cell suspension. |
| Instrument | Flow cytometry data was collected using the BD LSRFortessa or BD LSRFortessa X20. |
| Software | Flow cytometry data was analyzed using FlowJo software (Tree Star). |
| Cell population abundance | All cell populations contain >200 cells. |
| Gating strategy | Cell gating strategies are described in supplemental methods. Single-stain samples and fluorescence minus one (FMO) controls were used to establish PMT voltages, gating and compensation parameters. |

☒ Tick this box to confirm that a figure exemplifying the gating strategy is provided in the Supplementary Information.
